# Microbial Biotransformation of the Sesquiterpene Carotol: Generation of Hydroxylated Metabolites with Potential Cytotoxic and Target-Specific Binding Activities

**DOI:** 10.3390/biom15121651

**Published:** 2025-11-26

**Authors:** Hanan G. Sary, Mohammed A. Khedr, Mohamed M. Radwan, Mickey Vinodh, Khaled Y. Orabi

**Affiliations:** 1 Department of Pharmaceutical Chemistry, College of Pharmacy, Kuwait University, Safat 13110, Kuwait or hanangaber@pharma.asu.edu.eg (H.G.S.); mohammed.khedr@ku.edu.kw (M.A.K.); 2 Department of Pharmacognosy, Faculty of Pharmacy, Ain-Shams University, Cairo 11566, Egypt; 3 Department of Pharmaceutical Chemistry, Faculty of Pharmacy, Helwan University, Ain Helwan, Cairo 11795, Egypt; 4 Department of Biomolecular Sciences, School of Pharmacy, The University of Mississippi, University, MS 38677, USA; mradwan@olemiss.edu; 5 National Center for Natural Products Research, School of Pharmacy, The University of Mississippi, University, MS 38677, USA; 6 Research Sector Project Units, Faculty of Science, Kuwait University, Safat 13060, Kuwait; miky.findo@ku.edu.kw

**Keywords:** carotol, *Absidia coerulea*, microbial biotransformation, sesquiterpene alcohol, hydroxydaucol, carrot seed essential oil, molecular docking, cytotoxic activity

## Abstract

Carotol, the major sesquiterpene alcohol in carrot essential oil, possesses notable cytotoxic activity against various cancer cell lines, yet its metabolic fate remains poorly understood. This study explored microbial biotransformation as a tool for generating novel carotol derivatives with potential pharmacological value. Seventeen microbial strains were screened, with *Absidia coerulea* ATCC 6647 identified as the most effective biocatalyst. Preparative-scale fermentation with this strain afforded three new metabolites, CM1, CM2, and CM3, in yields of 30%, 9.96%, and 3.28%, respectively, which were structurally characterized by 1D and 2D NMR, HRMS, and single-crystal X-ray diffraction. These were identified as 9α-hydroxydaucol (CM1), 9α,13-dihydroxydaucol (CM2), and a diol derivative of daucol (CM3). Cytotoxicity evaluation against human carcinoma cell lines (HepG-2, HCT-116, MCF-7, A-549) and normal lung fibroblasts (MRC-5) revealed that carotol exhibited notable activity with IC_50_ values of 25.68 and 28.65 µM against HCT-116 and A-549 cell lines, respectively. Among the metabolites, CM2 showed selective cytotoxicity with IC_50_ values of 180.64 (HCT-116) and 138.21 µM (A-549), indicating that microbial transformation modulates the cytotoxic profile of carotol and yields metabolites with distinct bioactivity patterns. Molecular docking studies further revealed that carotol and CM2 demonstrated higher binding affinities and more stable interactions with human NADPH oxidase, suggesting that inhibition of this enzyme may underlie their cytotoxic effects. This work provides the first detailed microbial biotransformation pathway of carotol, highlighting *A. coerulea* as a promising source of new hydroxylated metabolites. The results underscore the potential of carotol derivatives in anticancer drug development and warrant further pharmacokinetic studies.

## 1. Introduction

The carrot (*Daucus carota* L. subsp. *carota*, Family Apiaceae), a biennial flowering plant native to Europe, Asia, and Africa, ranks among the ten most economically significant vegetable crops worldwide [1]. Carrots provide substantial nutritional and health benefits due to their abundant carotenoids, phenolics, vitamin C, and polyacetylenes, which contribute to reduced risks of cardiovascular disease and cancer [2]. Phytochemical-rich diets, including carrots, help prevent cancer, heart disease and metabolic disorders [3,4,5,6]. Additionally, *D. carota* extracts are used for hepatic insufficiency and wound healing [7,8,9], and exhibit iron-chelating, antioxidant [10], and antibacterial activities [11]. Carrot seed essential oil is widely utilized in food flavoring, perfumes, cosmetics, and soaps [10,11,12] demonstrating antimicrobial, antioxidant, anti-aging, cytotoxic, and mosquito-repellent properties [13,14,15,16,17,18,19,20]. Carotol, a sesquiterpene alcohol, is the predominant compound in carrot seed oil [12,21,22,23], displaying strong antifungal [19,24], cytotoxic [12], antioxidant, anti-inflammatory [23] and mosquito-deterrent activities [15].

Drug metabolism studies traditionally rely on in vivo animal models or in vitro enzyme-based systems, such as microsomal preparations and tissue cultures. Microbial biotransformation provides an alternative, overcoming certain limitations of traditional methods. The concept of “microbial models of mammalian metabolism” involves using bacteria, yeasts, and fungi to simulate human drug metabolism. Microbial systems serve as efficient, selective, and scalable tools for generating metabolites that closely resemble those produced in mammalian systems [25]. Their advantages include operational efficiency under mild conditions and the ability to catalyze diverse reactions beyond their natural substrates while exhibiting chemo-, regio-, and stereoselectivity [26]. These models facilitate metabolite production in quantities unattainable from animal-based systems or chemical synthesis, making them predictive tools for human metabolism.

Fungi, as eukaryotes, share enzymatic similarities with mammals, reinforcing their role as models for mammalian metabolism. It has been reported that different fungi are capable of modifying the structure of sesquiterpenoids via various reactions such as hydroxylation, hydrogenation, acylation, methoxylation and epoxidation [27,28,29]. Previous studies have reported carotol biotransformation by *Rhizopus oryzae*, yielding two lactate derivatives: 9*α*-hydroxydaucol-9-lactate and 9*α*-hydroxydaucol-3,9-dilactate [30]. These metabolites exhibited in vitro inhibition of cyclooxygenase-2 (COX-2), 5-lipoxygenase (5-LOX), and butyrylcholinesterase (BChE) [30]. Furthermore, carotol chemical modifications have yielded 3-bromcarotol ether, carotol aldehyde, 4-phenoxy carotol, and daucene [31], ketone mixtures [32], and carota-1,4-dienaldehyde [33]. To date, no carotol metabolites of human or mammalian origin have been reported.

This study investigates the microbial biotransformation of carotol to generate novel derivatives in quantities sufficient for cytotoxicity assessment, with the aim of supporting their potential therapeutic applications. Furthermore, molecular docking analyses were performed to compare the binding interactions of the newly formed metabolites and carotol with NADPH oxidase, an enzyme implicated as a therapeutic target in cancer treatment strategies.

## 2. Materials and Methods

### 2.1. Substrate Preparation

Pure carotol (**1**) was obtained from carrot essential oil as previously described [15]. Briefly, therapeutic-grade carrot essential oil was purchased from Edens Garden (1322 Calle Avanzado, San Clemente, CA, USA). Successive chromatographic purification yielded a carotol-rich fraction, which was further purified using preparative high-performance liquid chromatography (HPLC) to obtain pure carotol.

### 2.2. Microorganisms

The microbial cultures were sourced from the American Type Culture Collection (ATCC, Rockville, MD, USA), the Northern Regional Research Laboratories (NRRL), and Assiut University Mycology Center (AUMC). The following microorganisms were screened for their ability to catalyze the biotransformation of carotol: *Absidia coerulea* ATCC 6647, *Fusarium* sp. ATCC 11599, *Phanerochaete chrysosporium* ATCC 24725, *Rhizopus oryzae* (syn. *R. nigricans*) ATCC 34121, *Rhizopus stolonifer* ATCC 6227a, *Aspergillus flavus* AUMC 4787, *Aspergillus niger* NRRL 599, *Cunninghamella blacksleeana* AUMC, *Cunninghamella echinulata* NRRL 132, *Mucor* sp. AUMC, *Rhizopus* sp. ATCC 36060, *Saccharomyces cerevisiae* (Baker’s yeast), *Cladosporium* sp., *Cordyceps sinensis* AUMC, *Botrytis allii* ATCC 9435, *Streptomyces griseus* ATCC 13968, *Streptomyces spheroids* ATCC 23965. Stock cultures were maintained on agar slants using media recommended by ATCC and stored at 4 °C.

### 2.3. General Experimental Procedure

IR spectra were recorded as a chloroform film using Jasco FT/IR-4100 type A Spectrophotometer (Tokyo 192-8537, Japan). The ^1^H and ^13^C NMR spectra were obtained on a Bruker Avance II-600 spectrometer (Ettlingen, Germany) operating at 600 and 150 MHz, respectively. Both ^1^H and ^13^C NMR spectra were recorded in CDCl_3_, and the chemical shift values were expressed in *δ* (ppm) relative to the internal standard TMS. For the ^13^C NMR spectra, spectral editing was determined by DEPT. Two-dimensional NMR data were obtained using the standard pulse sequence of the Bruker Avance II-600 (Ettlingen, Germany) for COSY, HSQC and HMBC. EIHRMS analysis were carried out on a Thermo Scientific High-Resolution GC/MS-DFS (Double Focusing Sector) mass spectrometer (Thermo Fisher Scientific, Bremen, Germany). Column chromatography was carried out on Silica gel 60 (230–400 mesh ASTM, Merck, Darmstadt, Germany). TLC analysis was carried out on silica gel 60 F254 (Merck) plates. Compounds were detected by UV and *p*-anisaldehyde/H_2_SO_4_ spraying reagent followed by heating at 105 °C for 1–2 min.

### 2.4. Preparative HPLC

A Waters preparative HPLC system, equipped with a 2998 Photodiode Array detector, a 2545 quaternary gradient module, a FlexInject manual dual injector, and a Fraction Collector III, was used to isolate pure carotol. The separation was performed on a Waters XBridge^TM^ Prep C18 (10 × 150 mm, 5 µm) column using isocratic elution with 30% methanol in acetonitrile at a flow rate of 1.7 mL/min. The detection wavelength was set at 218 nm. The sample was prepared by dissolving 100 µL of carotol-rich fraction in 1.5 mL of 30% methanol in acetonitrile, filtered through a 0.1 µm PTFE membrane, and injected into the system.

### 2.5. Screening Procedure

The microbial screening was conducted following a standard two-stage protocol [25,34]. Fermentation medium alpha was prepared using 2% glucose, 0.5% yeast extract, 0.5% peptone, 0.5% NaCl, and 0.5% K_2_HPO_4_ in distilled water, sterilized at 121 °C for 15 min. In Stage I, 250 mL culture flasks containing 50 mL of sterile medium alpha were inoculated with the selected microbial strains and incubated at 150 rpm for 72 h at room temperature. Stage II involved transferring 5 mL of the Stage I culture to fresh medium, followed by 24 h of incubation before substrate addition. Carotol (**1**) was prepared as a 15% solution in *N,N*-dimethylformamide (DMF) and added to Stage II cultures at a final concentration of 0.5 mg/mL, then the cultures were incubated at 5 °C for two weeks.

Control groups included a substrate control, in which sterile medium containing carotol (5 mg/100 µL DMF) was incubated without microorganisms. The substrate control maintained at 5 °C remained fully stable throughout the two-week fermentation period, whereas the one incubated at room temperature (25–30 °C) showed slight decomposition of carotol over time. Culture controls consisted of microorganisms grown under identical conditions to the screening cultures but without carotol. After two weeks of incubation, all cultures were harvested and analyzed by thin-layer chromatography.

### 2.6. Preparative-Scale Fermentation

Since *Absidia coerulea* ATCC 6647 exhibited clear capability to metabolize carotol, it was cultured in five 250 mL flasks containing 50 mL of medium alpha (Stage I). A total of 304 mg of carotol, dissolved in 450 µL DMF, was distributed among nine 1 L flasks containing 24 h old Stage II cultures. After 14 days, the cultures were analyzed using TLC with an *n*-hexane–acetone (8.5:1.5) solvent system. Visualization with *p*-anisaldehyde/H_2_SO_4_ revealed the transformation of carotol (**1**) into three polar metabolites.

Each culture flask was filtered, and the filtrates were extracted with chloroform (1.5 L × 3). The combined extracts were dried over anhydrous Na_2_SO_4_ and evaporated to dryness under reduced pressure at 38 °C, yielding a brownish residue (525 mg). Purification was performed using flash silica gel column chromatography (68 g, 2.7 × 33 cm, 230–400 mesh) with CHCl_3_–acetone (9.3:0.7) as the eluent. Fractions were collected (25 mL each) and pooled based on TLC profile similarity.

### 2.7. Single Crystal X-Ray Diffraction Analysis

Single crystals of CM1 used in this study were obtained via slow solvent (chloroform in methanol) evaporation. X-ray diffraction data were collected at 150 K using a Bruker X8 Prospector diffractometer (Bruker Corporation, Billerica, MA, USA) equipped with Cu-Kα radiation. Reflection frames were integrated using the Bruker SAINT (SAINT V8.40A, Bruker Nano Inc., Billerica, MA, USA, 2019) software package with a narrow-frame algorithm. The crystal structure was solved with the SHELXT software 2014/5 suite and refined using SHELXL-2017/1. All non-hydrogen atoms were refined anisotropically, while hydrogen atoms were placed in calculated positions and refined using a riding model.

### 2.8. Cytotoxicity Evaluation

The cytotoxic activities of pure carotol (**1**) and its metabolites were evaluated against human liver (HepG-2), colon (HCT-116), breast (MCF-7), and lung (A-549) carcinoma cell lines, as well as normal human lung fibroblasts (MRC-5), using a standard cell viability (MTT) assay [35], and *cis*-platin and doxorubicin as positive controls. The selection of the carcinoma cell lines for evaluating the cytotoxicity of carotol (**1**), and its metabolites was based on the properties and traditional uses of *D. carota* extracts and essential oils. *D. carota* is consumed as a food and utilized in perfumes, furthermore, studies indicated that carrot intake lowers the risk of breast cancer and lung cancer in smokers [36,37]. Evaluating the effects of carotol (**1**) and its metabolites on HCT-116, MCF-7 and A-549 cell lines provides insight into their absorption, and assesses their potential as treatments for colon, breast, and lung cancers. The HepG-2 cell line was included to validate the traditional use of carrots in hepatic insufficiency [7] and to examine their effects on liver cancer cells. The assay was conducted at the Regional Center for Mycology and Biotechnology, Al-Azhar University, Cairo, Egypt.

Cancer and normal cells were seeded into 96-well plates at a density of 5 × 10^4^ cells per well and incubated for 24 h prior to treatment. Test compounds, dissolved in 0.5% DMSO, were added in triplicate at twelve different concentrations. Vehicle controls (media only or media containing 0.5% DMSO) were included. After a 24 h incubation period, cell viability was assessed via the MTT assay.

Briefly, the media were replaced with 100 µL of fresh RPMI-1640 medium, followed by the addition of 10 µL of a 12 mM MTT stock solution (5 mg/mL in PBS) to each well, including untreated controls. Plates were incubated at 37 °C in a 5% CO_2_ atmosphere for 4 h. Subsequently, 85 µL of medium was removed from each well, and 50 µL of DMSO was added to dissolve the resulting formazan crystals. The plates were thoroughly mixed and incubated for an additional 10 min at 37 °C. Absorbance was measured at 590 nm using a microplate reader (SunRise, TECAN Inc., Morrisville, NC, USA). Cell viability percentages were calculated, and IC_50_ values (µM) were determined from dose–response curves using GraphPad Prism 10 software (San Diego, CA, USA) [38].

### 2.9. Docking Study

The crystal structure of human NADPH oxidase was downloaded from the protein data bank (pdb code = 8gz3) [39]. The structure was published in 2022, with resolution = 3.3 Å. Molecular docking was performed using a Molecular Operating Environment (MOE 2022.02) package license purchased from Chemical Computing Group Inc., Sherbooke St, Montreal, QC, Canada. Triangle matcher was used as a placement method. London ΔG scoring method was applied to estimate the free energy of binding (Kcal/mol). Root mean square deviation (RMSD) of the docked ligands was computed to measure the deviation from the co-crystalized ligand. The root mean square fluctuation (RMSF) was computed to compare between carotol and its metabolites.

### 2.10. Statistical Analysis

Statistical analyses were performed using SPSS software version 22.0 (IBM Corp., Armonk, NY, USA). Data are presented as mean ± standard deviation (SD). Group differences were assessed using one-way analysis of variance (ANOVA), followed by Tukey’s post hoc test. A *p*-value < 0.05 was considered statistically significant.

## 3. Results and Discussion

**Figure 1:**
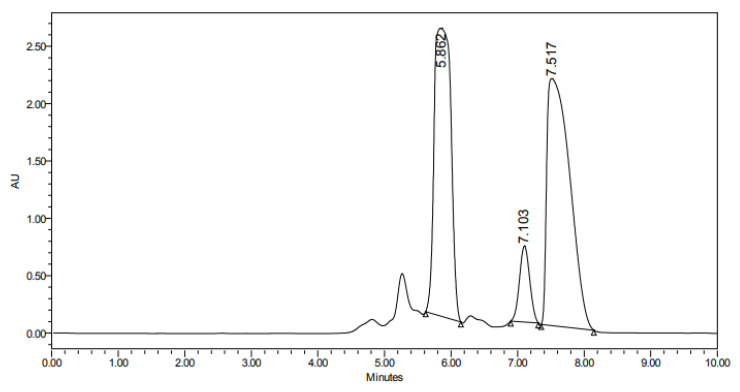
Chromatogram depicting the separation of two major compounds. Car-2 (t_R_ = 7.5 min) was purified and identified as pure carotol based on its NMR spectra.

Preparative HPLC fractionation resulted in the isolation of two major compounds (Figure 1), designated as Car-1 (t_R_ = 5.9 min) and Car-2 (t_R_ = 7.5 min). One-dimensional- and two-dimensional-NMR spectral analysis confirmed that Car-2 corresponds to pure carotol (Figure S1).

With the exception of a single reference [30], a comprehensive literature search revealed no prior documentation or investigation detailing either microbial or mammalian metabolic pathways of carotol. Although carotol has been previously investigated for its biological activities, including cytotoxic and antimicrobial properties, its metabolic fate, particularly via microbial biotransformation, remains largely unexplored. The current findings thus provide novel insights into the metabolism of carotol, laying the foundation for future pharmacokinetic and bioactivity studies, and underscoring the value of microbial systems in elucidating the biotransformation pathways of bioactive sesquiterpenes.

**Figure 2:**
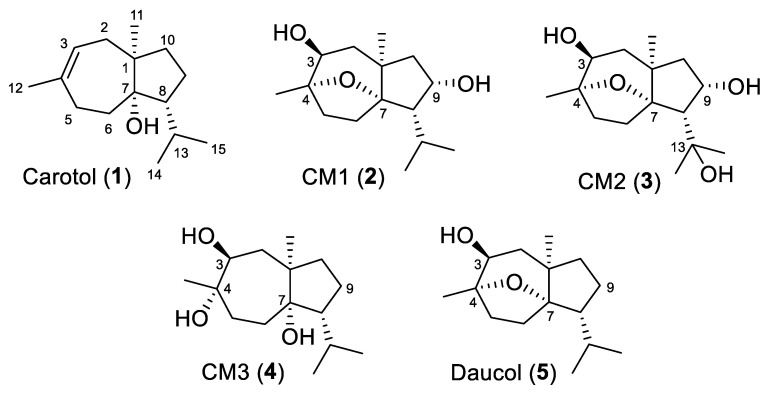
Structures of carotol, its metabolites and daucol.

The metabolic capabilities of seventeen microbial cultures were assessed for carotol biotransformation, and *Absidia coerulea* ATCC 6647 exhibited significant metabolic activity (Figure S2). Consequently, this strain was selected for preparative-scale fermentation, yielding three metabolites: CM1, CM2, and CM3 (Figure 2). The metabolites were purified using column chromatography, with multiple fractions collected and analyzed by TLC. Fractions 19, 21, and 25 were identified as promising based on their TLC profiles compared to the culture control.

Fraction 19 (123 mg) was subjected to further purification over a silica gel 60 column (17 g, 30 × 1.5 cm) with toluene–acetone (9:1) as the mobile phase. Fractions (5 mL each) were collected and analyzed by TLC using the same solvent system. Similar fractions were pooled, affording a pure, crystalline, non-UV-active compound, designated CM1 (**2**) (104.3 mg, 30% yield), with R*_f_* = 0.26 (toluene–acetone; 8.5:1.5).

Additionally, fraction 25, with no further purification, yielded 36.8 mg (9.96% yield) of pure, non-UV-active amorphous powder, designated CM2 (**3**) with an R*_f_* value = 0.27 (toluene–acetone; 8.5:1.5).

Moreover, fraction 21 (30.5 mg) was purified using a silica gel column (15 × 1.4 cm, 6.4 g) and eluted with toluene–acetone (8.7:1.3). Fractions (2 mL each) were collected and monitored by TLC using the same mobile phase. Similar fractions were pooled, yielding CM3 (**4**) (11.5 mg, 3.28% yield) as a pure, non-UV-active amorphous powder. The R*_f_* value was 0.27 in toluene-acetone (9:1).

The yields of CM2 and CM3 were low; however, this is not uncommon in microbial biotransformation studies, particularly when using secondary metabolites such as carotol, which may exhibit limited solubility and bioavailability within the fermentation medium. Moreover, similar low to moderate yields (4–10%) have been reported in the microbial transformations of sesquiterpenes such as vulgarin [40,41]. These comparisons further support that the transformation pattern and yield range observed in our study are in good agreement with the expected metabolic behavior of carotol under standard two-stage fermentation conditions.

CM1 (**2**) structure was characterized based on detailed spectroscopic analysis. Its molecular formula was established as C_15_H_26_O_3_, supported by the molecular ion peak at *m*/*z* 254.1873, along with its ^1^H and ^13^C NMR data (Table 1 and Table 2, and Figure S1). The ^13^C NMR spectrum exhibited 15 resonances, categorized as three singlets, four doublets, four triplets, and four quartets. Compared to carotol, CM1 lacked the characteristic olefinic signals observed at δ_C_ 138.8 (singlet) and δ_C_ 122.3 (doublet), indicating the absence of the double bond present in the parent compound.

Conversely, CM1 displayed four oxygenated aliphatic carbon signals at δ_C_ 71.5 (doublet, C-3), δ_C_ 85.6 (singlet, C-4), δ_C_ 92.6 (singlet, C-7), and δ_C_ 73.3 (doublet, C-9). Signal assignments were confirmed through 2D NMR experiments (HSQC and HMBC), which enabled unambiguous correlation of proton and carbon resonances. Notably, several of these chemical shifts closely matched those reported for daucol (**5**), a major sesquiterpene constituent of carrot seed oil [19]. The identity and absolute configuration of CM1 were further confirmed by single crystal X-ray diffraction.

**Figure 3:**
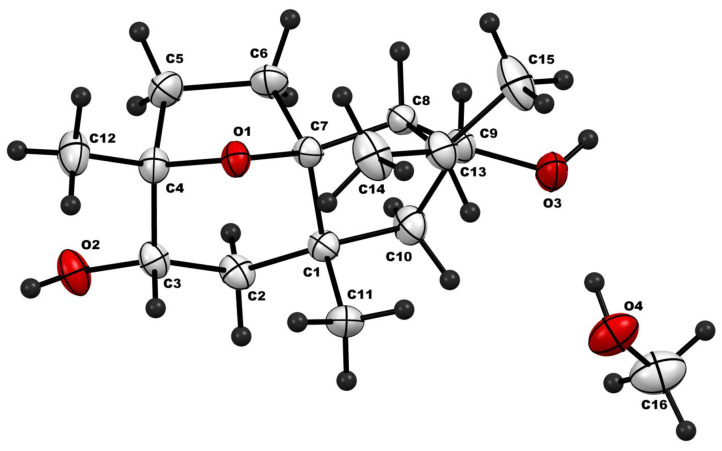
Crystal structure (thermal ellipsoid representation; 30% probability) of CM1 obtained from single crystal diffraction. Color code: gray—carbon; red—oxygen; and black—hydrogen.

The crystal structure of CM1 obtained from single crystal diffraction analysis is depicted in Figure 3, and important crystallographic parameters of this crystal are provided in Table 3.

**Figure 4:**
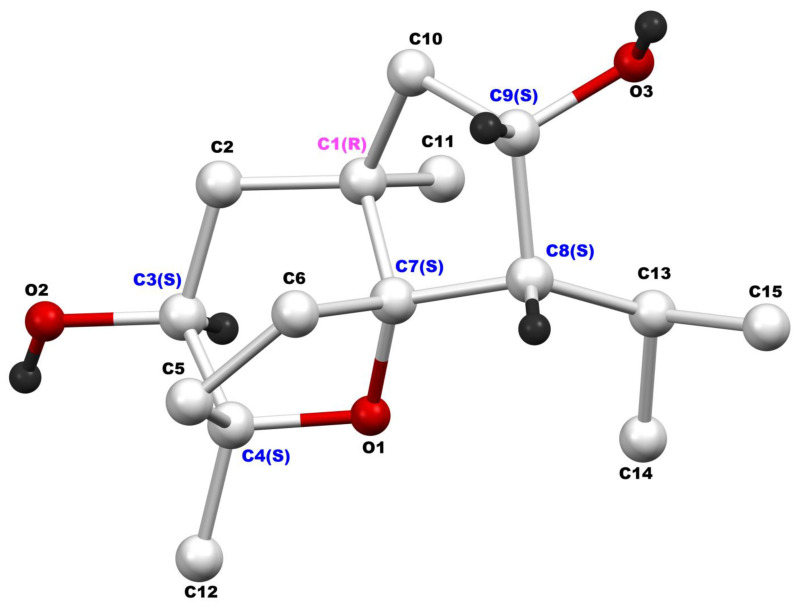
Crystal structure of CM1 in which the chiral atoms are color-labeled.

CM1 crystallizes in the orthorhombic crystal system, space group *P*2_1_2_1_2_1_. Methanol molecules are incorporated into the crystal lattice as space-filling solvents, stabilizing the crystal structure through non-bonding interactions with adjacent CM1 molecules. The crystal structure of CM1 reveals the presence of six chiral carbon atoms in the molecule. These chiral centers include C-1 (*R*), C-3 (*S*), C-4 (*S*), C-7 (*S*), C-8 (*S*), and C-9 (*S*), which are color-labeled in Figure 4. Thus, CM1 identity was established as the novel 9α-hydroxydaucol (**2**) (Figure 2).

On the other hand, CM2 (**3**) was determined to have the molecular formula C_15_H_26_O_4_, as established by its molecular ion peak at *m*/*z* 270.1826, supported by its ^1^H and ^13^C NMR data (Table 1 and Table 2, and Figure S1). The ^13^C NMR spectrum displayed 15 carbon resonances, comprising four singlets, three doublets, four triplets, and four quartets. Five of these resonances were clearly in the oxygenated aliphatic region. Comparison with the NMR data of CM1 revealed the presence of an additional oxygenated carbon in CM2, resonating at δ_C_ 74.1 as a singlet, and the absence of the doublet observed in CM1 at δ_C_ 29.4 (assigned to C-13). This suggested that the additional oxygen functionality in CM2 was located at C-13. This assignment was further supported by downfield chemical shifts of the adjacent methyl carbons, C-14 (δ_C_ 31.1, Δ = 4.9 ppm) and C-15 (δ_C_ 30.2, Δ = 7.5 ppm), indicating deshielding due to proximity to the oxygenated center. The remaining spectral data were consistent with those observed for CM1 (Table 1 and Table 2). Based on these findings, the structure of CM2 was established as the novel 9α,13-dihydroxydaucol (Figure 2).

Likewise, CM3 (**4**) was shown to have a molecular formula of C_15_H_28_O_3_ based on its HRMS that shows the molecular ion peak at *m*/*z* 256.2032 and its ^1^H and ^13^C NMR data. Again, there were 15 carbon resonances distributed as three singlets, three doublets, five triplets, and four quarters. Of these resonances, three appear in the oxygenated aliphatic region at δ_C_ 75.4 (doublet, C-3), δ_C_ 75.5 (singlet, C-4), and δ_C_ 83.6 (singlet, C-7). Comparison of the ^13^C NMR data of CM3 with those published for daucol indicates that CM3 is the new diol derivative resulting from the cleavage of the daucol ether linkage (Figure 2). Notably, CM3 is confirmed as a true microbial metabolite, as pure carotol was used as the sole substrate in the microbial cultures. The unambiguous assignment of all ^1^H and ^13^C resonances was aided through DEPT 135°, HSQC, and HMBC experiments (Figure S1).

CM1 (**2**): colorless prims (MeOH): mp 122–124 °C; IR (neat) υ_max_ 3434 (OH), 2935 (saturated C-H) cm^−1^; ^1^H NMR (CDCl_3_, 600 MHz) see Table 1 and Figure S1; ^13^C NMR (CDCl_3_, 150 MHz) see Table 2 and Figure S1; EIHRMS *m*/*z* 254.1873 [M]^+^ (calcd for C_15_H_26_O_3_ 254.1882).

CM2 (**3**): colorless amorphous powder; IR (neat) υ_max_ 3386 (OH), 2935 (saturated C-H) cm^−1^; ^1^H NMR (CDCl_3_, 600 MHz) see Table 1 and Figure S1; ^13^C NMR (CDCl_3_, 150 MHz) see Table 2 and Figure S1; HRMS *m*/*z* 270.1826 [M]^+^ (calcd for C_15_H_26_O_4_ 270.1831).

CM3 (**4**): gummy residue; IR (neat) υ_max_ 3380 (OH), 2910 (saturated C-H) cm^−1^; ^1^H NMR (CDCl_3_, 600 MHz) see Table 1 and Figure S1; ^13^C NMR (CDCl_3_, 150 MHz) see Table 2 and Figure S1; HRMS *m*/*z* 256.2032 [M]^+^ (calcd for C_15_H_28_O_3_ 256.2038).

The metabolites obtained in this study (CM1–CM3) differ structurally from those described by Soliman et al. [30], who reported the production of sesquiterpene lactate esters. Our findings instead demonstrate regioselective hydroxylation of the parent alcohol, suggesting the involvement of a different enzymatic system and biotransformation mechanism. This highlights the microbial strain-dependent variability in carotol metabolism.

The cytotoxic effects of carotol and its metabolites were evaluated against several cancer cell lines (HepG2, HCT-116, MCF-7, and A549) as well as the normal human lung fibroblast cell line (MRC-5), using cis-platin (Figure S3), and doxorubicin as positive controls. Among the tested compounds, the parent compound, carotol, exhibited the highest cytotoxic potency among the tested compounds, with IC_50_ values ranging from 25.68 to 52.34 µM across the four cancer cell lines (HepG-2, HCT-116, MCF-7, and A-549) as shown in Table 4. Statistical analysis (one-way ANOVA followed by Tukey’s post hoc test) revealed that its metabolites (CM1, CM2, and CM3) demonstrated significantly weaker cytotoxic effects (*p* < 0.05–0.001 vs. carotol), indicating that oxidative biotransformation reduces the intrinsic cytotoxic potential of the parent compound. The reference drugs cis-platin and doxorubicin exhibited much stronger cytotoxicity (*p* < 0.01–0.001), and lower toxicity toward normal MRC-5 fibroblasts, resulting in higher selectivity indices. In contrast, carotol and its metabolites displayed moderate to low selectivity (SI = 1.3–6.8), with carotol followed by CM1 displaying the highest SI in this series of compounds, suggesting a more favorable safety profile and potential for selective cytotoxicity. Carotol’s superior balance between potency and selectivity supports its potential as a promising anticancer lead compound for further optimization.

When compared with the positive controls, cis-platin and doxorubicin, carotol demonstrated significantly lower cytotoxic potency (*p* < 0.01–0.001) toward cancer cells; however, this reduced potency was accompanied by a markedly lower toxicity to normal cells (*p* < 0.05 vs. controls). This distinction resulted in higher selectivity indices for carotol relative to its metabolites and comparable selectivity to that of cis-platin, indicating that its cytotoxicity may arise from a more selective, regulated mechanism rather than broad nonspecific cell damage. While cis-platin and doxorubicin remain potent chemotherapeutics, their limited selectivity (SI = 6.4–14.7) reflects their well-known systemic toxicity. By contrast, carotol’s milder yet selective cytotoxic profile, in particular against HepG-2 and MCF-7 cells, positions it as a valuable scaffold for the design of safer anticancer agents. The confidence intervals indicate reproducible results across assays (Table S1).

Previous studies have reported the cytotoxicity of carrot seed essential oils from various geographical sources, along with pure carotol, using green monkey kidney epithelial cells (VERO) and human hypopharyngeal squamous cell carcinoma cells (FaDu). Moroccan and French essential oils exhibited comparable cytotoxic effects, while the Polish essential oil displayed lower activity. In contrast, pure carotol demonstrated moderate cytotoxicity on both VERO and FaDu cell lines, with no apparent selectivity (IC_50_ = 39.7 µg/mL and 38.3 µg/mL, respectively) [12].

It has been previously documented that sesquiterpene alcohols can act as inhibitors of NADPH oxidase, an enzyme implicated in oxidative stress and cancer progression. The inhibition of NADPH oxidase is regarded as a key mechanism contributing to the anticancer potential of this class of compounds [42]. In the present study, molecular docking simulations were performed to assess the binding interactions, free binding energy (ΔG), and binding stability of carotol and its metabolites with NADPH oxidase. As shown in Table 5, carotol exhibited the most favorable binding profile, with the highest binding affinity (ΔG = −5.65 kcal/mol) and the lowest RMSD value (1.35 Å), indicating a stable interaction. CM2 followed with a ΔG of −5.41 kcal/mol and an RMSD of 1.50 Å. In contrast, CM1 and CM3 showed lower binding affinities (ΔG = −5.11 and −5.16 kcal/mol, respectively), suggesting weaker interactions with the enzyme. These computational findings are in good agreement with the experimental cytotoxicity data presented in Table 4 and further support the hypothesis that carotol and CM2 exert anticancer effects, at least in part, through NADPH oxidase inhibition.

**Figure 5:**
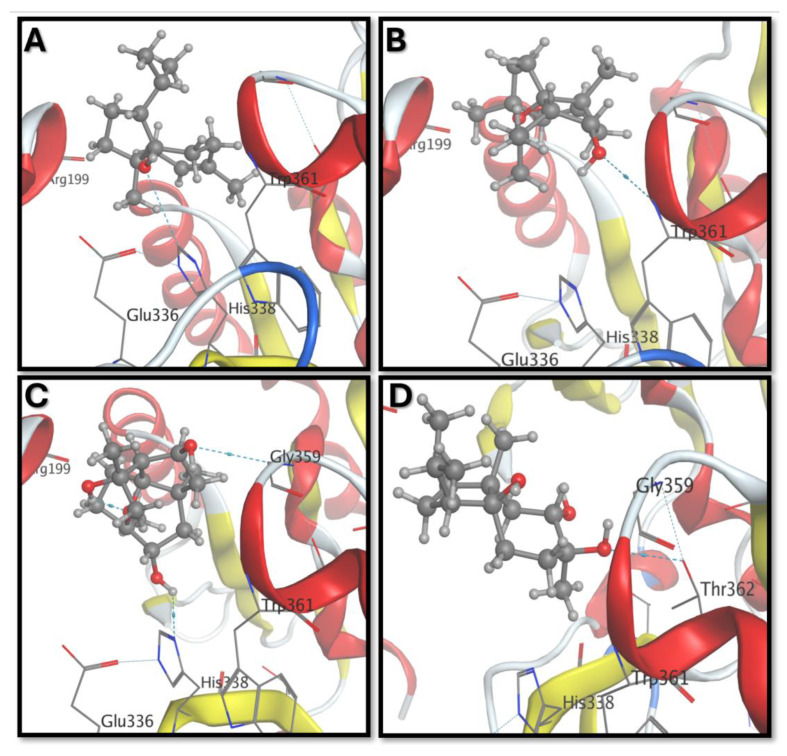
Docking interactions of NADPH oxidase with: (**A**) Carotol, (**B**) CM1, (**C**) CM2, and (**D**) CM3. Hydrogen bonds and key interacting residues are highlighted to illustrate binding modes of each compound.

Further analysis of the molecular docking interactions revealed distinct binding modes for carotol and its metabolites with NADPH oxidase (Figure 5). Carotol formed a hydrogen bond with His338 and exhibited potential hydrophobic interactions with Trp361 (Figure 5A), suggesting a stable and favorable binding conformation. CM1 established a single hydrogen bond with the –NH group of Trp361 (Figure 5B), indicating a moderately stable interaction. Notably, CM2 was the only metabolite to exhibit an intramolecular hydrogen bond, which may contribute to enhanced binding stability. Additionally, CM2 formed two hydrogen bonds with Gly359 and His338 (Figure 5C), reinforcing its strong binding affinity. In contrast, CM3 interacted via a single hydrogen bond with Thr362 (Figure 5D), suggesting a comparatively weaker interaction. These distinct binding interactions further support the differential binding affinities observed in the docking scores and correlate well with the experimental cytotoxicity data.

**Figure 6:**
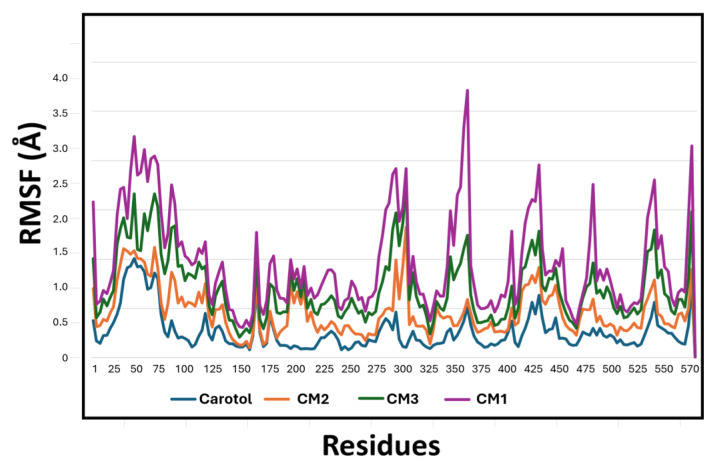
Root mean square fluctuations (RMSFs) of NADPH oxidase residues in complex with carotol, CM1, CM2, and CM3 over a 50 ns molecular dynamics simulation. The RMSF profiles illustrate the residue-wise flexibility upon binding of each compound, providing insight into the stability of the protein–ligand complexes.

To further investigate the stability of binding interactions with NADPH oxidase, molecular dynamics (MD) simulations were performed over a 50 ns timescale for the best-docked complexes of carotol and its metabolites (CM1, CM2, and CM3). The resulting trajectories were analyzed to compute the root mean square fluctuations (RMSFs) of NADPH oxidase residues in each complex, providing insight into local flexibility and binding stability (Figure 6). Carotol exhibited the lowest RMSF values (blue trace), indicating a highly stable interaction with the enzyme throughout the simulation. CM2 displayed RMSF values comparable to those of carotol (orange trace), supporting its strong and stable binding. In contrast, CM1 (purple trace) showed higher fluctuations relative to carotol and CM2, suggesting a less stable interaction. The metabolite CM3 (green trace) demonstrated the highest residue fluctuations, with RMSF values exceeding 3.5 Å in certain regions, indicating the least stable binding among the tested compounds. These findings align well with the cytotoxicity results and further support the superior stability and potential biological activity of CM2 relative to the other metabolites.

The observed cytotoxicity of carotol and its metabolites may be linked to reactive oxygen species (ROS) generation and disruption of mitochondrial membrane potential, mechanisms frequently associated with sesquiterpene alcohols. Moreover, molecular docking and molecular dynamics simulations against NADPH oxidase revealed that carotol and CM2 form stable complexes with the enzyme’s catalytic pocket, supporting a possible role in oxidative stress modulation. This mechanistic insight aligns with the experimental findings, reinforcing carotol’s selective cytotoxic behavior through redox-mediated pathways rather than direct DNA alkylation typical of cisplatin and doxorubicin. These results provide supportive yet preliminary evidence suggesting a possible involvement of NADPH oxidase in the biological activity of the metabolites, warranting further in vitro validation in future studies. Although the molecular docking data indicate a potential interaction between carotol and its metabolites and NADPH oxidase, this finding should be regarded as a plausible mechanistic hypothesis rather than a confirmed causal relationship. It is worth mentioning that previous studies have demonstrated that sesquiterpene alcohols, e.g., cedrol, can inhibit NADPH oxidase and attenuate oxidative stress [42]; therefore, a similar interaction may contribute, at least in part, to the cytotoxic effects observed in this study. Nevertheless, additional biochemical and enzymatic investigations are required to substantiate this proposed mechanism. Overall, the integrated biological, statistical, and computational results identify carotol and its metabolites as promising natural leads for further anticancer optimization and mechanistic exploration.

## 4. Conclusions

This study successfully isolated pure carotol from carrot essential oil and evaluated its microbial biotransformation by diverse microorganisms. Among the strains tested, *Absidia coerulea* ATCC 6647 demonstrated significant metabolic activity, producing three novel distinct metabolites (CM1, CM2, and CM3). Structural elucidation of these metabolites was achieved through comprehensive spectroscopic analyses and single-crystal X-ray diffraction, confirming their identities as oxygenated sesquiterpene derivatives. Future investigations should extend these findings through in vivo and ex vivo studies, particularly by examining the interaction of carotol and its metabolites with human gut microbiota, to better understand their metabolic transformations and potential health implications. Cytotoxicity assays revealed that carotol exhibited the strongest cytotoxic activity against multiple human carcinoma cell lines, while its metabolites showed moderate yet selective cytotoxicity toward cancer cells compared to normal cells. This selective activity may, in part, be associated with potential interactions with NADPH oxidase, as previously reported for other sesquiterpene alcohols; however, further biochemical validation is required to confirm this mechanism. Molecular docking and dynamics simulations supported these findings, indicating that carotol and CM2 bind NADPH oxidase with higher affinity and stability, potentially underpinning their cytotoxic mechanisms through enzyme inhibition. This work provides novel insights into the microbial metabolism of carotol, a bioactive sesquiterpene with promising cytotoxic properties, and highlights the utility of microbial biotransformation as a tool for generating and characterizing novel derivatives. Future studies are warranted to further explore the pharmacokinetics, in vivo efficacy, and safety profiles of carotol metabolites, ultimately contributing to the development of new therapeutic agents derived from natural products.

## Figures and Tables

**Table 1:** ^1^H NMR spectroscopic data (600 MHz, CDCl_3_) of carotol and its metabolites.

	ẟ_H,_ Multiplicity (*J* in Hz)			
	Carotol	CM1	CM2	CM3
2	1.73, dd (12.1, 8.4) 2.29, d (16.2)	1.22, dd (12.0, 12.0) 1.75, dd (12.9, 6.0)	1.79, dd (12.2, 5.6) 2.27, m	1.39, m 1.55, dd (12.0, 10.2)
3	5.35, d	3.76, m	3.85, m	3.62, d (10.2)
5	2.10, dd (5.4, 5.4) -	1.34, dd (5.4, 1.2) 1.85, m	1.35, m 1.92, m	1.46, m 1.88, m
6	1.65, m 1.97, m	1.54, ddd (13.0, 13.0, 4.8) 2.14, m	1.25, m 1.78, m	1.68, m 1.74, m
8	1.82, m	1.85, m	2.45, d (8.0)	1.47, m
9	1.52, m 1.67, m	4.47, ddd (7.2, 7.2, 0.6)	4.56, m	1.45, m 1.63, m
10	1.32, m 1.58, m	1.45, dd (13.2, 0.6) 1.80, ddd (13.8, 7.2, 0.6)	1.60, m 1.65, m	1.47, m 1.93, m
11	0.96, s	1.36, s	1.38, s	1.01, s
12	1.68, s	1.30, s	1.37, s	1.24, s
13	1.83, m	2.10, m	-	1.64, m
14	1.02, d (6.6)	1.04, d (6.0)	1.47, s	0.98, d (6.6)
15	0.97, d (6.0)	1.17, d (6.6)	1.48, s	0.90, d (6.6)

**Table 2:** ^13^C NMR spectroscopic data (150 MHz, CDCl_3_) of carotol and its metabolites.

	ẟc, Multiplicity ^a^			
	Carotol	CM1	CM2	CM3
1	49.3, s	44.8, s	45.5, s	47.5, s
2	38.8, t	40.8, t	43.4, t	38.7, t
3	122.3, d	71.5, d	71.1, d	75.4, d
4	138.8, s	85.6, s	87.4, s	75.5, s
5	29.6, t	29.2, t	29.1, t	35.5, t
6	34.6, t	41.7, t	40.8, t	41.3, t
7	84.8, s	92.6, s	91.9, s	83.6, s
8	52.7, d	58.8, d	60.1, d	56.6, d
9	24.6, t	73.3, d	74.3, d	26.5, t
10	39.6, t	45.4, t	43.7, t	34.1, t
11	21.7, q	23.1, q	23.0, q	25.9, q
12	25.2, q	24.1, q	24.3, q	24.4, q
13	27.8, d	29.4, d	74.1, s	28.9, d
14	24.3, q	26.0, q	31.1, q	23.5, q
15	21.6, q	22.7, q	30.2, q	21.8, q

^a^ Carbon multiplicities were determined by DEPT 135°.

**Table 3:** Summary of the various crystallographic parameters of the compound CM1.

Parameter	Value
Chemical formula	C_16_H_30_O_4_
*M*_r_	286.40
Crystal system, space group	Orthorhombic, *P*2_1_2_1_2_1_
Temperature (K)	150
*a*, *b*, *c* (Å)	6.4762 (3), 15.7204 (9), 15.9679 (9)
*α*, *β*, *γ* (°)	90, 90, 90
*V* (Å^3^)	1625.67 (15)
*Z*	4
Radiation type	Cu *K*α
*µ* (mm^−1^)	0.66
Crystal size (mm)	0.21 × 0.19 × 0.15
Diffractometer	Bruker *APEX*-II CCD
Absorption correction	Multi-scan: SADABS2016/2—Bruker AXS area detector scaling and absorption correction
*T_min_*, *T_max_*	0.86, 0.91
No. of measured, independent & observed [*I* > 2*σ*(*I*)] reflections	10,554, 2864, 2713
*R_int_*	0.037
(sin θ/λ)_max_ (Å^−1^)	0.596
*R*[*F*^2^ > 2*σ*(*F*^2^)], *wR*(*F*^2^), *S*	0.037, 0.099, 1.08
No. of reflections	2864
No. of parameters	189
H-atom treatment	Constrained
Δ*ρ_max_*, Δ*ρ_min_* (*e* Å^−3^)	0.13−0.20

**Table 4:** Cytotoxic activity (IC_50_ ± SD, µM) and selectivity indices (SI) of carotol and its metabolites against selected cancer and normal cell lines.

Cell Line ^a^	Carotol	CM1	CM2	CM3	*Cis*-Platin	Doxorubicin
HepG-2	52.34 ± 4.23 ^b^ SI = 3.36	220.74 ± 12.26 ^a^ SI = 2.11	154.45 ± 9.39 ^a^ SI = 1.60	195.53 ± 10.57 ^a^ SI = 1.62	11.96 ± 1.23 ^c^ SI = 13.12	1.27 ± 0.20 ^d^ SI = 34.62
HCT-116	25.68 ± 0.53 ^b^ SI = 6.85	339.38 ± 15.56 ^a^ SI = 1.37	180.64 ± 9.95 ^a^ SI = 1.37	226.38 ± 11.04 ^a^ SI = 1.40	17.96 ± 1.76 ^b^ SI = 8.74	2.99 ± 0.22 ^c^ SI = 14.66
MCF-7	68.38 ± 6.04 ^b^ SI = 2.57	400.75 ± 20.28 ^a^ SI = 1.16	212.85 ± 13.72 ^a^ SI = 1.16	242.14 ± 15.68 ^a^ SI = 1.31	19.03 ± 2.10 ^c^ SI = 8.25	3.07 ± 0.18 ^d^ SI = 14.31
A-549	28.65 ± 2.75 ^b^ SI = 6.14	225.38 ± 13.40 ^a^ SI = 2.07	138.21 ± 9.20 ^a^ SI = 1.79	205.24 ± 10.61 ^a^ SI = 1.54	24.53 ± 2.26 ^b^ SI = 6.40	3.31 ± 0.26 ^c^ SI = 12.71
MRC-5	175.61 ± 10.93 ^ab^	466.17 ± 20.32 ^a^	247.62 ± 11.24 ^ab^	316.36 ± 15.17 ^a^	156.46 ± 6.48 ^ab^	43.96 ± 3.24 ^b^

^a^ HepG-2: hepatocellular carcinoma; HCT-116: colon carcinoma; MCF-7: breast carcinoma; A-549: lung carcinoma; MRC-5: normal human lung fibroblasts. Data represent mean ± SD of three independent experiments (each performed in triplicate). Statistical analysis was carried out using one-way ANOVA followed by Tukey’s post hoc test. Groups sharing the same superscript letter are not significantly different (*p* > 0.05); different letters indicate significant differences vs. carotol: (^a^ vs. ^b^) *p* < 0.05; (^a^ vs. ^c^) *p* < 0.01; (^a^ vs. ^d^) *p* < 0.001. Selectivity Index (SI) = CC_50_(MRC-5)/IC_50_(cancer cell).

**Table 5:** Docking results against NADPH oxidase (PBD = 8gz3).

Compound	Free Energy of Binding (Kcal/mol)	RMSD (Å)	Interacted Residues
Carotol	−5.65	1.35	His338, Trp361
CM1	−5.11	1.91	Trp361
CM2	−5.41	1.50	Gly359, His338
CM3	−5.16	1.85	Thr362

## Data Availability

All data generated or analyzed during this study are included in this published article and its Supplementary Materials.

## Supplementary Materials

The following supporting information can be downloaded at: https://www.mdpi.com/article/10.3390/biom15121651/s1. All 1D and 2D NMR spectra of carotol and its metabolites (Figure S1), TLC plate of carotol metabolites (Figure S2), dose–response curve of cis-platin (Figure S3), and 95% Confidence Intervals (Table S1) accompany this paper.

## Abbreviations

The following abbreviations are used in this manuscript:

API	Active Pharmaceutical Ingredient
ATCC	American Type Culture Collection
AUMC	Assiut University Mycology Center
BChE	Butyrylcholinesterase
CM1,2,3	Carotol metabolites 1, 2, and 3
COX-2	Cyclooxygenase-2
COSY	Correlation Spectroscopy
DEPT	Distortionless Enhancement by Polarization Transfer
DMF	*N,N*-Dimethylformamide
EIHRMS	Electron Impact High-Resolution Mass Spectrometry
HPLC	High-Performance Liquid Chromatography
HSQC	Heteronuclear Single Quantum Coherence
HMBC	Heteronuclear Multiple Bond Correlation
IC_50_	Half Maximal Inhibitory Concentration
IR	Infrared Spectroscopy
MD	Molecular Dynamic
MTT	3-(4,5-Dimethylthiazol-2-yl)-2,5-Diphenyltetrazolium Bromide
NRRL	Northern Regional Research Laboratories
PBS	Phosphate-Buffered Saline
PTFE	Polytetrafluoroethylene
RMSD	Root Mean Square Deviation
RMSF	Root Mean Square Fluctuations
TLC	Thin-Layer Chromatography
TMS	Tetramethylsilane
UV	Ultraviolet
XRD	X-Ray Diffraction
