# Supplementary material for: Microbial Biotransformation of the Sesquiterpene Carotol: Generation of Hydroxylated Metabolites with Potential Cytotoxic and Target-Specific Binding Activities
Source: Biomolecules. 2025 Nov 26;15(12):1651. doi: 10.3390/biom15121651 (PMC12731232; doi:10.3390/biom15121651)
Supplement: Supplementary file 1 [file biomolecules-15-01651-s001.zip › biomolecules-3927078-supplementary.pdf]

Microbial Biotransformation of the Sesquiterpene Carotol: Generation of Hydroxylated Metabolites with Potential Cytotoxic and Target-Specific Binding Activities

Hanan G. Sary [1,2], Mohammed A. Khedr [1,3], Mohamed M. Radwan [4,5], Mickey Vinodh [6], and Khaled Y. Orabi [1*]

[1] Department of Pharmaceutical Chemistry, College of Pharmacy, Kuwait University, Safat 13110, Kuwait; hanan.sary@ku.edu.kw, mohammed.khedr@ku.edu.kw, ky.orabi@ku.edu.kw
[2] Department of Pharmacognosy, Faculty of Pharmacy, Ain-Shams University, Cairo, Egypt; hanangaber@pharma.asu.edu.eg
[3] Department of Pharmaceutical Chemistry, Faculty of Pharmacy, Helwan University, Ain Helwan, Cairo 11795, Egypt
[4] Department of Biomolecular Sciences, School of Pharmacy, The University of Mississippi, University, MS 38677, USA; mradwan@olemiss.edu
[5] National Center for Natural Products Research, School of Pharmacy, The University of Mississippi, University, MS 38677, USA; mradwan@olemiss.edu
[6] Research Sector Project Units, Faculty of Science, Kuwait University, Safat 13060, Kuwait; miky.findo@ku.edu.kw
* Correspondence: ky.orabi@ku.edu.kw; Tel.: +965-2463-6048

Table S1. IC_{50} values (mean ± 95% CI) of carotol, its metabolites, and *cis*-platin.

Cell line[a]	IC_{50} (μM) ± CI				
	Carotol	CM1	CM2	CM3	*Cis*-platin
HepG-2	52.34 ± 10.51	220.74 ± 30.43	154.45 ± 23.33	195.53 ± 26.23	11.96 ± 3.06
HCT-116	25.68 ± 1.32	339.38 ± 38.65	180.64 ± 24.72	226.38 ± 27.38	17.96 ± 4.37
MCF-7	68.38 ± 15.00	400.75 ± 50.38	212.85 ± 34.05	242.14 ± 38.96	19.03 ± 5.21
A-549	28.65 ± 6.83	225.38 ± 33.32	138.21 ± 22.85	205.24 ± 26.36	24.53 ± 5.61
MRC-5	175.61 ± 27.17	466.17 ± 50.49	247.62 ± 27.93	316.36 ± 37.70	N/A

[a] *HepG-2: hepatocellular carcinoma, HCT-116: colon carcinoma, MCF-7: breast carcinoma, A-549: lung carcinoma, MRC-5: normal human lung fibroblasts.*

Microbial Biotransformation of the Sesquiterpene Carotol: Generation of Hydroxylated Metabolites with Potential Cytotoxic and Target-Specific Binding Activities

Hanan G. Sary [1,2], Mohammed A. Khedr [1,3], Mohamed M. Radwan [4,5], Mickey Vinodh [6], and Khaled Y. Orabi [1*]

[1] Department of Pharmaceutical Chemistry, College of Pharmacy, Kuwait University, Safat 13110, Kuwait; hanan.sary@ku.edu.kw, mohammed.khedr@ku.edu.kw, ky.orabi@ku.edu.kw

[2] Department of Pharmacognosy, Faculty of Pharmacy, Ain-Shams University, Cairo, Egypt; hanangaber@pharma.asu.edu.eg

[3] Department of Pharmaceutical Chemistry, Faculty of Pharmacy, Helwan University, Ain Helwan, Cairo 11795, Egypt

[4] Department of Biomolecular Sciences, School of Pharmacy, The University of Mississippi, University, MS 38677, USA; mradwan@olemiss.edu

[5] National Center for Natural Products Research, School of Pharmacy, The University of Mississippi, University, MS 38677, USA; mradwan@olemiss.edu

[6] Research Sector Project Units, Faculty of Science, Kuwait University, Safat 13060, Kuwait; miky.findo@ku.edu.kw

* Correspondence: ky.orabi@ku.edu.kw; Tel.: +965-2463-6048

Figure S1

Carotol

1H spectra Dr.Orabi Caro-2 in CDCL3

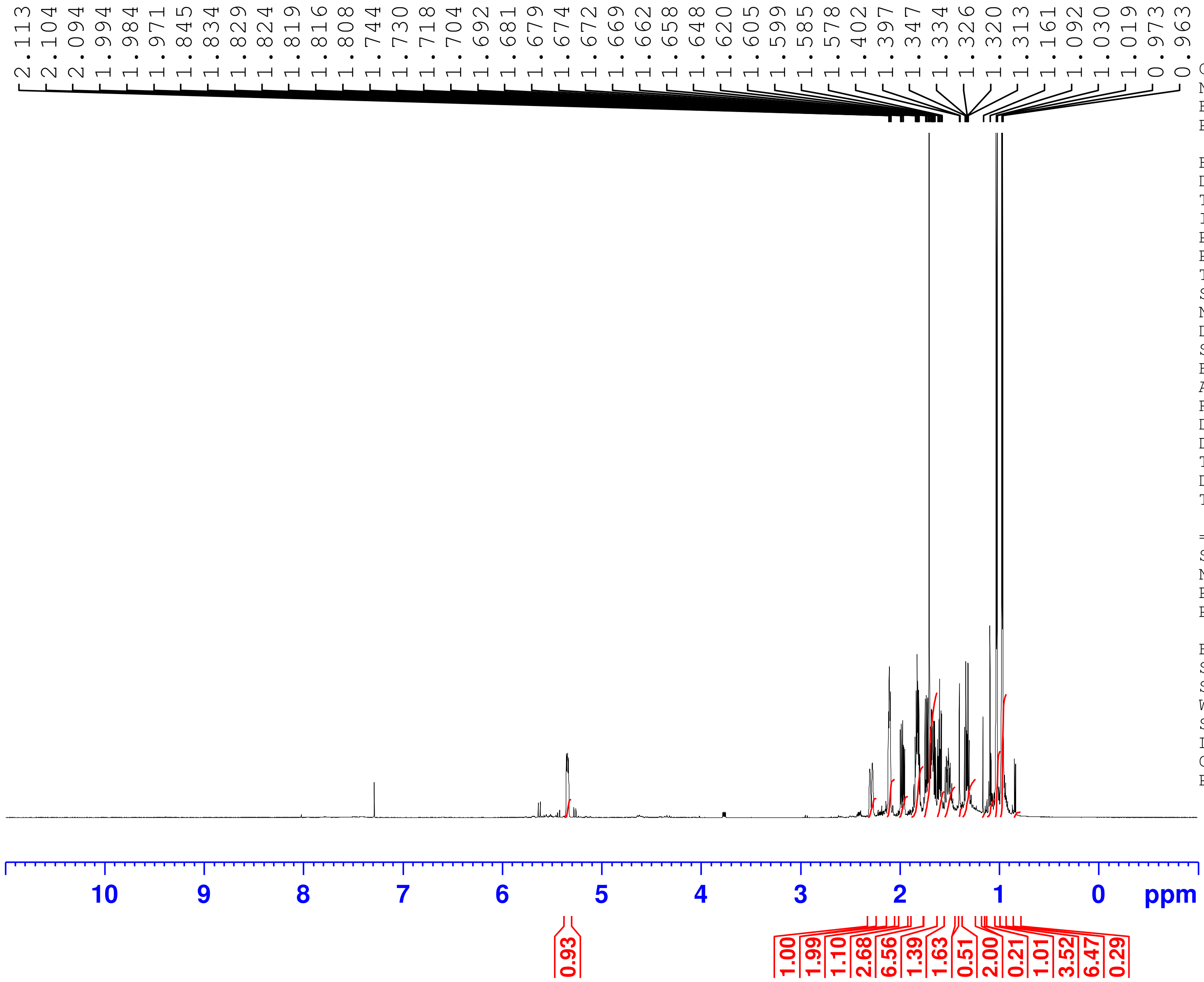


```
Current Data Parameters
NAME            Caro-2-2D
EXPNO                   1
PROCNO                  1

F2 - Acquisition Parameters
Date_            20211020
Time                10.42
INSTRUM             spect
PROBHD      5 mm PABBO BB-
PULPROG              zg30
TD                  65536
SOLVENT             CDCl3
NS                     16
DS                      2
SWH             12335.526 Hz
FIDRES           0.188225 Hz
AQ              2.6563926 sec
RG                    161
DW                 40.533 usec
DE                   6.50 usec
TE                  298.0 K
D1             1.00000000 sec
TD0                     1

======== CHANNEL f1 ========
SFO1          600.1337060 MHz
NUC1                   1H
P1                  10.60 usec
PLW1          27.82500076 W

F2 - Processing parameters
SI                  32768
SF            600.1300000 MHz
WDW                    EM
SSB      0
LB                   0.30 Hz
GB       0
PC                   1.00
```

Carotol

1H spectra Dr.Orabi Caro-2 in CDCL3

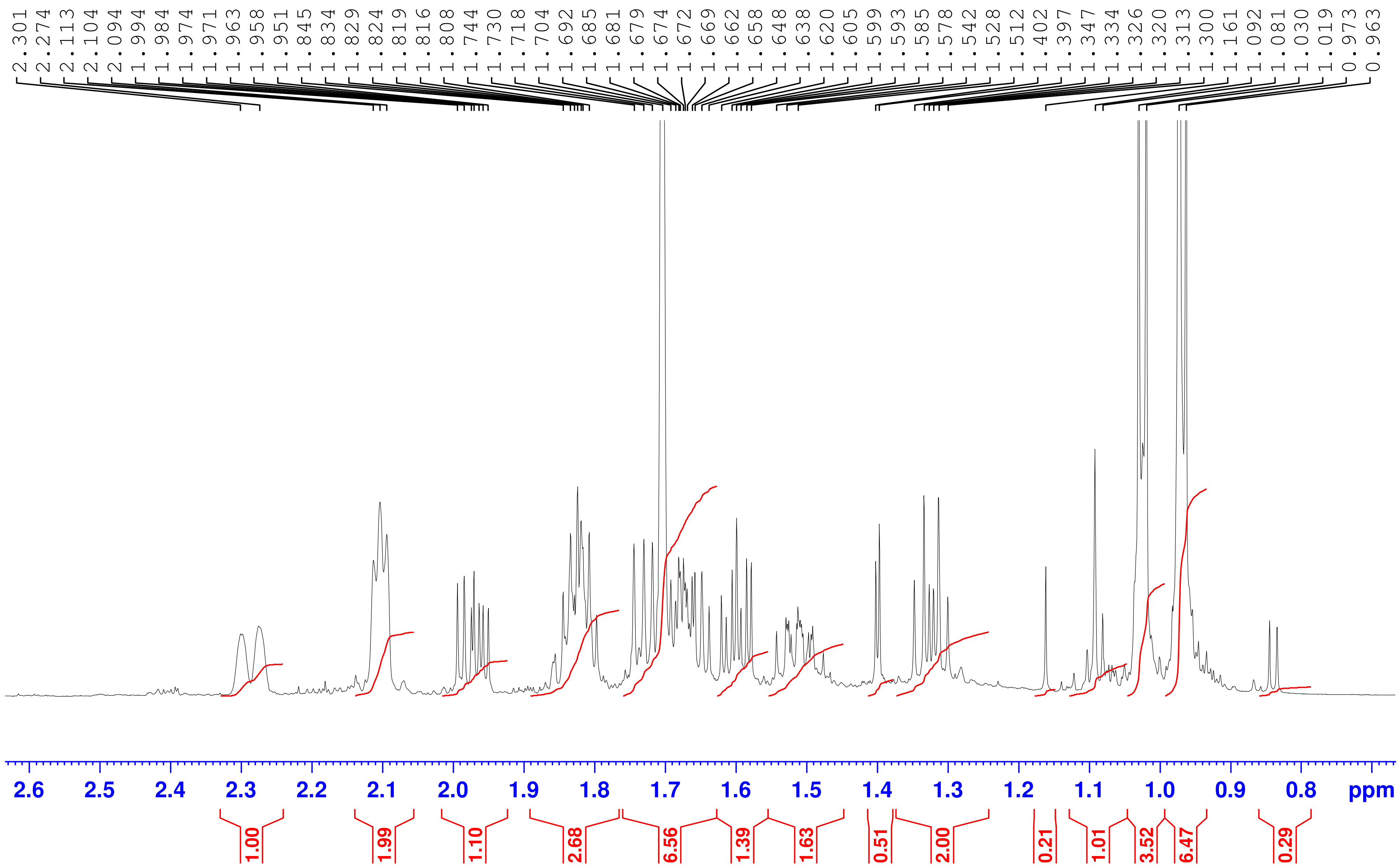

Carotol

13C decoupled spectra Dr.Orabi Caro-2 in CDCL3

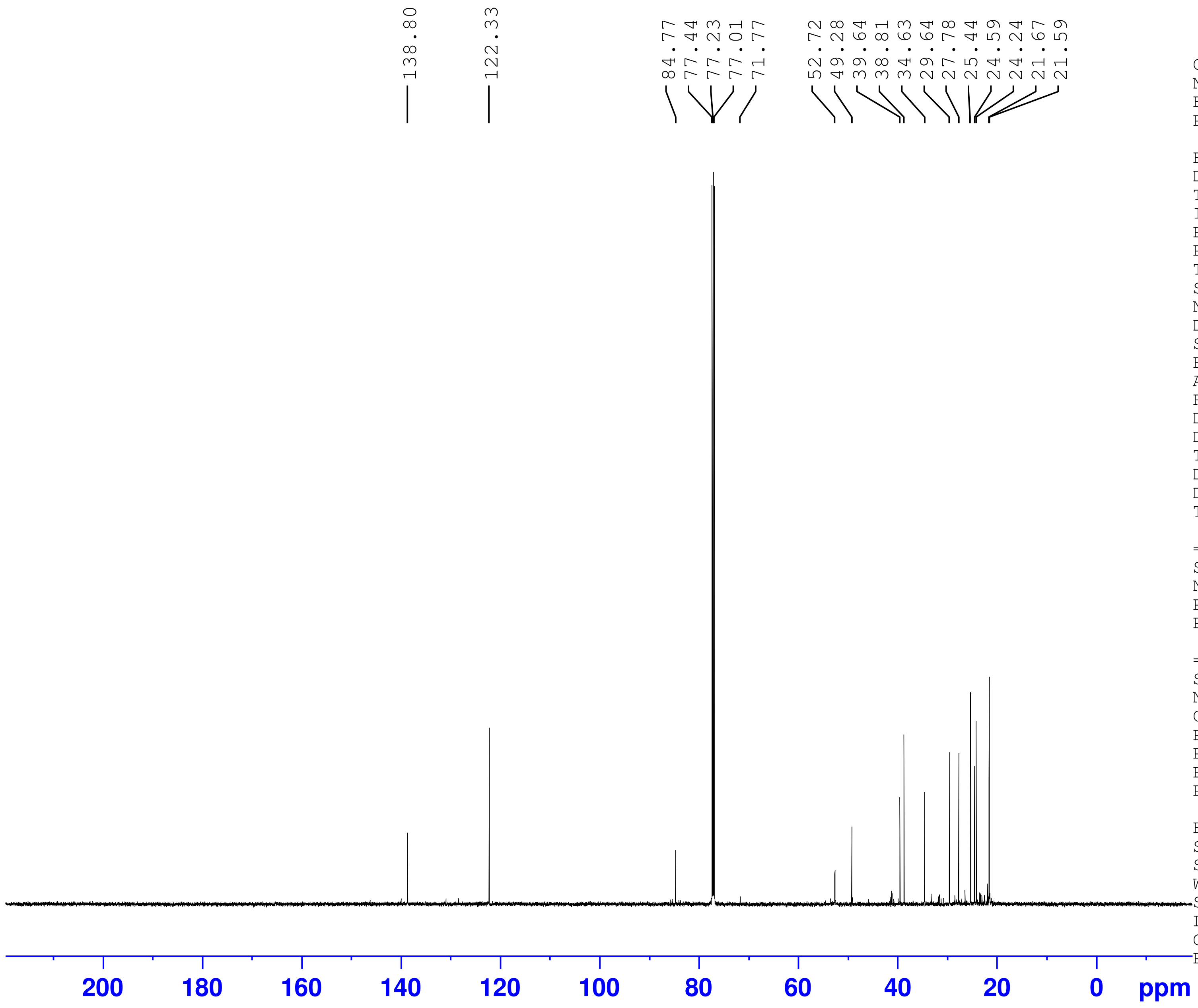


```
Current Data Parameters
NAME             Caro-2-2D
EXPNO                    2
PROCNO                   1

F2 - Acquisition Parameters
Date_             20211021
Time                  4.59
INSTRUM              spect
PROBHD     5 mm PABBO BB-
PULPROG            zgpg30
TD                  65536
SOLVENT             CDCl3
NS                   2048
DS                      4
SWH             36057.691 Hz
FIDRES           0.550197 Hz
AQ              0.9087659 sec
RG                    203
DW                 13.867 usec
DE                  50.00 usec
TE                  299.0 K
D1             2.00000000 sec
D11            0.03000000 sec
TD0                     1

======== CHANNEL f1 ========
SFO1          150.9178979 MHz
NUC1                  13C
P1                   8.80 usec
PLW1          78.13500214 W

======== CHANNEL f2 ========
SFO2          600.1324005 MHz
NUC2                   1H
CPDPRG[2          waltz65
PCPD2               70.00 usec
PLW2          27.82500076 W
PLW12          0.63804001 W
PLW13          0.32093000 W

F2 - Processing parameters
SI                  32768
SF            150.9027774 MHz
WDW                    EM
SSB       0
LB                   1.00 Hz
GB        0
PC                   1.40
```

Carotol

13C decoupled spectra Dr.Orabi Caro-2 in CDCL3

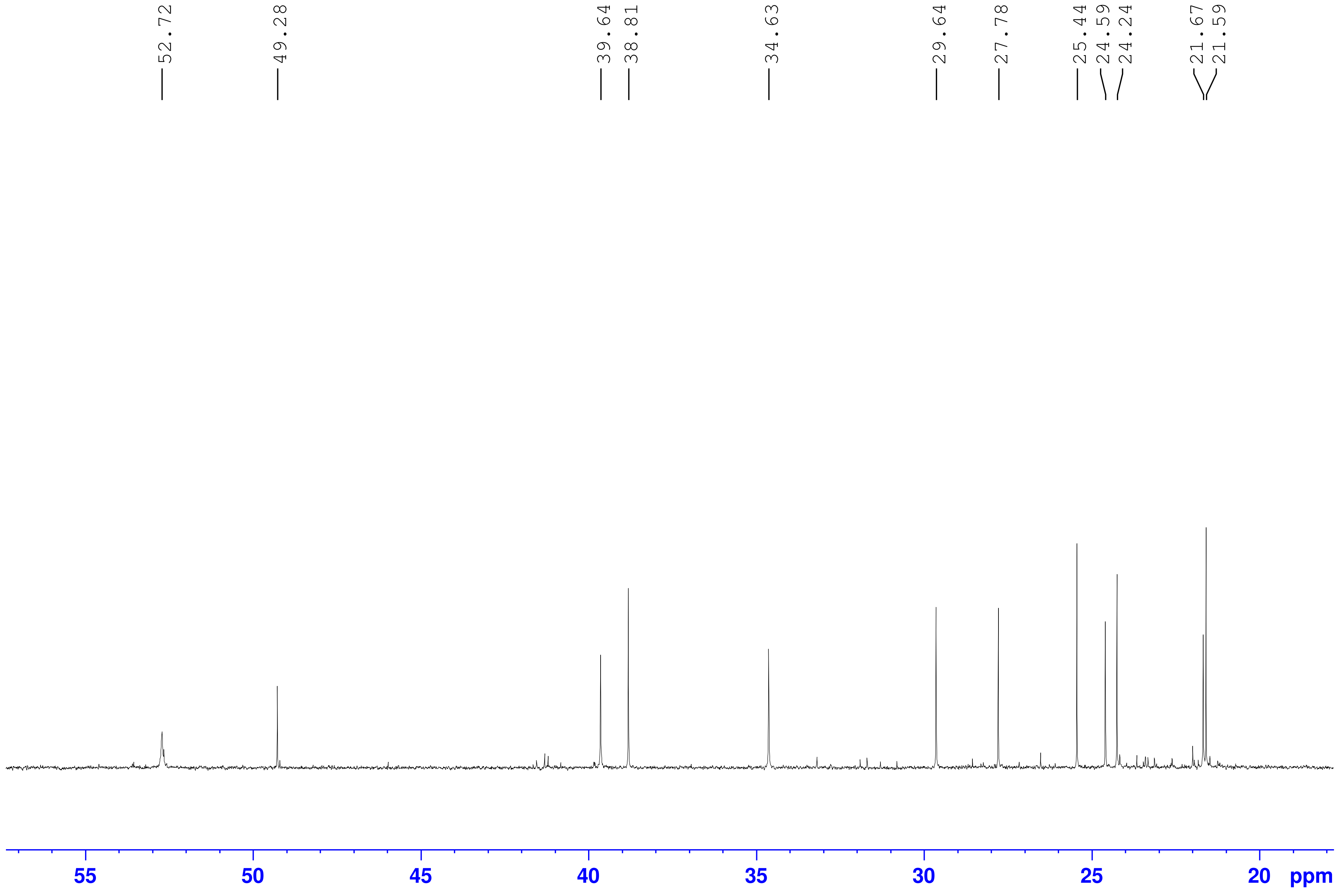

Carotol

DEPT 135 spectra Dr.Orabi Caro-1 in CDCL3

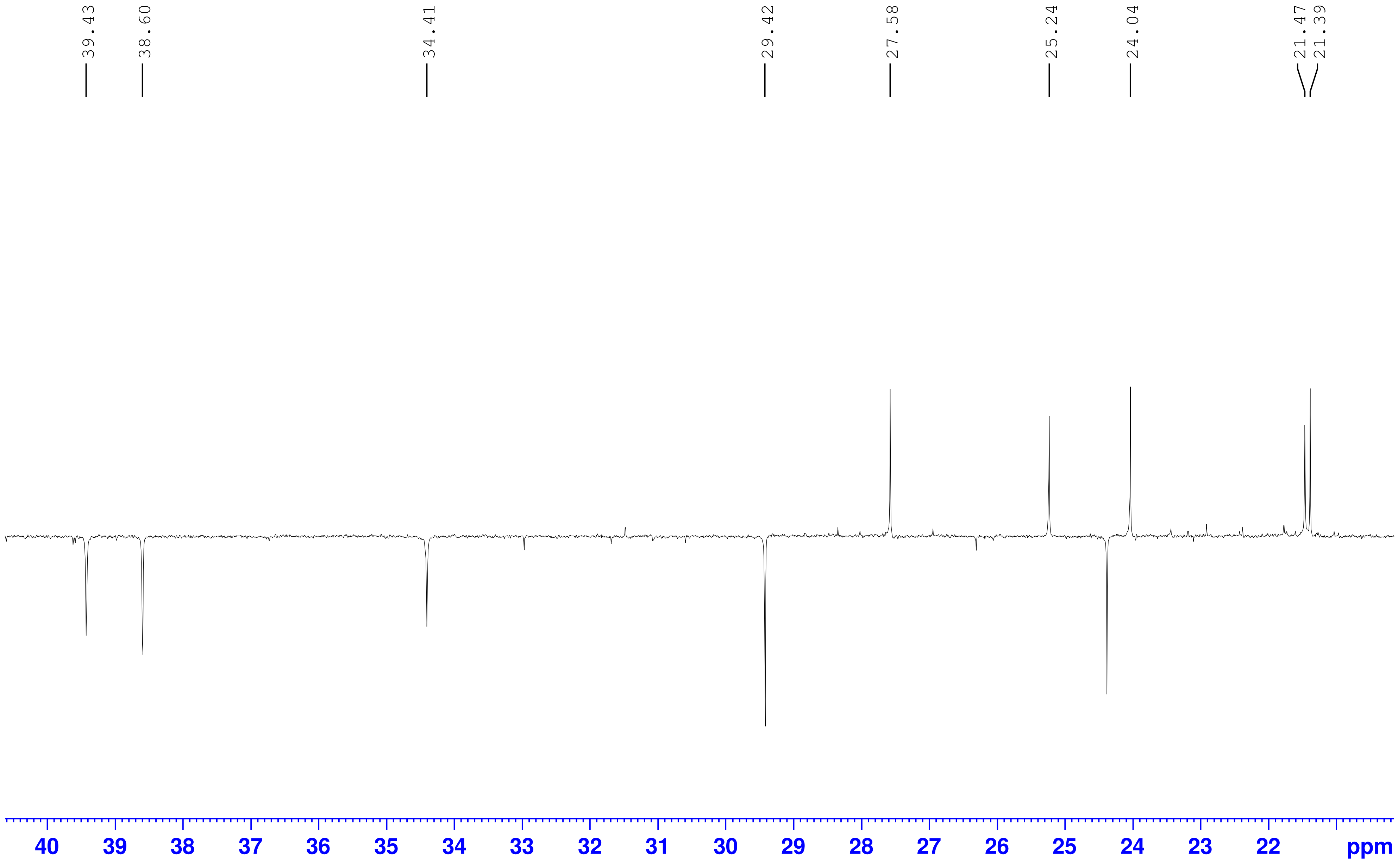

Carotol

13C HSQC spectra Dr.Orabi Caro-2 in CDCL3

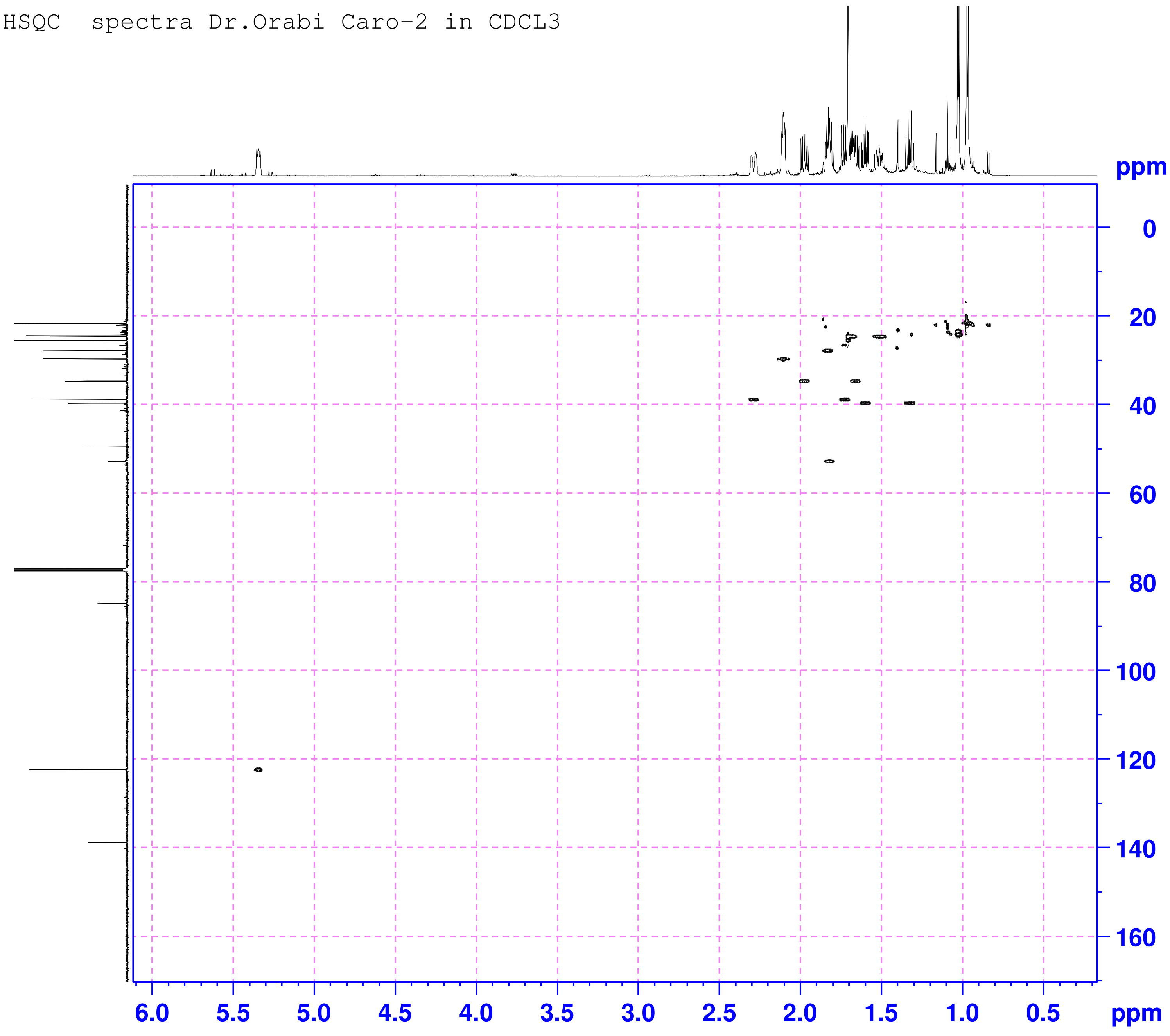

Current Data Parameters

NAME	Caro-2-2D
EXPNO	4
PROCNO	1

F2 - Acquisition Parameters

Date_	20211021
Time	5.26
INSTRUM	spect
PROBHD	5 mm PABBO BB-
PULPROG	hsqcetgpsp.2
TD	2048
SOLVENT	CDCl3
NS	8
DS	16
SWH	3571.428 Hz
FIDRES	1.743862 Hz
AQ	0.2867200 sec
RG	203
DW	140.000 usec
DE	6.50 usec
TE	298.5 K
CNST2	145.0000000
D0	0.00000300 sec
D1	1.81977606 sec
D4	0.00172414 sec
D11	0.03000000 sec
D16	0.00020000 sec
IN0	0.00001840 sec
ZGOPTNS	

======== CHANNEL f1 ========

SFO1	600.1318862 MHz
NUC1	1H
P1	10.60 usec
P2	21.20 usec
P28	1000.00 usec
PLW1	27.82500076 W

======== CHANNEL f2 ========

SFO2	150.9148803 MHz
NUC2	13C
CPDPRG[2	garp4
P3	8.80 usec
P14	500.00 usec
P24	2000.00 usec
PCPD2	60.00 usec
PLW0	0 W
PLW2	78.13500214 W
PLW12	1.68079996 W
SPNAM[3]	Crp60,0.5,20.1
SPOAL3	0.500
SPOFFS3	0 Hz
SPW3	9.24489975 W
SPNAM[7]	Crp60comp.4
SPOAL7	0.500
SPOFFS7	0 Hz
SPW7	9.24489975 W

====== GRADIENT CHANNEL =====

GPNAM[1]	SINE.100
GPNAM[2]	SINE.100
GPZ1	80.00 %
GPZ2	20.10 %
P16	1000.00 usec

F1 - Acquisition parameters

TD	512
SFO1	150.9149 MHz
FIDRES	53.074047 Hz
SW	180.061 ppm
FnMODE	Echo-Antiecho

F2 - Processing parameters

SI	2048
SF	600.1300000 MHz
WDW	QSINE
SSB	2
LB	0 Hz
GB	0
PC	1.40

F1 - Processing parameters

SI	1024
MC2	echo-antiecho
SF	150.9027685 MHz
WDW	QSINE
SSB	2
LB	0 Hz
GB	0

Carotol

13C HSQC spectra Dr.Orabi Caro-2 in CDCL3

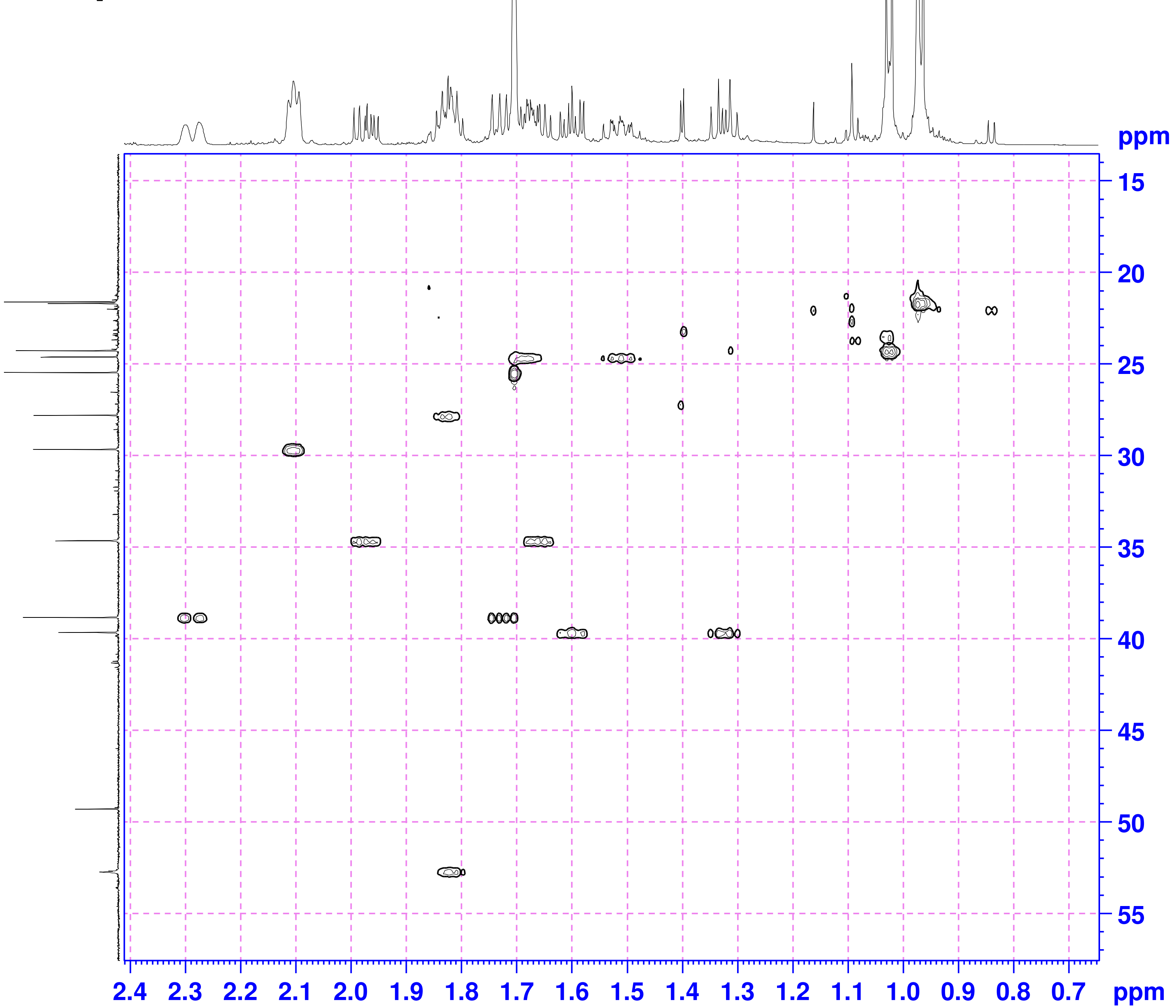


```
Current Data Parameters
NAME            Caro-2-2D
EXPNO                   4
PROCNO                  1

F2 - Acquisition Parameters
Date_            20211021
Time                 5.26
INSTRUM             spect
PROBHD    5 mm PABBO BB-
PULPROG    hsqcetgpsp.2
TD                   2048
SOLVENT             CDCl3
NS                      8
DS                     16
SWH              3571.428 Hz
FIDRES           1.743862 Hz
AQ              0.2867200 sec
RG                    203
DW                140.000 usec
DE                   6.50 usec
TE                  298.5 K
CNST2         145.0000000
D0             0.00000300 sec
D1             1.81977606 sec
D4             0.00172414 sec
D11            0.03000000 sec
D16            0.00020000 sec
IN0            0.00001840 sec
ZGOPTNS

======== CHANNEL f1 ========
SFO1          600.1318862 MHz
NUC1                   1H
P1                  10.60 usec
P2                  21.20 usec
P28               1000.00 usec
PLW1          27.82500076 W

======== CHANNEL f2 ========
SFO2          150.9148803 MHz
NUC2                  13C
CPDPRG[2            garp4
P3                   8.80 usec
P14                500.00 usec
P24               2000.00 usec
PCPD2               60.00 usec
PLW0        0 W
PLW2          78.13500214 W
PLW12          1.68079996 W
SPNAM[3] Crp60,0.5,20.1
SPOAL3              0.500
SPOFFS3     0 Hz
SPW3           9.24489975 W
SPNAM[7]     Crp60comp.4
SPOAL7              0.500
SPOFFS7     0 Hz
SPW7           9.24489975 W

====== GRADIENT CHANNEL =====
GPNAM[1]         SINE.100
GPNAM[2]         SINE.100
GPZ1                80.00 %
GPZ2                20.10 %
P16               1000.00 usec

F1 - Acquisition parameters
TD                    512
SFO1             150.9149 MHz
FIDRES          53.074047 Hz
SW                180.061 ppm
FnMODE       Echo-Antiecho

F2 - Processing parameters
SI                   2048
SF            600.1300000 MHz
WDW                 QSINE
SSB                     2
LB          0 Hz
GB          0
PC                   1.40

F1 - Processing parameters
SI                   1024
MC2          echo-antiecho
SF            150.9027685 MHz
WDW                 QSINE
SSB                     2
LB          0 Hz
GB          0
```

Carotol

13C HMBC spectra Dr.Orabi Caro-1 in CDCL3

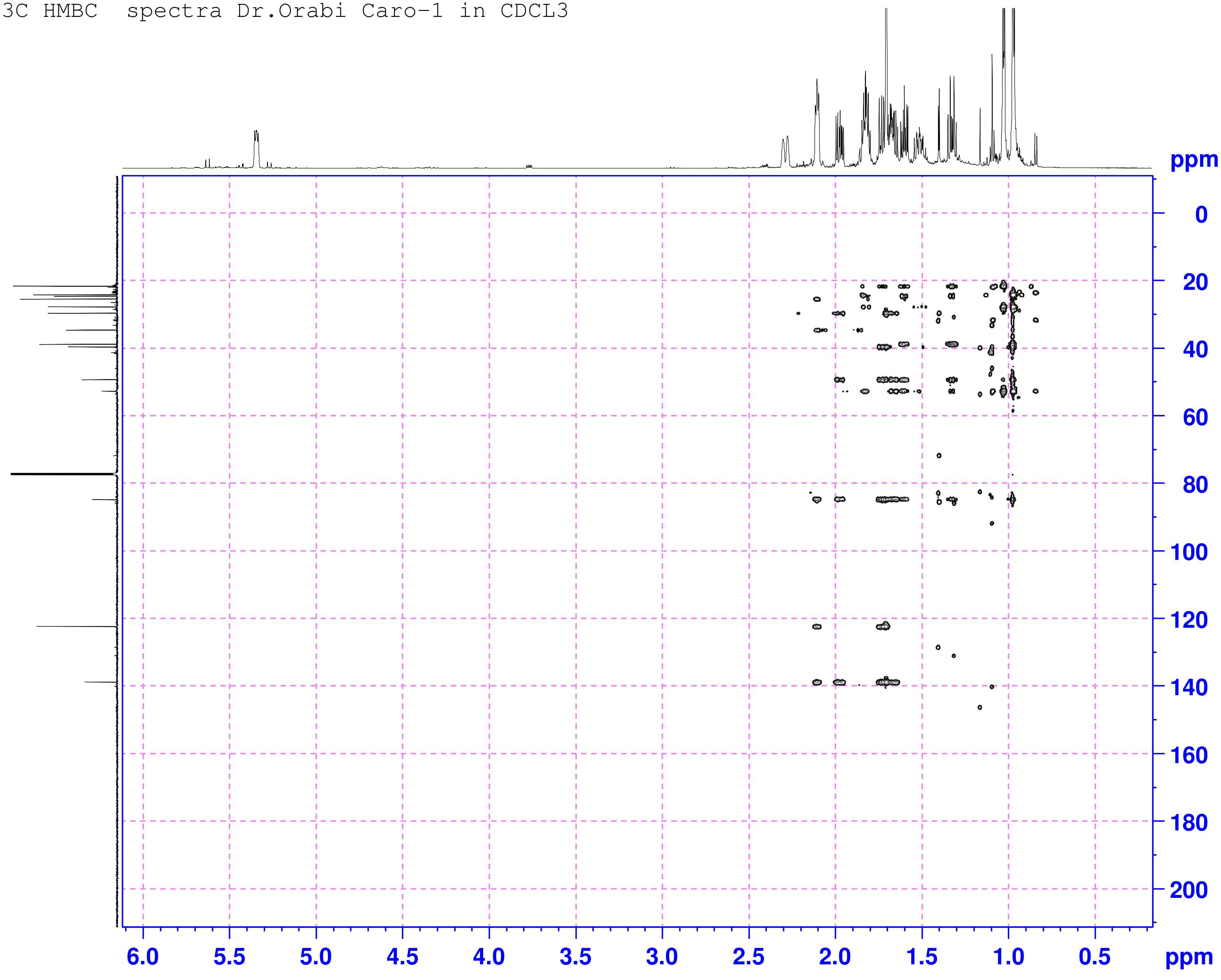


```
Current Data Parameters
NAME           Caro-2-2D
EXPNO                  5
PROCNO                 1

F2 - Acquisition Parameters
Date_           20211021
Time                7.55
INSTRUM            spect
PROBHD    5 mm PABBO BB-
PULPROG     hmbcetgpl2nd
TD                  2048
SOLVENT            CDCl3
NS                    32
DS                    16
SWH             3571.428 Hz
FIDRES          1.743862 Hz
AQ             0.2867200 sec
RG                   203
DW               140.000 usec
DE                  6.50 usec
TE                 297.9 K
CNST6        125.0000000
CNST7        165.0000000
CNST13        10.0000000
CNST30         0.5981148
D0            0.00000300 sec
D1            1.34435201 sec
D6            0.05000000 sec
D16           0.00020000 sec
IN0           0.00001490 sec

======== CHANNEL f1 ========
SFO1        600.1318862 MHz
NUC1                  1H
P1                 10.60 usec
P2                 21.20 usec
PLW1         27.82500076 W

======== CHANNEL f2 ========
SFO2        150.9178738 MHz
NUC2                 13C
P3                  8.80 usec
P24              2000.00 usec
PLW2         78.13500214 W
SPNAM[7]     Crp60comp.4
SPOAL7             0.500
SPOFFS7    0 Hz
SPW7          9.24489975 W

====== GRADIENT CHANNEL =====
GPNAM[1]       SINE.100
GPNAM[3]       SINE.100
GPNAM[4]       SINE.100
GPNAM[5]       SINE.100
GPZ1              80.00 %
GPZ3              15.00 %
GPZ4             -10.00 %
GPZ5              -5.00 %
P16             1000.00 usec

F1 - Acquisition parameters
TD                   256
SFO1            150.9179 MHz
FIDRES        131.082214 Hz
SW               222.353 ppm
FnMODE      Echo-Antiecho

F2 - Processing parameters
SI                  2048
SF           600.1300000 MHz
WDW                 SINE
SSB        0
LB         0 Hz
GB         0
PC                  1.40

F1 - Processing parameters
SI                   512
MC2          echo-antiecho
SF           150.9027541 MHz
WDW                 SINE
SSB                    2
LB         0 Hz
GB         0
```

Carotol

13C HMBC spectra Dr.Orabi Caro-1 in CDCL3

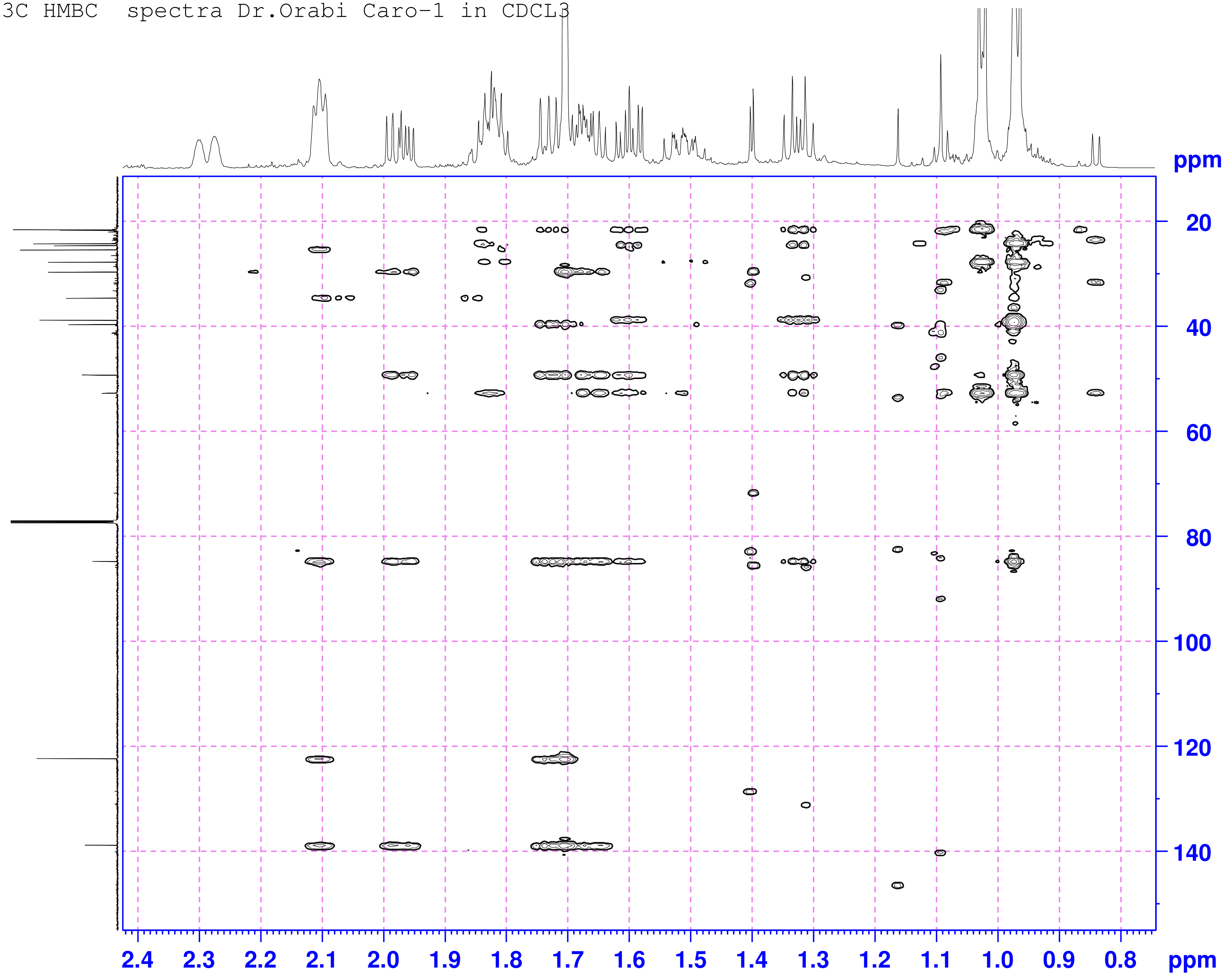


```
Current Data Parameters
NAME              Caro-2-2D
EXPNO                     5
PROCNO                    1

F2 - Acquisition Parameters
Date_              20211021
Time                   7.55
INSTRUM               spect
PROBHD   5 mm PABBO BB-
PULPROG     hmbcetgpl2nd
TD                     2048
SOLVENT               CDCl3
NS                       32
DS                       16
SWH                3571.428 Hz
FIDRES             1.743862 Hz
AQ                0.2867200 sec
RG                      203
DW                  140.000 usec
DE                     6.50 usec
TE                    297.9 K
CNST6           125.0000000
CNST7           165.0000000
CNST13           10.0000000
CNST30            0.5981148
D0               0.00000300 sec
D1               1.34435201 sec
D6               0.05000000 sec
D16              0.00020000 sec
IN0              0.00001490 sec

======== CHANNEL f1 ========
SFO1            600.1318862 MHz
NUC1                     1H
P1                    10.60 usec
P2                    21.20 usec
PLW1            27.82500076 W

======== CHANNEL f2 ========
SFO2            150.9178738 MHz
NUC2                    13C
P3                     8.80 usec
P24                 2000.00 usec
PLW2            78.13500214 W
SPNAM[7]        Crp60comp.4
SPOAL7                0.500
SPOFFS7   0 Hz
SPW7             9.24489975 W

====== GRADIENT CHANNEL =====
GPNAM[1]           SINE.100
GPNAM[3]           SINE.100
GPNAM[4]           SINE.100
GPNAM[5]           SINE.100
GPZ1                  80.00 %
GPZ3                  15.00 %
GPZ4                 -10.00 %
GPZ5                  -5.00 %
P16                 1000.00 usec

F1 - Acquisition parameters
TD                      256
SFO1               150.9179 MHz
FIDRES           131.082214 Hz
SW                  222.353 ppm
FnMODE         Echo-Antiecho

F2 - Processing parameters
SI                     2048
SF              600.1300000 MHz
WDW                    SINE
SSB       0
LB        0 Hz
GB        0
PC                     1.40

F1 - Processing parameters
SI                      512
MC2            echo-antiecho
SF              150.9027541 MHz
WDW                    SINE
SSB                       2
LB        0 Hz
GB        0
```

COSY   spectra Dr.Orabi Caro-2 in CDCL3

Carotol

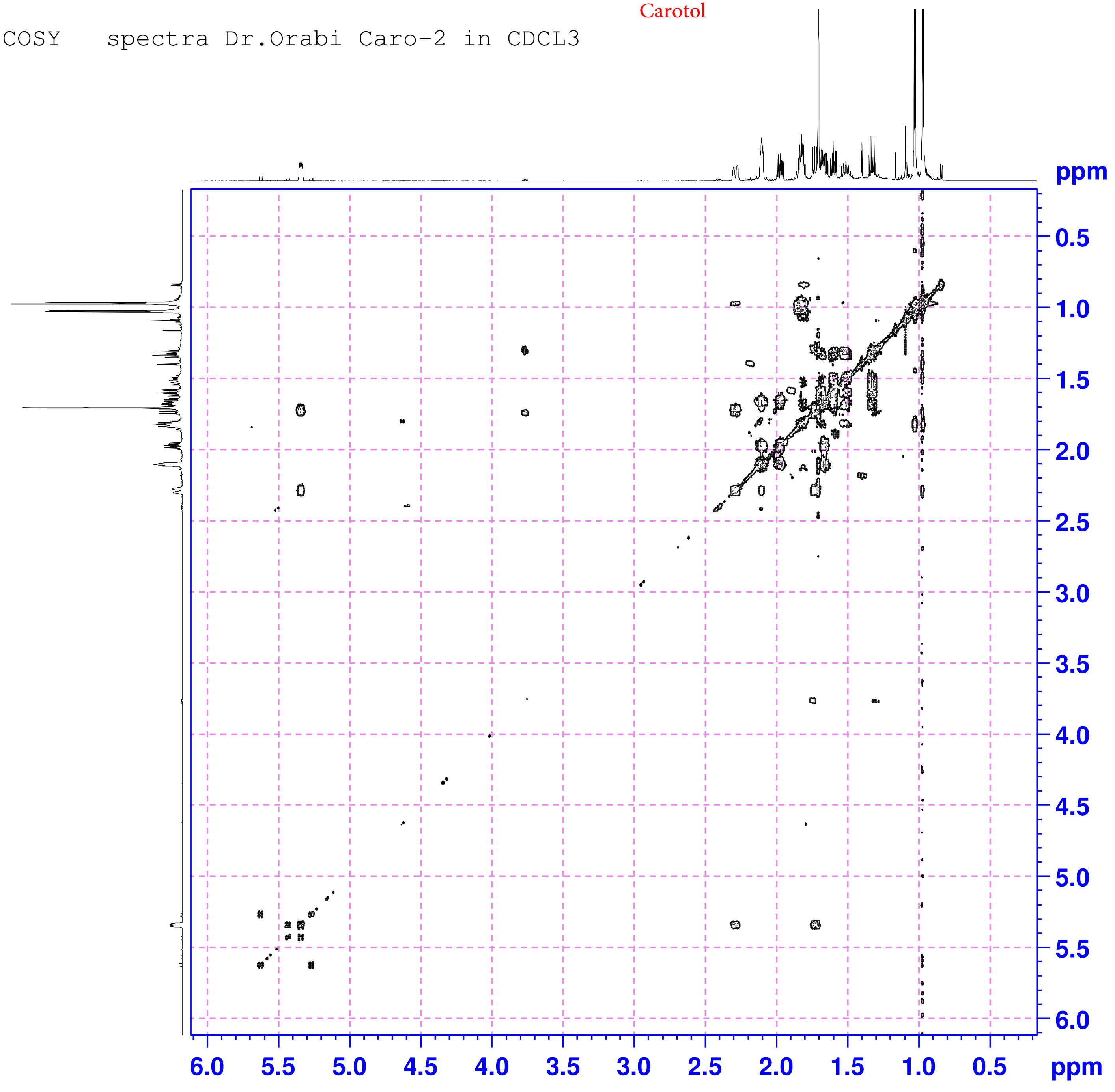


```
Current Data Parameters
NAME              Caro-2-2D
EXPNO                     3
PROCNO                    1

F2 - Acquisition Parameters
Date_              20211021
Time                   5.01
INSTRUM               spect
PROBHD    5 mm PABBO BB-
PULPROG          cosygpppqf
TD                     2048
SOLVENT               CDCl3
NS                        4
DS                        8
SWH                3571.428 Hz
FIDRES             1.743862 Hz
AQ                0.2867200 sec
RG                     90.5
DW                  140.000 usec
DE                     6.50 usec
TE                    298.0 K
D0               0.00000300 sec
D1               0.59107488 sec
D11              0.03000000 sec
D12              0.00002000 sec
D13              0.00000400 sec
D16              0.00020000 sec
IN0              0.00028000 sec

======== CHANNEL f1 ========
SFO1            600.1318862 MHz
NUC1                     1H
P0                    10.60 usec
P1                    10.60 usec
P17                 2500.00 usec
PLW1            27.82500076 W
PLW10            5.00229979 W

====== GRADIENT CHANNEL =====
GPNAM[1]           SINE.100
GPZ1                  20.00 %
P16                 1000.00 usec

F1 - Acquisition parameters
TD                      320
SFO1               600.1319 MHz
FIDRES            11.160714 Hz
SW                    5.951 ppm
FnMODE                   QF

F2 - Processing parameters
SI                     2048
SF              600.1300000 MHz
WDW                    SINE
SSB          0
LB           0 Hz
GB           0
PC                     1.40

F1 - Processing parameters
SI                     2048
MC2                      QF
SF              600.1300000 MHz
WDW                    SINE
SSB          0
LB           0 Hz
GB           0
```

Carotol

COSY spectra Dr.Orabi Caro-2 in CDCL3

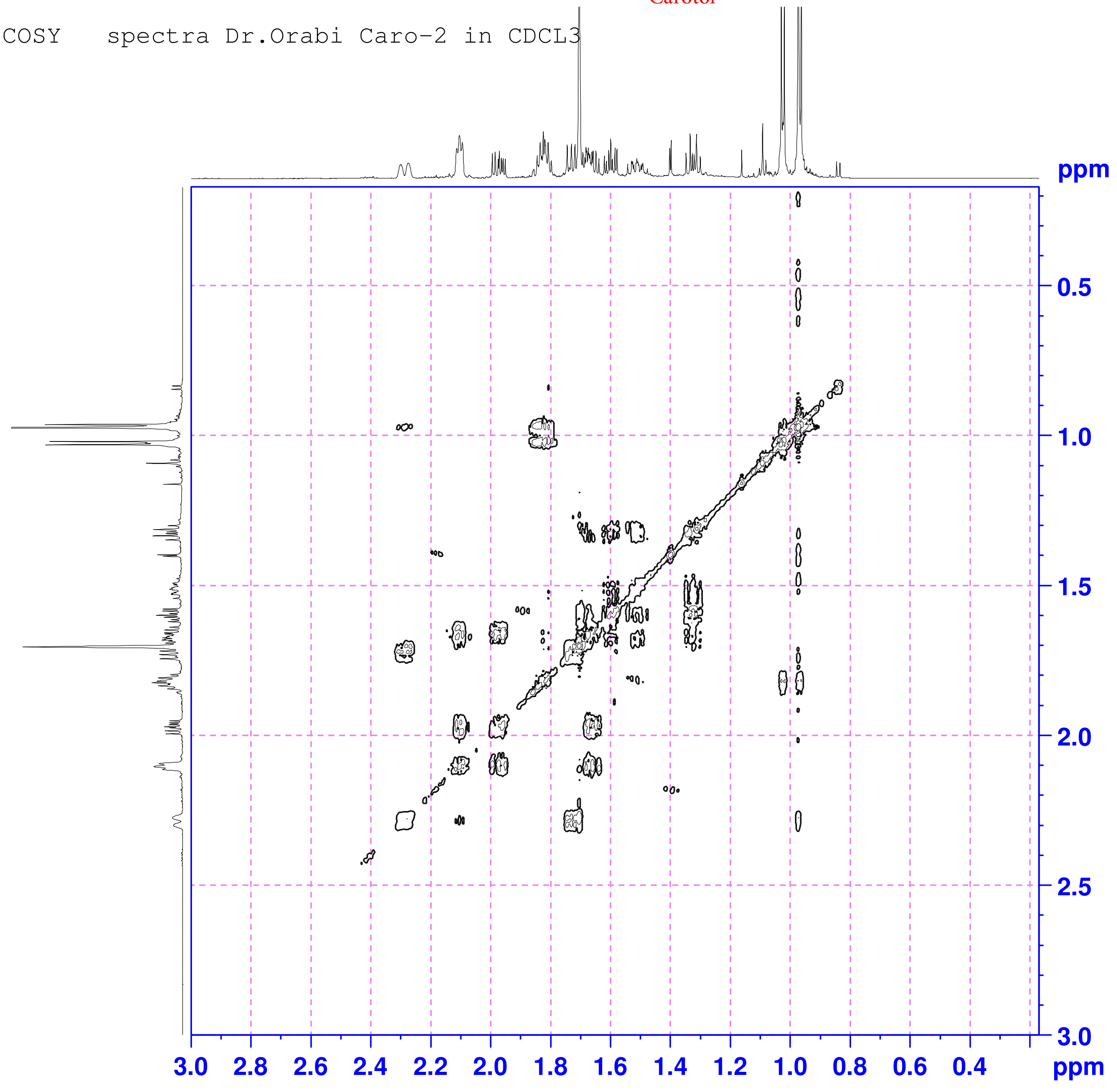

Current Data Parameters

NAME	Caro-2-2D	
EXPNO	3	
PROCNO	1	

F2 - Acquisition Parameters

Date_	20211021	
Time	5.01	
INSTRUM	spect	
PROBHD	5 mm PABBO BB-	
PULPROG	cosygpppqf	
TD	2048	
SOLVENT	CDCl3	
NS	4	
DS	8	
SWH	3571.428	Hz
FIDRES	1.743862	Hz
AQ	0.2867200	sec
RG	90.5	
DW	140.000	usec
DE	6.50	usec
TE	298.0	K
D0	0.00000300	sec
D1	0.59107488	sec
D11	0.03000000	sec
D12	0.00002000	sec
D13	0.00000400	sec
D16	0.00020000	sec
IN0	0.00028000	sec

======== CHANNEL f1 ========

SFO1	600.1318862	MHz
NUC1	1H	
P0	10.60	usec
P1	10.60	usec
P17	2500.00	usec
PLW1	27.82500076	W
PLW10	5.00229979	W

====== GRADIENT CHANNEL =====

GPNAM[1]	SINE.100	
GPZ1	20.00	%
P16	1000.00	usec

F1 - Acquisition parameters

TD	320	
SFO1	600.1319	MHz
FIDRES	11.160714	Hz
SW	5.951	ppm
FnMODE	QF	

F2 - Processing parameters

SI	2048	
SF	600.1300000	MHz
WDW	SINE	
SSB	0	
LB	0	Hz
GB	0	
PC	1.40	

F1 - Processing parameters

SI	2048	
MC2	QF	
SF	600.1300000	MHz
WDW	SINE	
SSB	0	
LB	0	Hz
GB	0	

1H spectra Dr.Orabi HGS -V-Caro-85-A in CDCL3

CM1

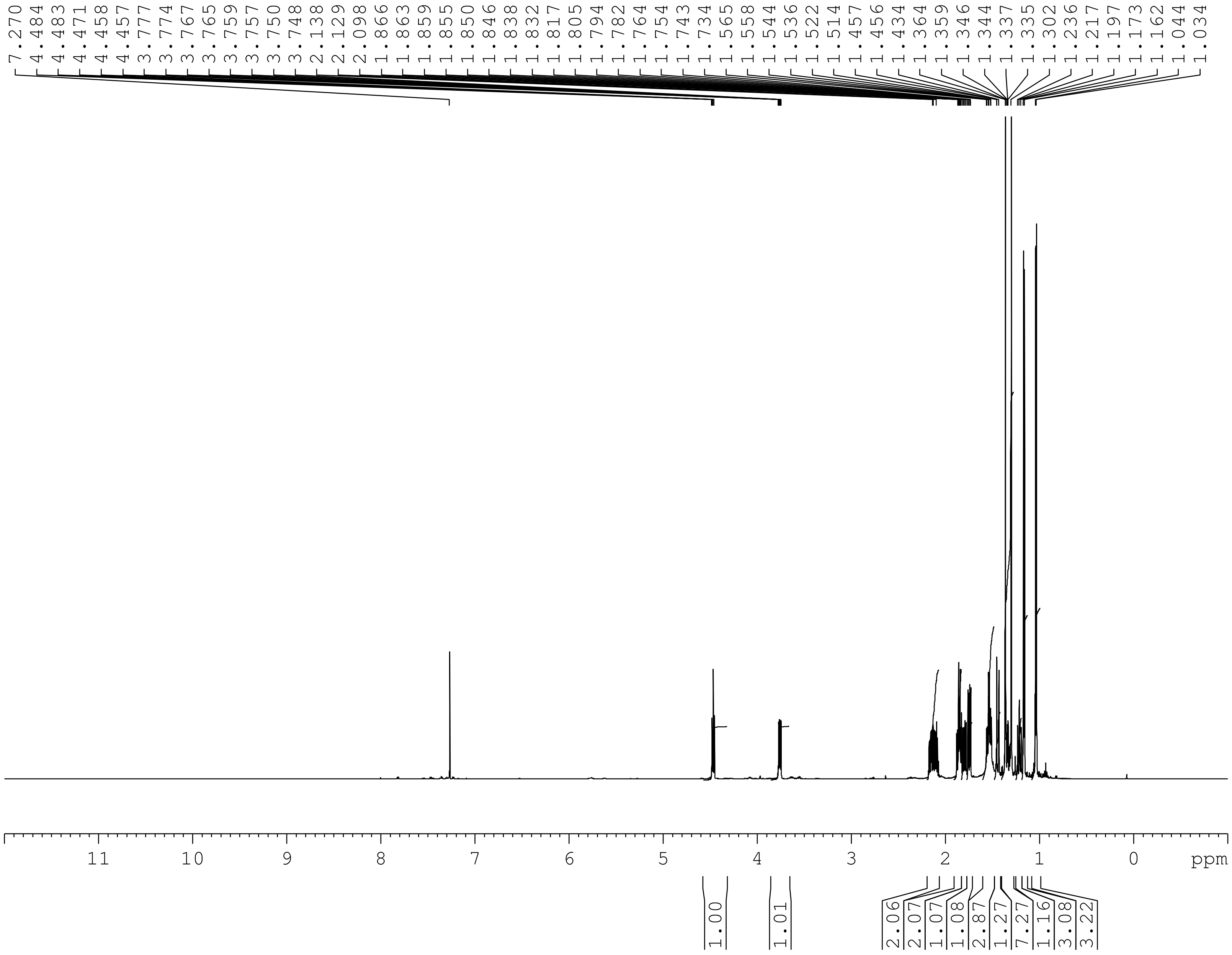

Current	
NAME	HGS-V-Caro85A-2D
EXPNO	1
PROCNO	1
F2 - Acquisition Parameters	
Date_	20220524
Time	11.33
INSTRUM	spect
PROBHD	5 mm PABBO BB-
PULPROG	zg30
TD	65536
SOLVENT	CDCl3
NS	16
DS	2
SWH	12335.526 Hz
FIDRES	0.188225 Hz
AQ	2.6563926 sec
RG	181
DW	40.533 usec
DE	20.00 usec
TE	298.0 K
D1	1.00000000 sec
TD0	1
======== CHANNEL f1 ========	
SFO1	600.1337060 MHz
NUC1	1H
P1	10.60 usec
PLW1	27.82500076 W
F2 - Processing parameters	
SI	32768
SF	600.1300115 MHz
WDW	EM
SSB	0
LB	0.30 Hz
GB	0
PC	1.00

CM1

1H spectra Dr.Orabi HGS -V-Caro-85-A in CDCL3

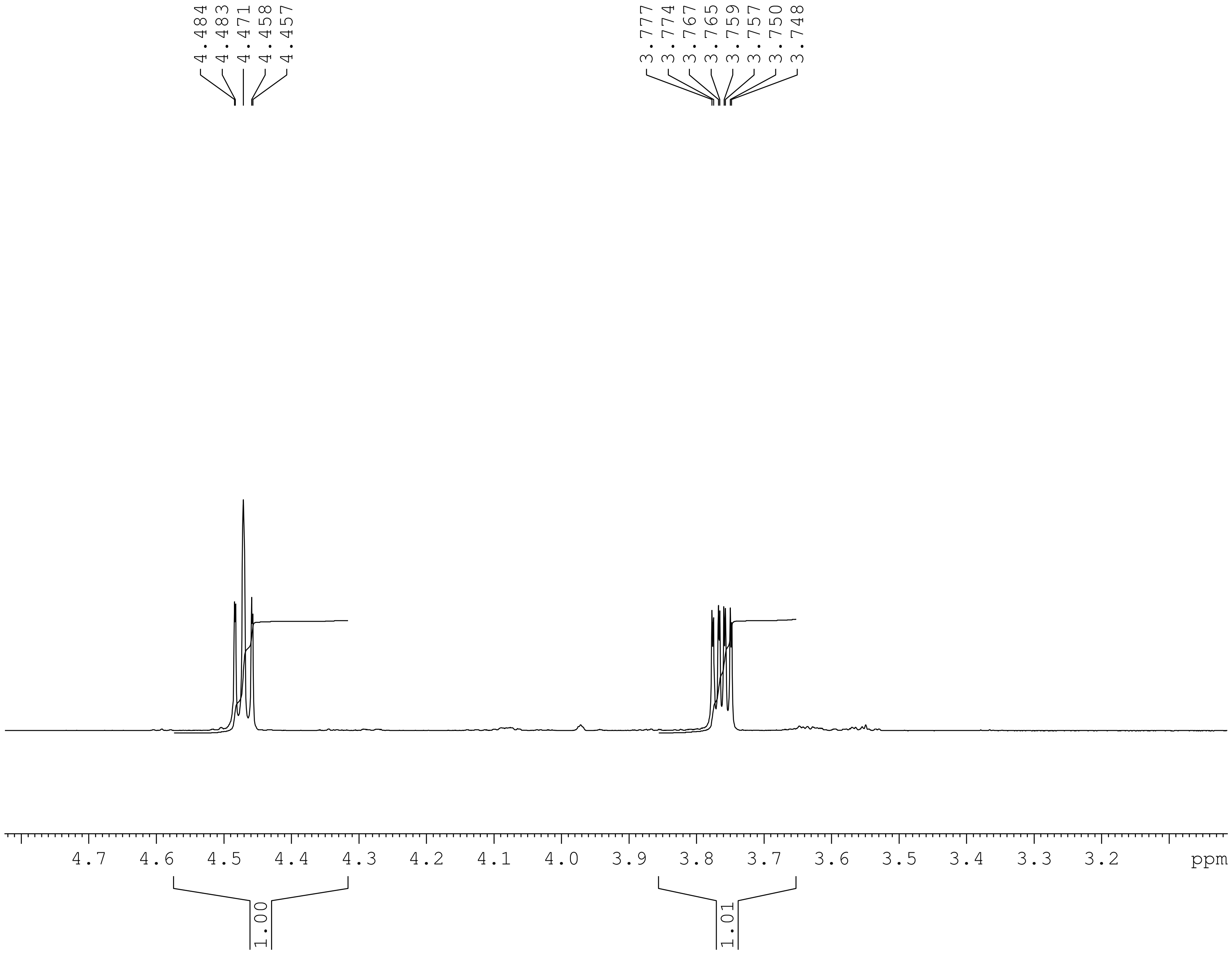

Current	
NAME	HGS-V-Caro85A-2D
EXPNO	1
PROCNO	1
F2 - Acquisition Parameters	
Date_	20220524
Time	11.33
INSTRUM	spect
PROBHD	5 mm PABBO BB-
PULPROG	zg30
TD	65536
SOLVENT	CDCl3
NS	16
DS	2
SWH	12335.526 Hz
FIDRES	0.188225 Hz
AQ	2.6563926 sec
RG	181
DW	40.533 usec
DE	20.00 usec
TE	298.0 K
D1	1.00000000 sec
TD0	1
======== CHANNEL f1 ========	
SFO1	600.1337060 MHz
NUC1	1H
P1	10.60 usec
PLW1	27.82500076 W
F2 - Processing parameters	
SI	32768
SF	600.1300115 MHz
WDW	EM
SSB	0
LB	0.30 Hz
GB	0
PC	1.00

CM1

1H spectra Dr.Orabi HGS -V-Caro-85-A in CDCL3

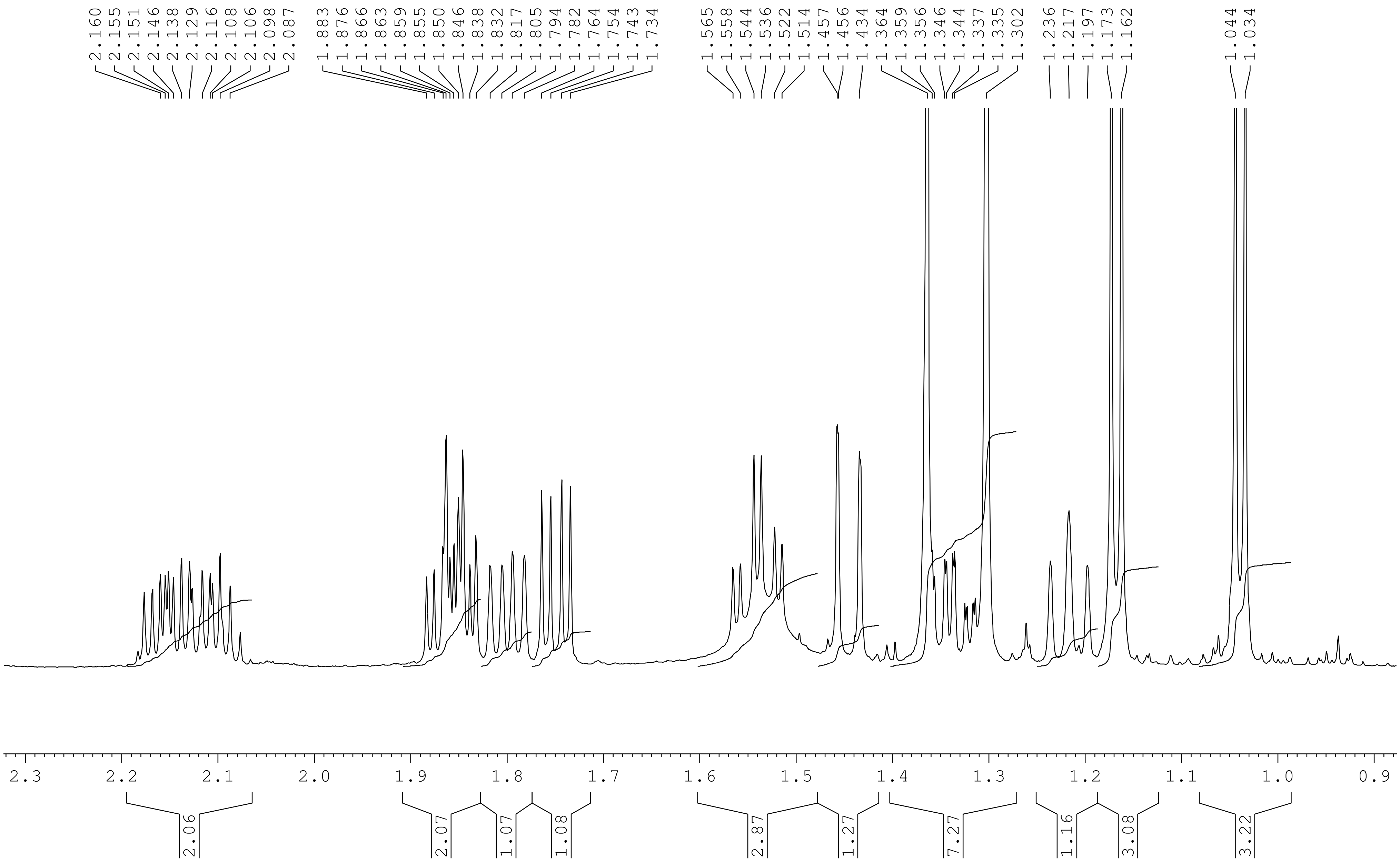

CM1

13C decoupled spectra Dr.Orabi HGS -V-Caro-85-A in CDCL3

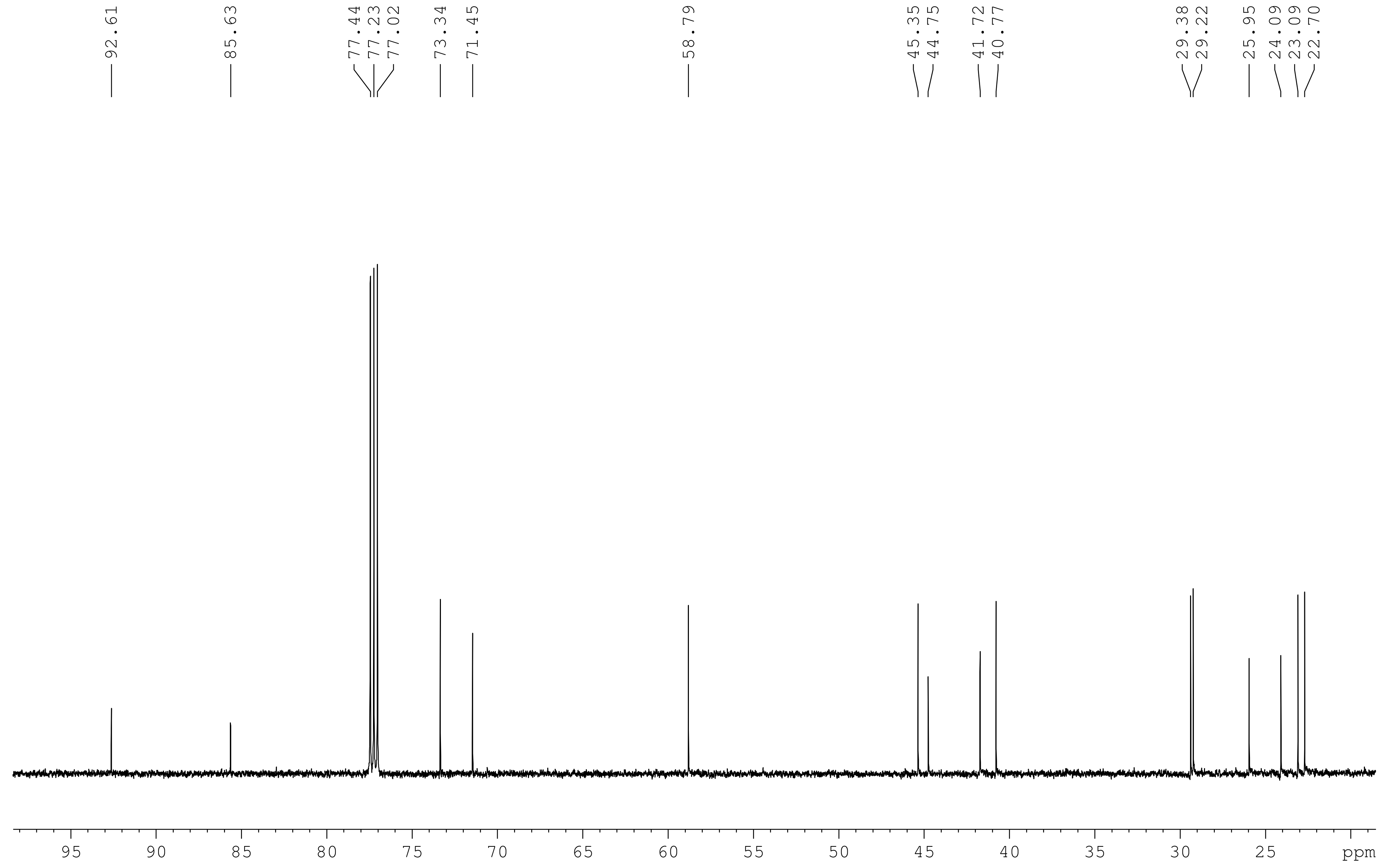

CM1

13C DEPT 135 spectra Dr.Orabi HGS -V-Caro-85-A in CDCL3

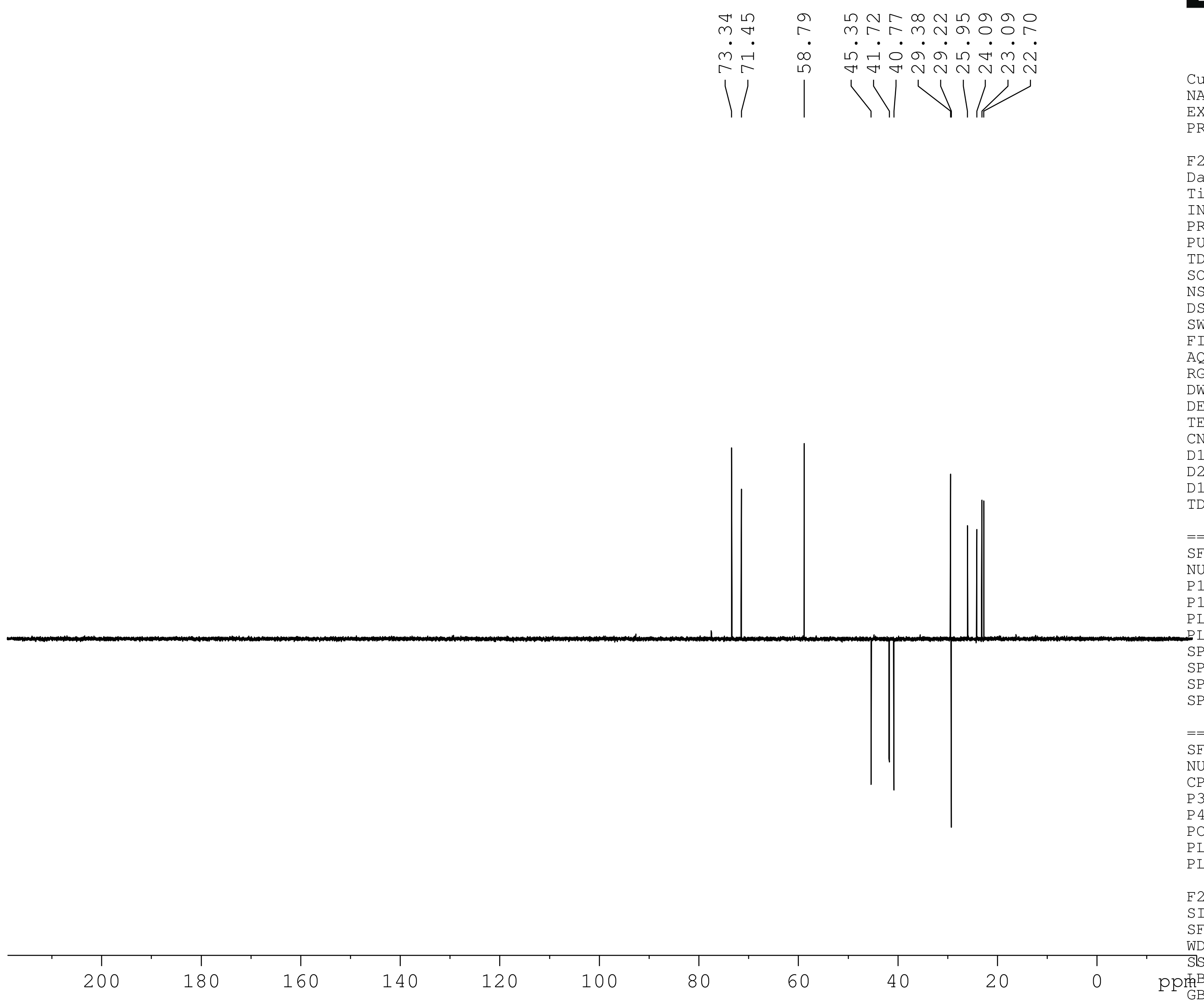


```
Current Data Parameters
NAME       HGS-V-Caro85A-2D
EXPNO                     5
PROCNO                    1

F2 - Acquisition Parameters
Date_              20220524
Time                  23.56
INSTRUM               spect
PROBHD     5 mm PABBO BB-
PULPROG           deptsp135
TD                    65536
SOLVENT               CDCl3
NS                      500
DS                        4
SWH              36057.691 Hz
FIDRES            0.550197 Hz
AQ               0.9087659 sec
RG                      203
DW                   13.867 usec
DE                    50.00 usec
TE                    298.0 K
CNST2           145.0000000
D1               2.00000000 sec
D2               0.00344828 sec
D12              0.00002000 sec
TD0                       1

======== CHANNEL f1 ========
SFO1           150.9178979 MHz
NUC1                    13C
P1                     8.80 usec
P13                 2000.00 usec
PLW0       0 W
PLW1            78.13500214 W
SPNAM[5]        Crp60comp.4
SPOAL5                0.500
SPOFFS5    0 Hz
SPW5             9.24489975 W

======== CHANNEL f2 ========
SFO2           600.1324005 MHz
NUC2                     1H
CPDPRG[2            waltz65
P3                    10.60 usec
P4                    21.20 usec
PCPD2                 70.00 usec
PLW2            27.82500076 W
PLW12            0.63804001 W

F2 - Processing parameters
SI                    32768
SF             150.9027781 MHz
WDW                      EM
SSB        0
LB                     1.00 Hz
GB         0
PC                     1.40
```

CM1

13C HSQC   spectra Dr.Orabi HGS -V-Caro-85-A in CDCL3

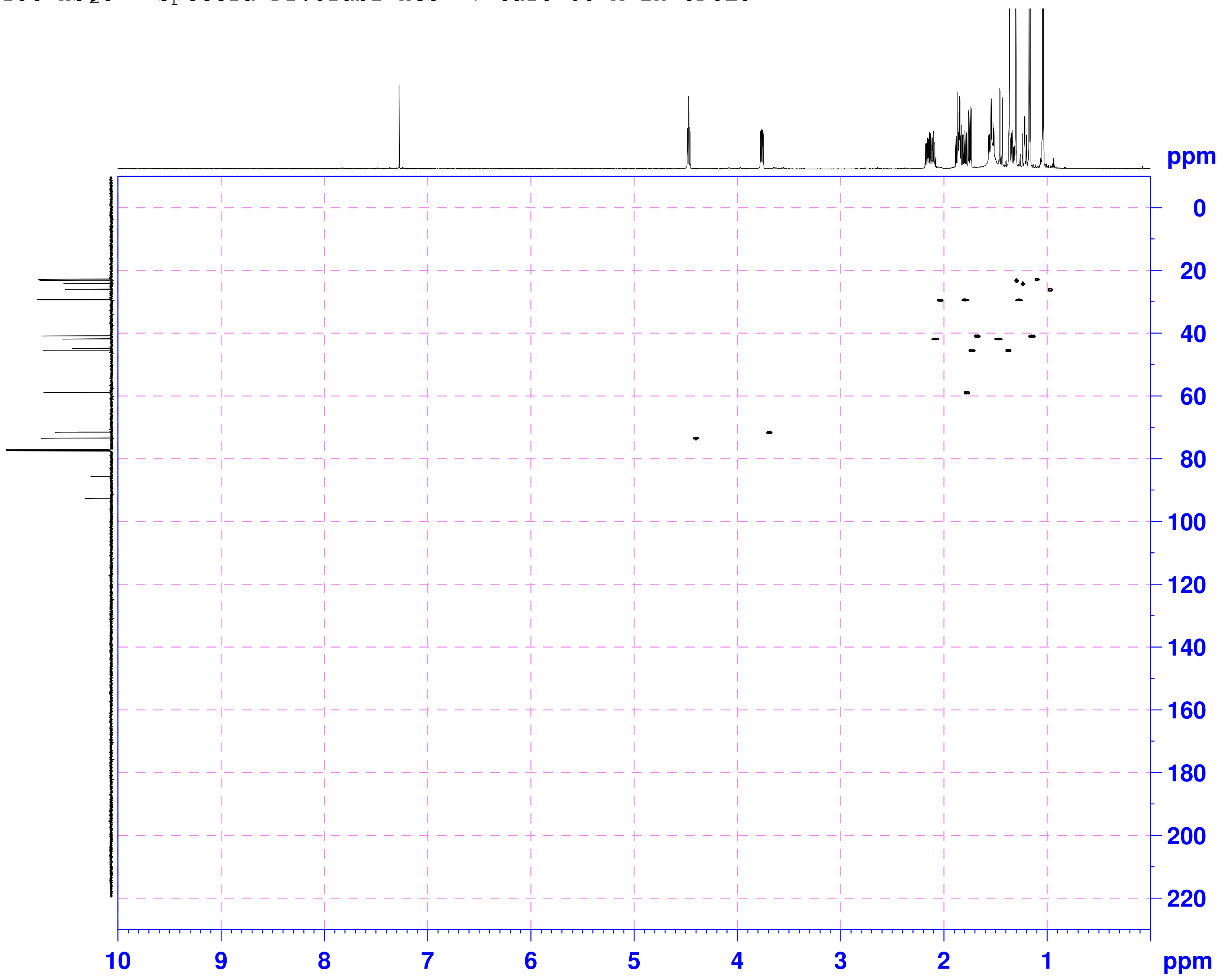


```
Current Data Parameters
NAME      HGS-V-Caro85A-2D
EXPNO              7
PROCNO             1

F2 - Acquisition Parameters
Date_          20220525
Time              0.12
INSTRUM          spect
PROBHD   5 mm PABBO BB-
PULPROG    hsqcetgpsp.2
TD                2048
SOLVENT          CDCl3
NS                   4
DS                  16
SWH           4672.897 Hz
FIDRES        2.281688 Hz
AQ           0.2191360 sec
RG                 203
DW             107.000 usec
DE               20.00 usec
TE               298.1 K
CNST2      145.0000000
D0          0.00000300 sec
D1          1.88735998 sec
D4          0.00172414 sec
D11         0.03000000 sec
D16         0.00020000 sec
IN0         0.00001840 sec
ZGOPTNS

======== CHANNEL f1 ========
SFO1      600.1324588 MHz
NUC1                1H
P1               10.60 usec
P2               21.20 usec
P28            1000.00 usec
PLW1       27.82500076 W

======== CHANNEL f2 ========
SFO2      150.9148803 MHz
NUC2               13C
CPDPRG[2         garp4
P3                8.80 usec
P14             500.00 usec
P24            2000.00 usec
PCPD2            60.00 usec
PLW0      0 W
PLW2       78.13500214 W
PLW12       1.68079996 W
SPNAM[3] Crp60,0.5,20.1
SPOAL3           0.500
SPOFFS3   0 Hz
SPW3        9.24489975 W
SPNAM[7]    Crp60comp.4
SPOAL7           0.500
SPOFFS7   0 Hz
SPW7        9.24489975 W

====== GRADIENT CHANNEL =====
GPNAM[1]      SINE.100
GPNAM[2]      SINE.100
GPZ1             80.00 %
GPZ2             20.10 %
P16            1000.00 usec

F1 - Acquisition parameters
TD                 512
SFO1          150.9149 MHz
FIDRES       53.074047 Hz
SW             180.061 ppm
FnMODE    Echo-Antiecho

F2 - Processing parameters
SI                2048
SF         600.1300553 MHz
WDW              QSINE
SSB                  2
LB        0 Hz
GB        0
PC                1.40

F1 - Processing parameters
SI                1024
MC2        echo-antiecho
SF         150.9027623 MHz
WDW              QSINE
SSB                  2
LB        0 Hz
```

CM1

13C HSQC spectra Dr.Orabi HGS -V-Caro-85-A in CDCL3

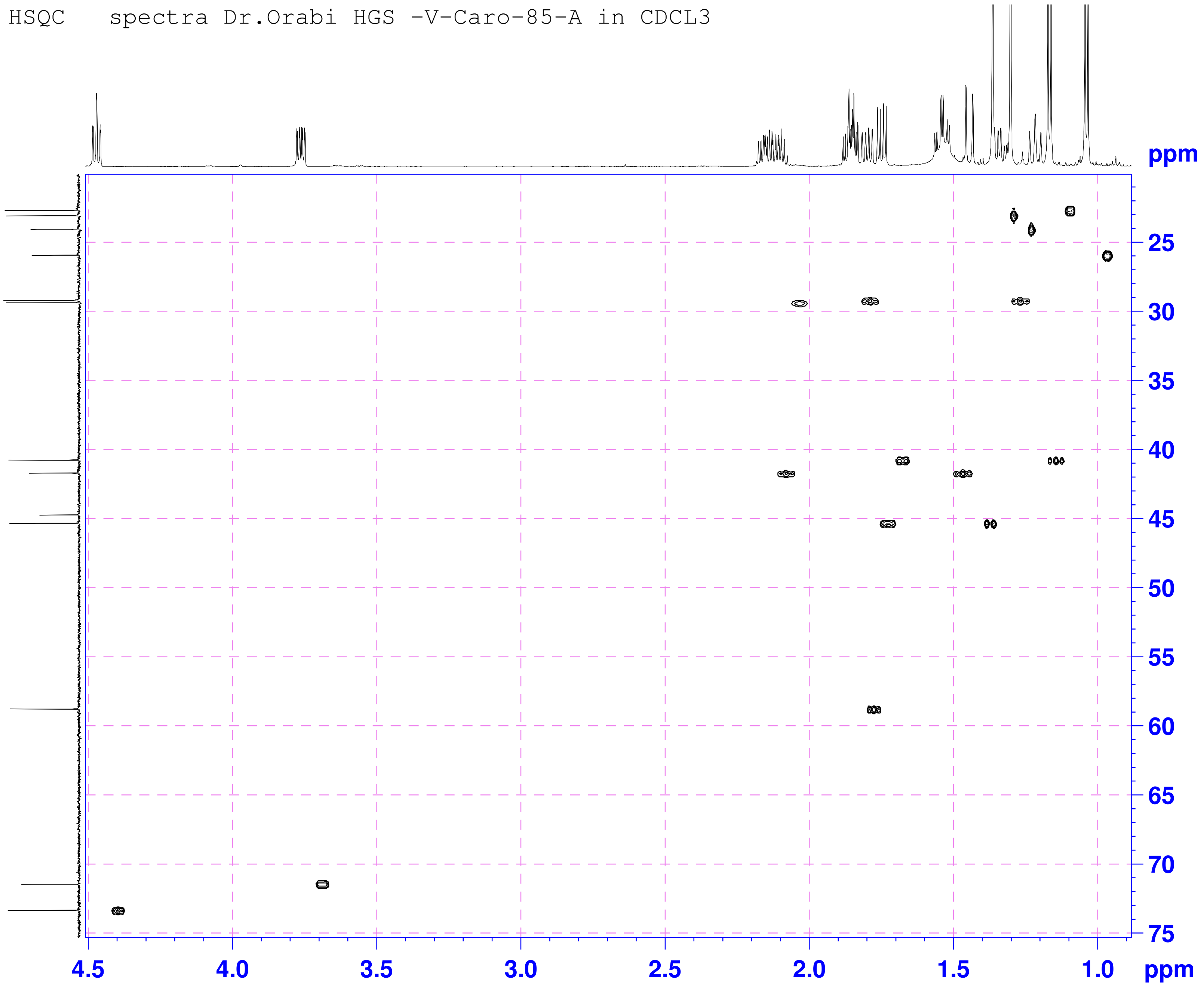


```
Current Data Parameters
NAME      HGS-V-Caro85A-2D
EXPNO                    7
PROCNO                   1

F2 - Acquisition Parameters
Date_            20220525
Time                 0.12
INSTRUM             spect
PROBHD   5 mm PABBO BB-
PULPROG     hsqcetgpsp.2
TD                   2048
SOLVENT             CDCl3
NS                      4
DS                     16
SWH              4672.897 Hz
FIDRES           2.281688 Hz
AQ              0.2191360 sec
RG                    203
DW                107.000 usec
DE                  20.00 usec
TE                  298.1 K
CNST2         145.0000000
D0             0.00000300 sec
D1             1.88735998 sec
D4             0.00172414 sec
D11            0.03000000 sec
D16            0.00020000 sec
IN0            0.00001840 sec
ZGOPTNS

======== CHANNEL f1 ========
SFO1         600.1324588 MHz
NUC1                  1H
P1                 10.60 usec
P2                 21.20 usec
P28              1000.00 usec
PLW1         27.82500076 W

======== CHANNEL f2 ========
SFO2         150.9148803 MHz
NUC2                 13C
CPDPRG[2           garp4
P3                  8.80 usec
P14               500.00 usec
P24              2000.00 usec
PCPD2              60.00 usec
PLW0       0 W
PLW2         78.13500214 W
PLW12         1.68079996 W
SPNAM[3] Crp60,0.5,20.1
SPOAL3             0.500
SPOFFS3    0 Hz
SPW3          9.24489975 W
SPNAM[7]    Crp60comp.4
SPOAL7             0.500
SPOFFS7    0 Hz
SPW7          9.24489975 W

====== GRADIENT CHANNEL =====
GPNAM[1]        SINE.100
GPNAM[2]        SINE.100
GPZ1               80.00 %
GPZ2               20.10 %
P16              1000.00 usec

F1 - Acquisition parameters
TD                   512
SFO1            150.9149 MHz
FIDRES         53.074047 Hz
SW               180.061 ppm
FnMODE     Echo-Antiecho

F2 - Processing parameters
SI                  2048
SF           600.1300553 MHz
WDW                QSINE
SSB                    2
LB         0 Hz
GB         0
PC                  1.40

F1 - Processing parameters
SI                  1024
MC2         echo-antiecho
SF           150.9027623 MHz
WDW                QSINE
SSB                    2
LB         0 Hz
```

CM1

13C HMBC   spectra Dr.Orabi HGS -V-Caro-85-A in CDCL3

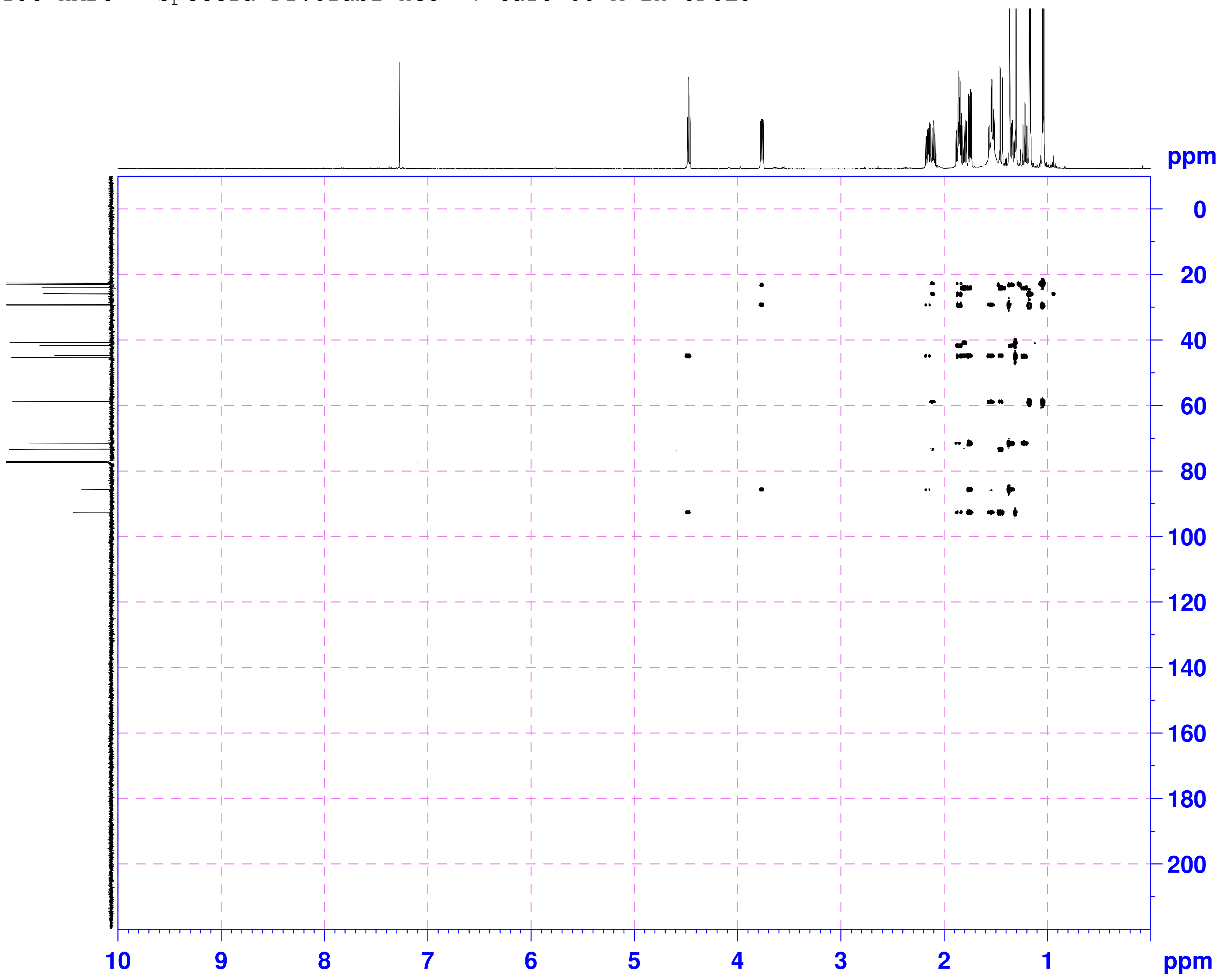

Current Data Parameters

NAME	HGS-V-Caro85A-2D
EXPNO	8
PROCNO	1

F2 - Acquisition Parameters

Date_	20220525
Time	1.28
INSTRUM	spect
PROBHD	5 mm PABBO BB-
PULPROG	hmbcetgpl2nd
TD	2048
SOLVENT	CDCl3
NS	16
DS	16
SWH	4672.897 Hz
FIDRES	2.281688 Hz
AQ	0.2191360 sec
RG	203
DW	107.000 usec
DE	20.00 usec
TE	297.9 K
CNST6	125.0000000
CNST7	165.0000000
CNST13	10.0000000
CNST30	0.5981151
D0	0.00000300 sec
D1	1.41193604 sec
D6	0.05000000 sec
D16	0.00020000 sec
IN0	0.00001490 sec

======== CHANNEL f1 ========

SFO1	600.1324588 MHz
NUC1	1H
P1	10.60 usec
P2	21.20 usec
PLW1	27.82500076 W

======== CHANNEL f2 ========

SFO2	150.9178738 MHz
NUC2	13C
P3	8.80 usec
P24	2000.00 usec
PLW2	78.13500214 W
SPNAM[7]	Crp60comp.4
SPOAL7	0.500
SPOFFS7	0 Hz
SPW7	9.24489975 W

====== GRADIENT CHANNEL =====

GPNAM[1]	SINE.100
GPNAM[3]	SINE.100
GPNAM[4]	SINE.100
GPNAM[5]	SINE.100
GPZ1	80.00 %
GPZ3	15.00 %
GPZ4	-10.00 %
GPZ5	-5.00 %
P16	1000.00 usec

F1 - Acquisition parameters

TD	256
SFO1	150.9179 MHz
FIDRES	131.082214 Hz
SW	222.353 ppm
FnMODE	Echo-Antiecho

F2 - Processing parameters

SI	2048
SF	600.1300104 MHz
WDW	SINE
SSB	0
LB	0 Hz
GB	0
PC	1.40

F1 - Processing parameters

SI	512
MC2	echo-antiecho
SF	150.9027493 MHz
WDW	SINE
SSB	2
LB	0 Hz

CM1

13C HMBC   spectra Dr.Orabi HGS -V-Caro-85-A in CDCL3

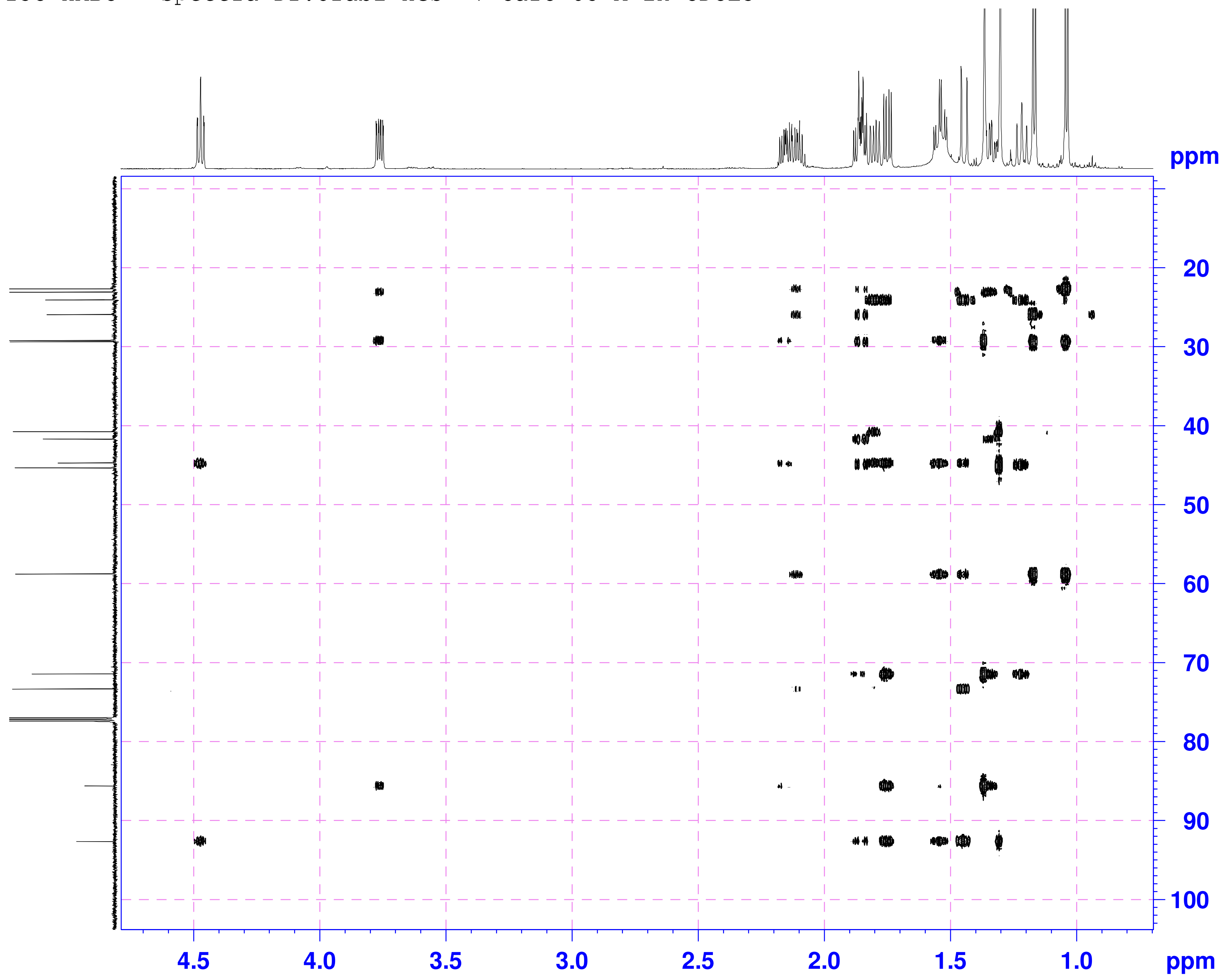

BRUKER

```
Current Data Parameters
NAME      HGS-V-Caro85A-2D
EXPNO                    8
PROCNO                   1

F2 - Acquisition Parameters
Date_             20220525
Time                  1.28
INSTRUM              spect
PROBHD    5 mm PABBO BB-
PULPROG     hmbcetgpl2nd
TD                    2048
SOLVENT              CDCl3
NS                      16
DS                      16
SWH               4672.897 Hz
FIDRES            2.281688 Hz
AQ               0.2191360 sec
RG                     203
DW                 107.000 usec
DE                   20.00 usec
TE                   297.9 K
CNST6          125.0000000
CNST7          165.0000000
CNST13          10.0000000
CNST30           0.5981151
D0              0.00000300 sec
D1              1.41193604 sec
D6              0.05000000 sec
D16             0.00020000 sec
IN0             0.00001490 sec

======== CHANNEL f1 ========
SFO1           600.1324588 MHz
NUC1                    1H
P1                   10.60 usec
P2                   21.20 usec
PLW1           27.82500076 W

======== CHANNEL f2 ========
SFO2           150.9178738 MHz
NUC2                   13C
P3                    8.80 usec
P24                2000.00 usec
PLW2           78.13500214 W
SPNAM[7]       Crp60comp.4
SPOAL7               0.500
SPOFFS7   0 Hz
SPW7            9.24489975 W

====== GRADIENT CHANNEL =====
GPNAM[1]          SINE.100
GPNAM[3]          SINE.100
GPNAM[4]          SINE.100
GPNAM[5]          SINE.100
GPZ1                 80.00 %
GPZ3                 15.00 %
GPZ4                -10.00 %
GPZ5                 -5.00 %
P16                1000.00 usec

F1 - Acquisition parameters
TD                     256
SFO1              150.9179 MHz
FIDRES          131.082214 Hz
SW                 222.353 ppm
FnMODE     Echo-Antiecho

F2 - Processing parameters
SI                    2048
SF             600.1300104 MHz
WDW                   SINE
SSB       0
LB        0 Hz
GB        0
PC                    1.40

F1 - Processing parameters
SI                     512
MC2          echo-antiecho
SF             150.9027493 MHz
WDW                   SINE
SSB                      2
LB        0 Hz
```

CM1

13C HMBC spectra Dr.Orabi HGS -V-Caro-85-A in CDCL3

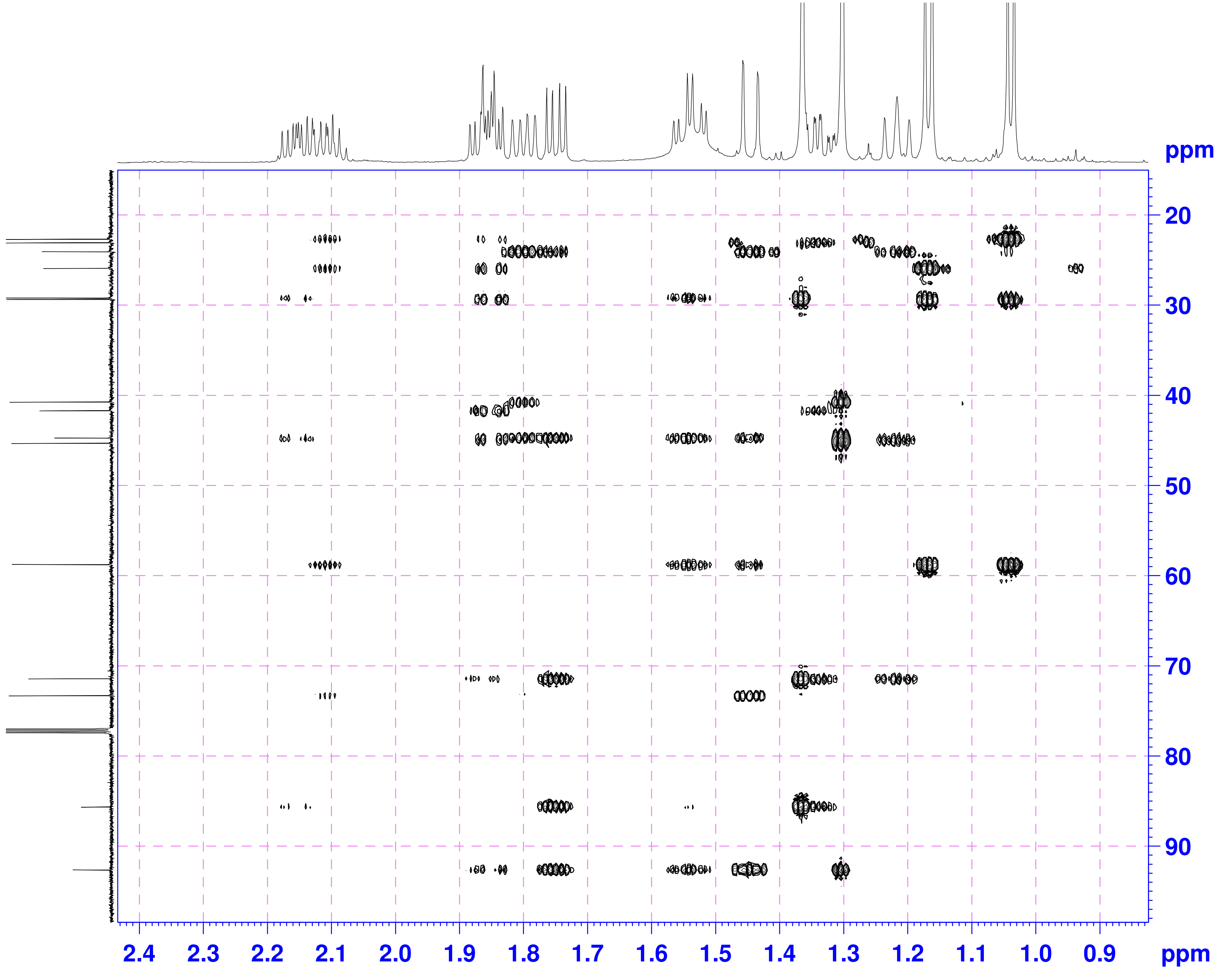


```
Current Data Parameters
NAME      HGS-V-Caro85A-2D
EXPNO                    8
PROCNO                   1

F2 - Acquisition Parameters
Date_             20220525
Time                  1.28
INSTRUM              spect
PROBHD   5 mm PABBO BB-
PULPROG     hmbcetgpl2nd
TD                    2048
SOLVENT              CDCl3
NS                      16
DS                      16
SWH               4672.897 Hz
FIDRES            2.281688 Hz
AQ               0.2191360 sec
RG                     203
DW                 107.000 usec
DE                   20.00 usec
TE                   297.9 K
CNST6          125.0000000
CNST7          165.0000000
CNST13          10.0000000
CNST30           0.5981151
D0              0.00000300 sec
D1              1.41193604 sec
D6              0.05000000 sec
D16             0.00020000 sec
IN0             0.00001490 sec

======== CHANNEL f1 ========
SFO1        600.1324588 MHz
NUC1                     1H
P1                    10.60 usec
P2                    21.20 usec
PLW1            27.82500076 W

======== CHANNEL f2 ========
SFO2        150.9178738 MHz
NUC2                    13C
P3                     8.80 usec
P24                 2000.00 usec
PLW2            78.13500214 W
SPNAM[7]       Crp60comp.4
SPOAL7                0.500
SPOFFS7   0 Hz
SPW7             9.24489975 W

====== GRADIENT CHANNEL =====
GPNAM[1]          SINE.100
GPNAM[3]          SINE.100
GPNAM[4]          SINE.100
GPNAM[5]          SINE.100
GPZ1                 80.00 %
GPZ3                 15.00 %
GPZ4                -10.00 %
GPZ5                 -5.00 %
P16                1000.00 usec

F1 - Acquisition parameters
TD                     256
SFO1              150.9179 MHz
FIDRES          131.082214 Hz
SW                 222.353 ppm
FnMODE        Echo-Antiecho

F2 - Processing parameters
SI                    2048
SF             600.1300104 MHz
WDW                   SINE
SSB       0
LB        0 Hz
GB        0
PC                    1.40

F1 - Processing parameters
SI                     512
MC2           echo-antiecho
SF             150.9027493 MHz
WDW                   SINE
SSB                      2
LB        0 Hz
```

CM1

COSY spectra Dr.Orabi HGS -V-Caro-85-A in CDCL3

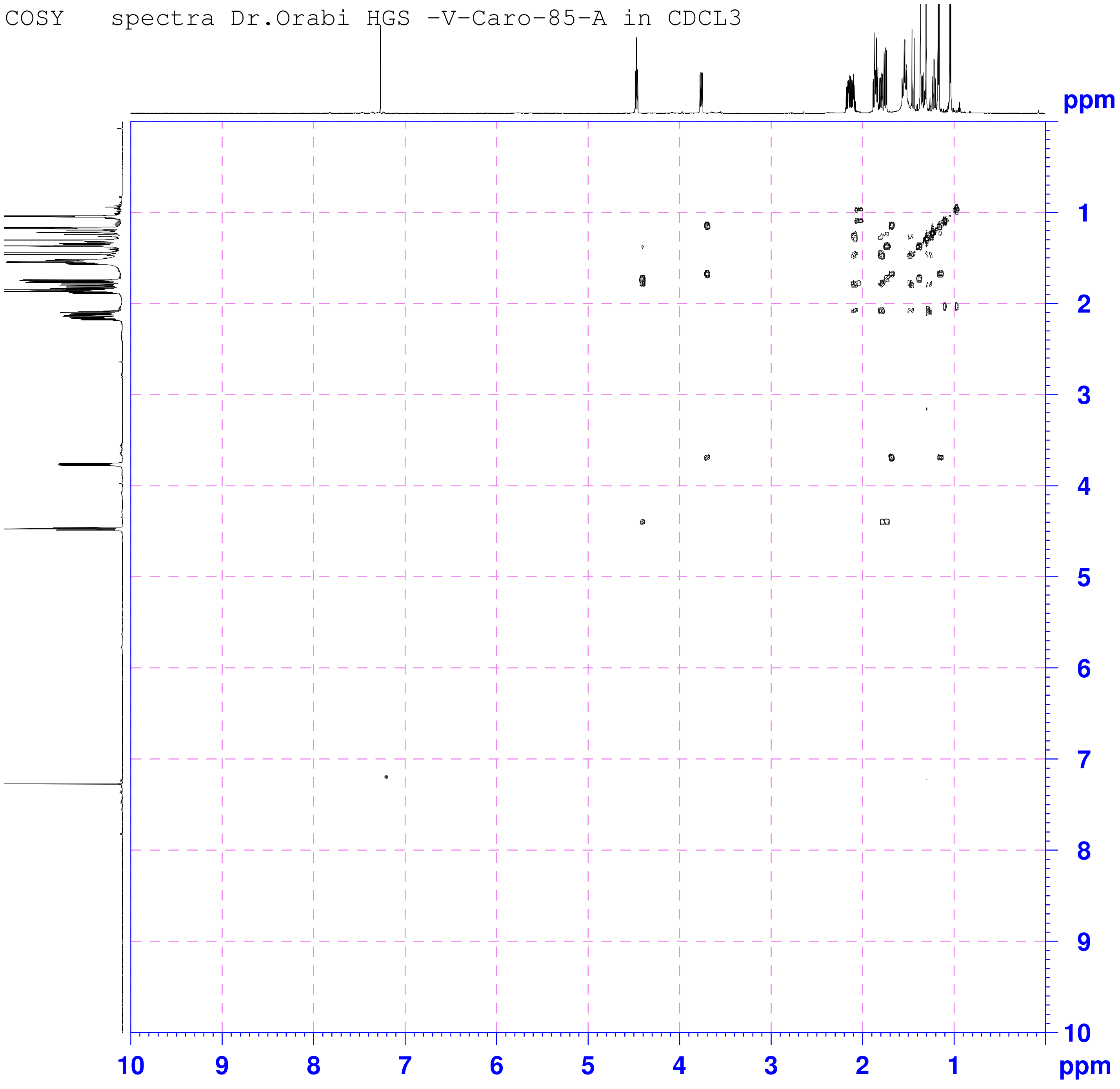


```
Current Data Parameters
NAME      HGS-V-Caro85A-2D
EXPNO                    6
PROCNO                   1

F2 - Acquisition Parameters
Date_             20220524
Time                 23.58
INSTRUM              spect
PROBHD    5 mm PABBO BB-
PULPROG        cosygpppqf
TD                    2048
SOLVENT              CDCl3
NS                       2
DS                       8
SWH              4672.897 Hz
FIDRES           2.281688 Hz
AQ              0.2191360 sec
RG                    90.5
DW                 107.000 usec
DE                   20.00 usec
TE                   297.9 K
D0              0.00000300 sec
D1              0.65865898 sec
D11             0.03000000 sec
D12             0.00002000 sec
D13             0.00000400 sec
D16             0.00020000 sec
IN0             0.00021400 sec

======== CHANNEL f1 ========
SFO1         600.1323807 MHz
NUC1                    1H
P0                   10.60 usec
P1                   10.60 usec
P17                2500.00 usec
PLW1           27.82500076 W
PLW10           4.62489986 W

====== GRADIENT CHANNEL =====
GPNAM[1]          SINE.100
GPZ1                 20.00 %
P16                1000.00 usec

F1 - Acquisition parameters
TD                     320
SFO1              600.1324 MHz
FIDRES           14.602804 Hz
SW                   7.786 ppm
FnMODE                  QF

F2 - Processing parameters
SI                    2048
SF            600.1300549 MHz
WDW                   SINE
SSB       0
LB        0 Hz
GB        0
PC                    1.40

F1 - Processing parameters
SI                    2048
MC2                     QF
SF            600.1300578 MHz
WDW                   SINE
SSB       0
LB        0 Hz
GB        0
```

CM1

COSY spectra Dr.Orabi HGS -V-Caro-85-A in CDCL3

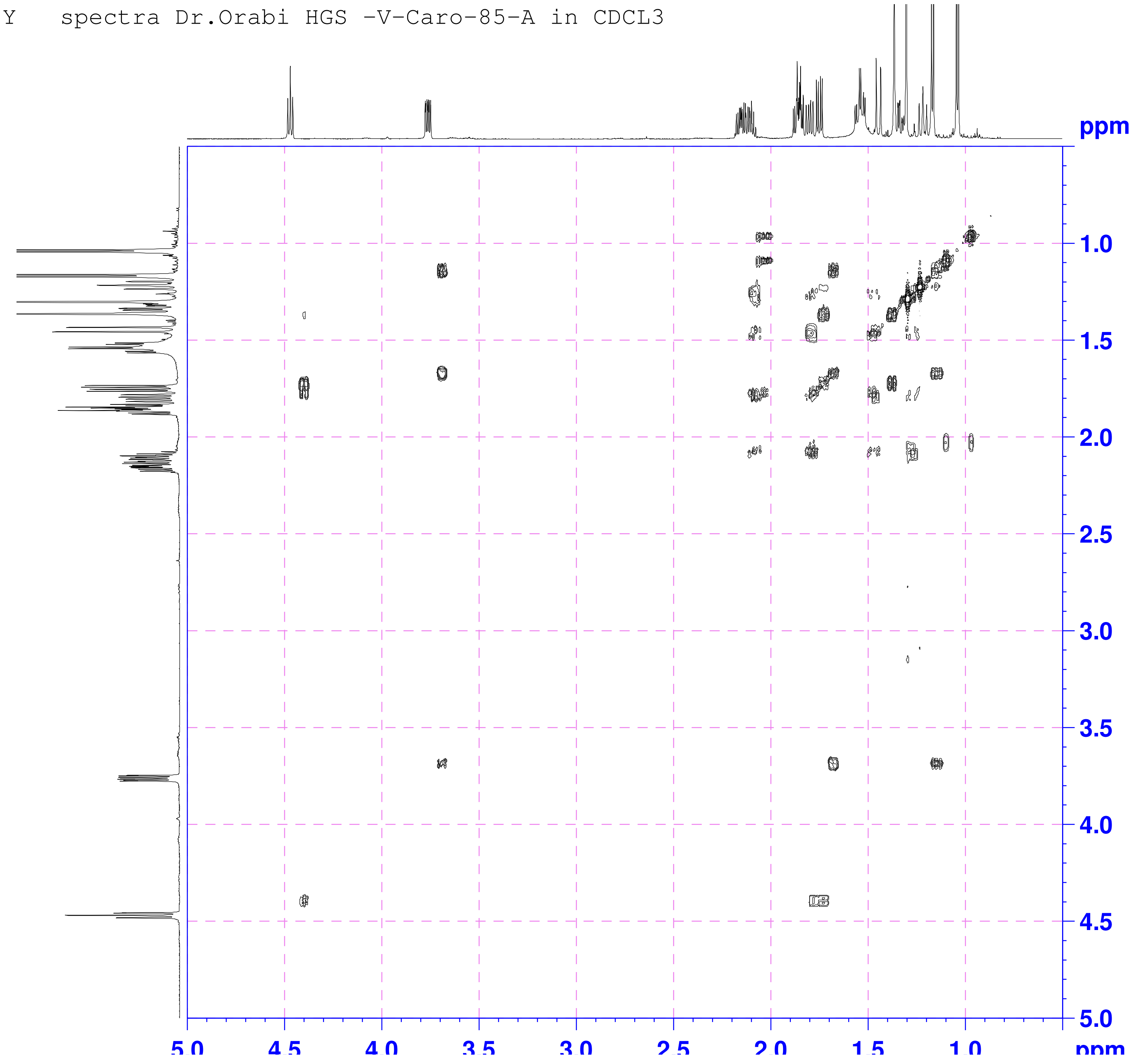


```
Current Data Parameters
NAME      HGS-V-Caro85A-2D
EXPNO                    6
PROCNO                   1

F2 - Acquisition Parameters
Date_             20220524
Time                 23.58
INSTRUM              spect
PROBHD    5 mm PABBO BB-
PULPROG         cosygpppqf
TD                    2048
SOLVENT              CDCl3
NS                       2
DS                       8
SWH               4672.897 Hz
FIDRES            2.281688 Hz
AQ               0.2191360 sec
RG                    90.5
DW                 107.000 usec
DE                   20.00 usec
TE                   297.9 K
D0              0.00000300 sec
D1              0.65865898 sec
D11             0.03000000 sec
D12             0.00002000 sec
D13             0.00000400 sec
D16             0.00020000 sec
IN0             0.00021400 sec

======== CHANNEL f1 ========
SFO1           600.1323807 MHz
NUC1                    1H
P0                   10.60 usec
P1                   10.60 usec
P17                2500.00 usec
PLW1           27.82500076 W
PLW10           4.62489986 W

====== GRADIENT CHANNEL =====
GPNAM[1]          SINE.100
GPZ1                 20.00 %
P16                1000.00 usec

F1 - Acquisition parameters
TD                     320
SFO1              600.1324 MHz
FIDRES           14.602804 Hz
SW                   7.786 ppm
FnMODE                  QF

F2 - Processing parameters
SI                    2048
SF             600.1300549 MHz
WDW                   SINE
SSB        0
LB         0 Hz
GB         0
PC                    1.40

F1 - Processing parameters
SI                    2048
MC2                     QF
SF             600.1300578 MHz
WDW                   SINE
SSB        0
LB         0 Hz
GB         0
```

CM1

COSY spectra Dr.Orabi HGS -V-Caro-85-A in CDCL3

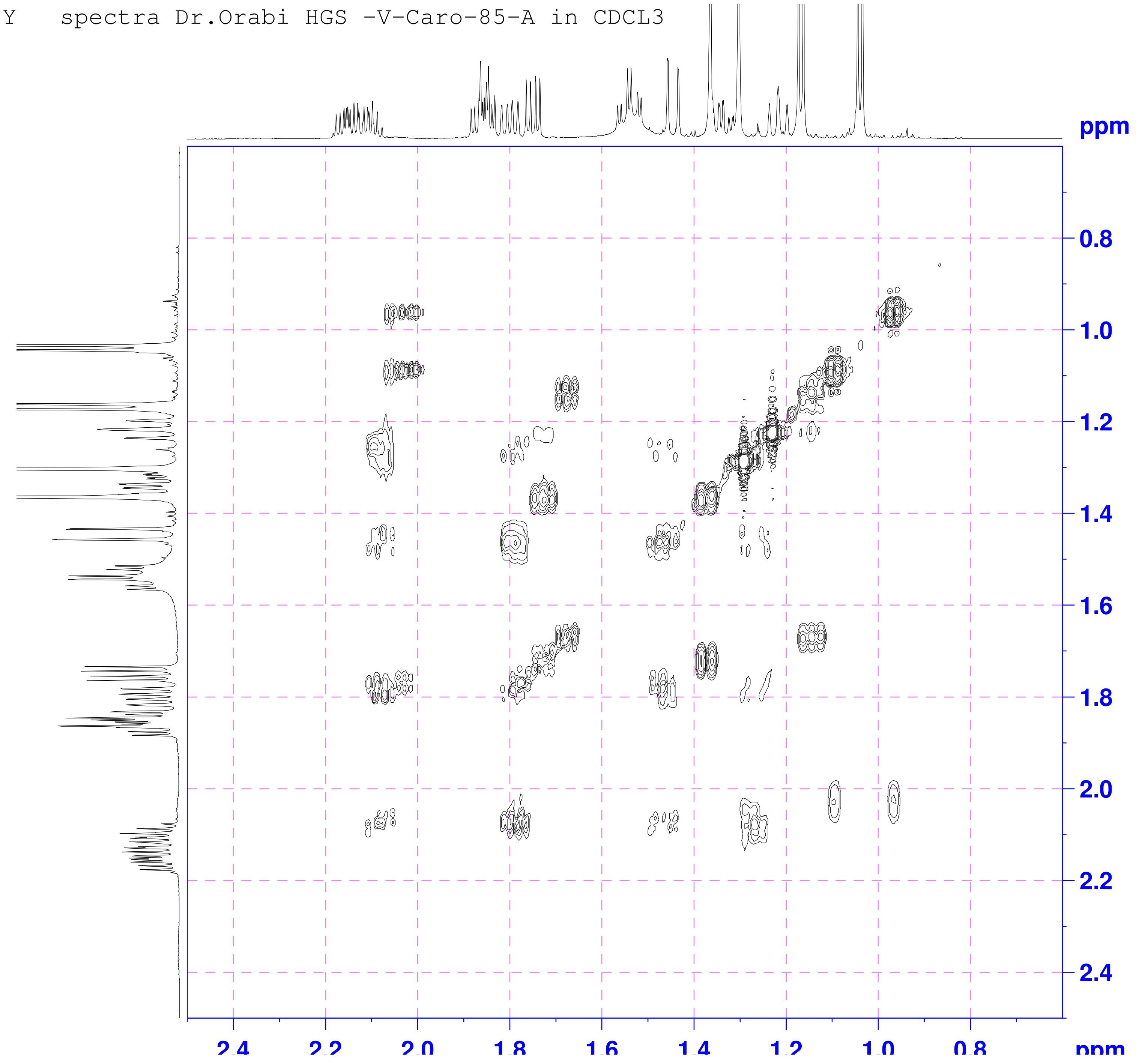


```
Current Data Parameters
NAME        HGS-V-Caro85A-2D
EXPNO                      6
PROCNO                     1

F2 - Acquisition Parameters
Date_               20220524
Time                   23.58
INSTRUM                spect
PROBHD     5 mm PABBO BB-
PULPROG           cosygpppqf
TD                      2048
SOLVENT                CDCl3
NS                         2
DS                         8
SWH                 4672.897 Hz
FIDRES              2.281688 Hz
AQ                 0.2191360 sec
RG                      90.5
DW                   107.000 usec
DE                     20.00 usec
TE                     297.9 K
D0                0.00000300 sec
D1                0.65865898 sec
D11               0.03000000 sec
D12               0.00002000 sec
D13               0.00000400 sec
D16               0.00020000 sec
IN0               0.00021400 sec

======== CHANNEL f1 ========
SFO1          600.1323807 MHz
NUC1                       1H
P0                      10.60 usec
P1                      10.60 usec
P17                   2500.00 usec
PLW1             27.82500076 W
PLW10             4.62489986 W

====== GRADIENT CHANNEL =====
GPNAM[1]            SINE.100
GPZ1                   20.00 %
P16                  1000.00 usec

F1 - Acquisition parameters
TD                       320
SFO1                600.1324 MHz
FIDRES             14.602804 Hz
SW                     7.786 ppm
FnMODE                    QF

F2 - Processing parameters
SI                      2048
SF               600.1300549 MHz
WDW                     SINE
SSB      0
LB       0 Hz
GB       0
PC                      1.40

F1 - Processing parameters
SI                      2048
MC2                       QF
SF               600.1300578 MHz
WDW                     SINE
SSB      0
LB       0 Hz
GB       0
```

CM2

1H SPECTRUM DR.ORABI HGS V caro 86 IN CDCL3

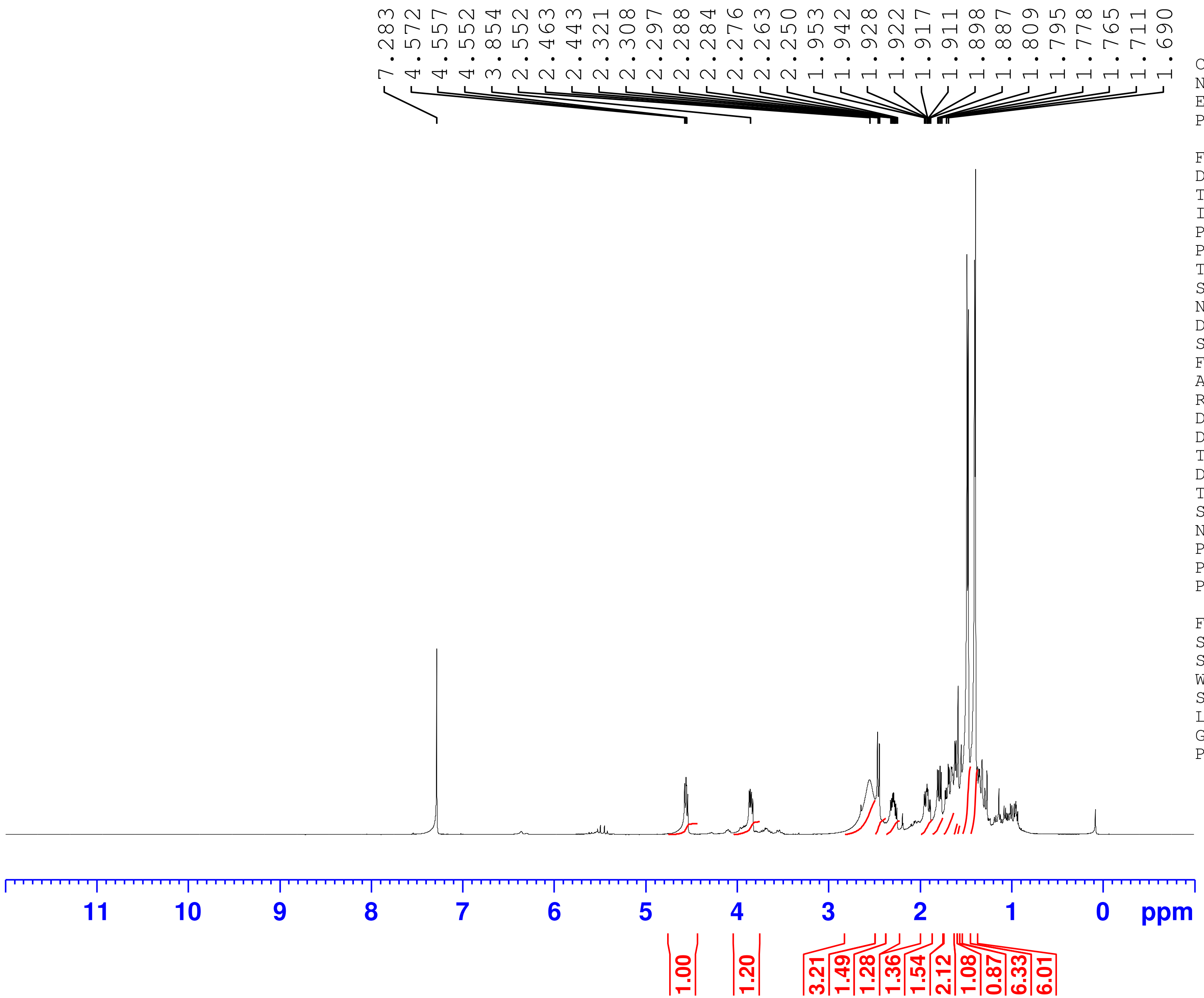

Current Data Parameters
NAME HGSVcaro86-2D
EXPNO 10
PROCNO 1

F2 - Acquisition Parameters
Date_ 20220522
Time 14.12 h
INSTRUM Avance AV Neo 400
PROBHD Z108618_0912 (
PULPROG zg30
TD 65536
SOLVENT CH3OH
NS 16
DS 2
SWH 8196.722 Hz
FIDRES 0.250144 Hz
AQ 3.9976959 sec
RG 101
DW 61.000 usec
DE 12.86 usec
TE 298.0 K
D1 1.00000000 sec
TD0 1
SFO1 400.1324708 MHz
NUC1 1H
P0 4.67 usec
P1 14.00 usec
PLW1 13.01399994 W

F2 - Processing parameters
SI 65536
SF 400.1300000 MHz
WDW EM
SSB 0
LB 0.30 Hz
GB 0
PC 1.00

CM2

1H SPECTRUM DR.ORABI HGS V caro 86 IN CDCL3

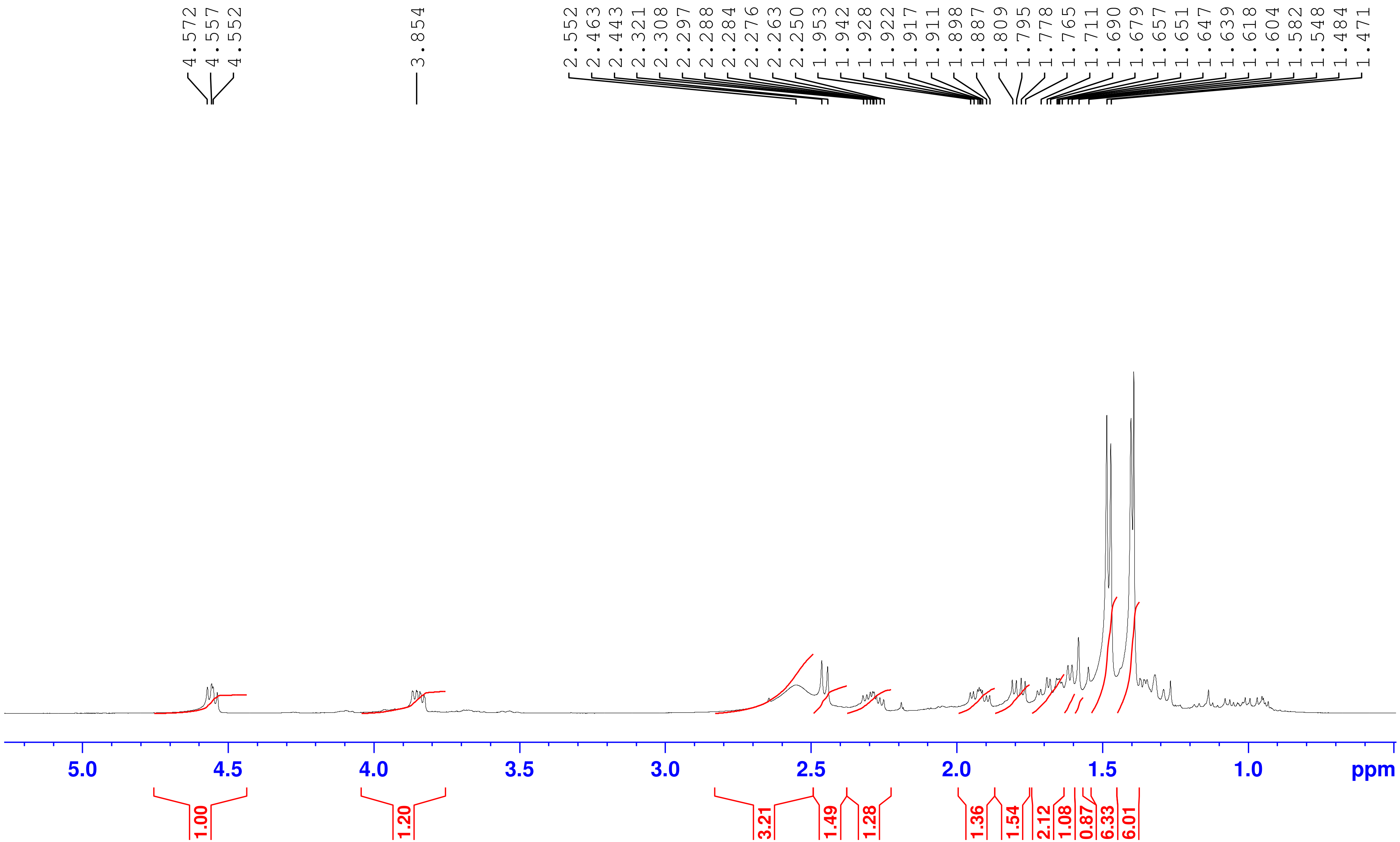

CM2

13C DECOUPLED SPECTRUM DR.ORABI HGS V caro 86 IN CDCL3

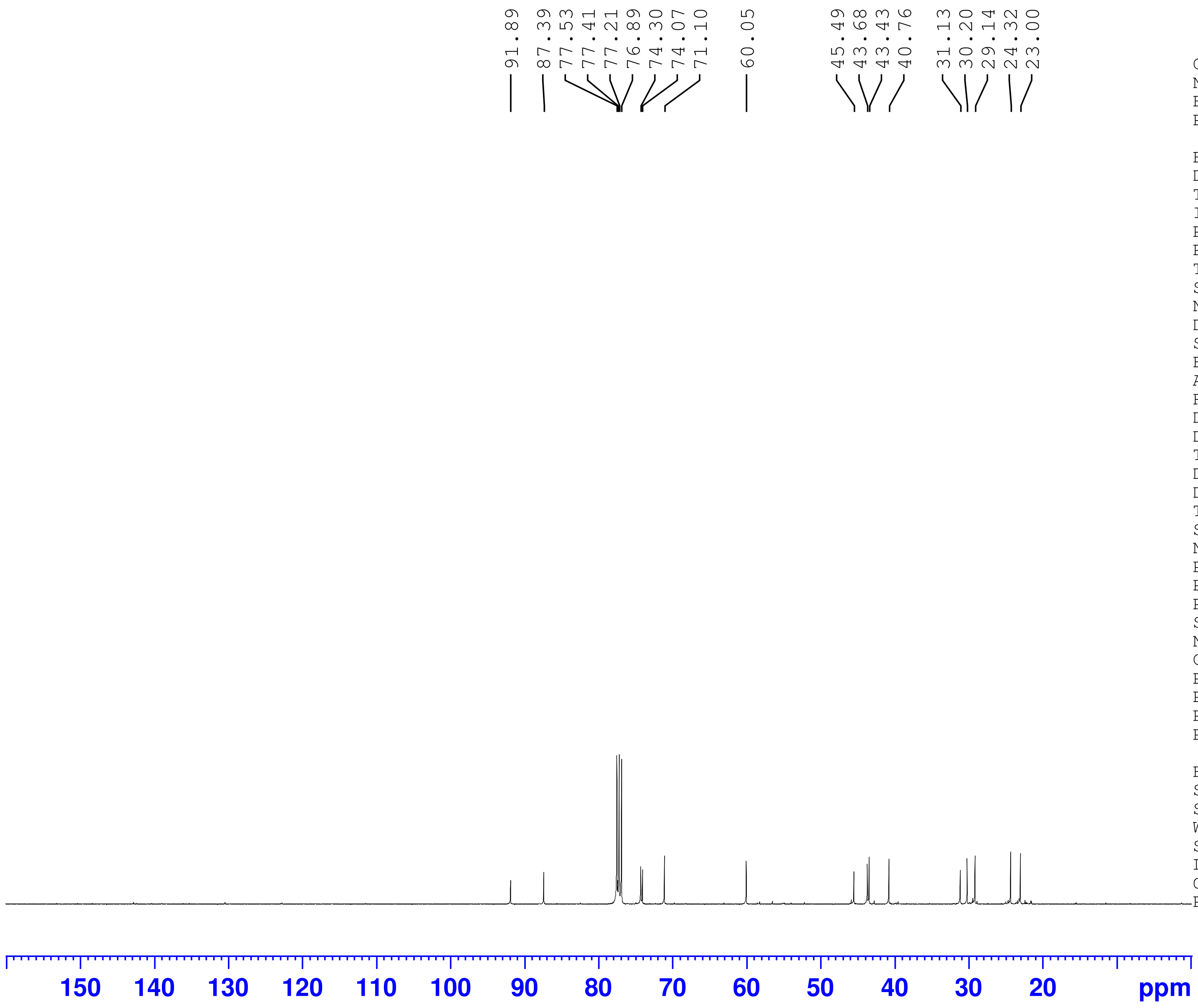

Current Data Parameters
NAME HGSVcaro86-2D
EXPNO 11
PROCNO 1

F2 - Acquisition Parameters
Date_ 20220523
Time 5.21 h
INSTRUM Avance AV Neo 400
PROBHD Z108618_0912 (
PULPROG zgpg30
TD 65536
SOLVENT CH3OH
NS 15360
DS 4
SWH 23809.523 Hz
FIDRES 0.726609 Hz
AQ 1.3762560 sec
RG 29.8117
DW 21.000 usec
DE 6.50 usec
TE 298.9 K
D1 2.00000000 sec
D11 0.03000000 sec
TD0 1
SFO1 100.6228298 MHz
NUC1 13C
P0 3.33 usec
P1 10.00 usec
PLW1 53.78799820 W
SFO2 400.1316005 MHz
NUC2 1H
CPDPRG[2 waltz65
PCPD2 90.00 usec
PLW2 13.01399994 W
PLW12 0.31492001 W
PLW13 0.15840000 W

F2 - Processing parameters
SI 32768
SF 100.6127514 MHz
WDW EM
SSB 0
LB 1.00 Hz
GB 0
PC 1.40

CM2

13C DECOUPLED SPECTRUM DR.ORABI HGS V caro 86 IN CDCL3

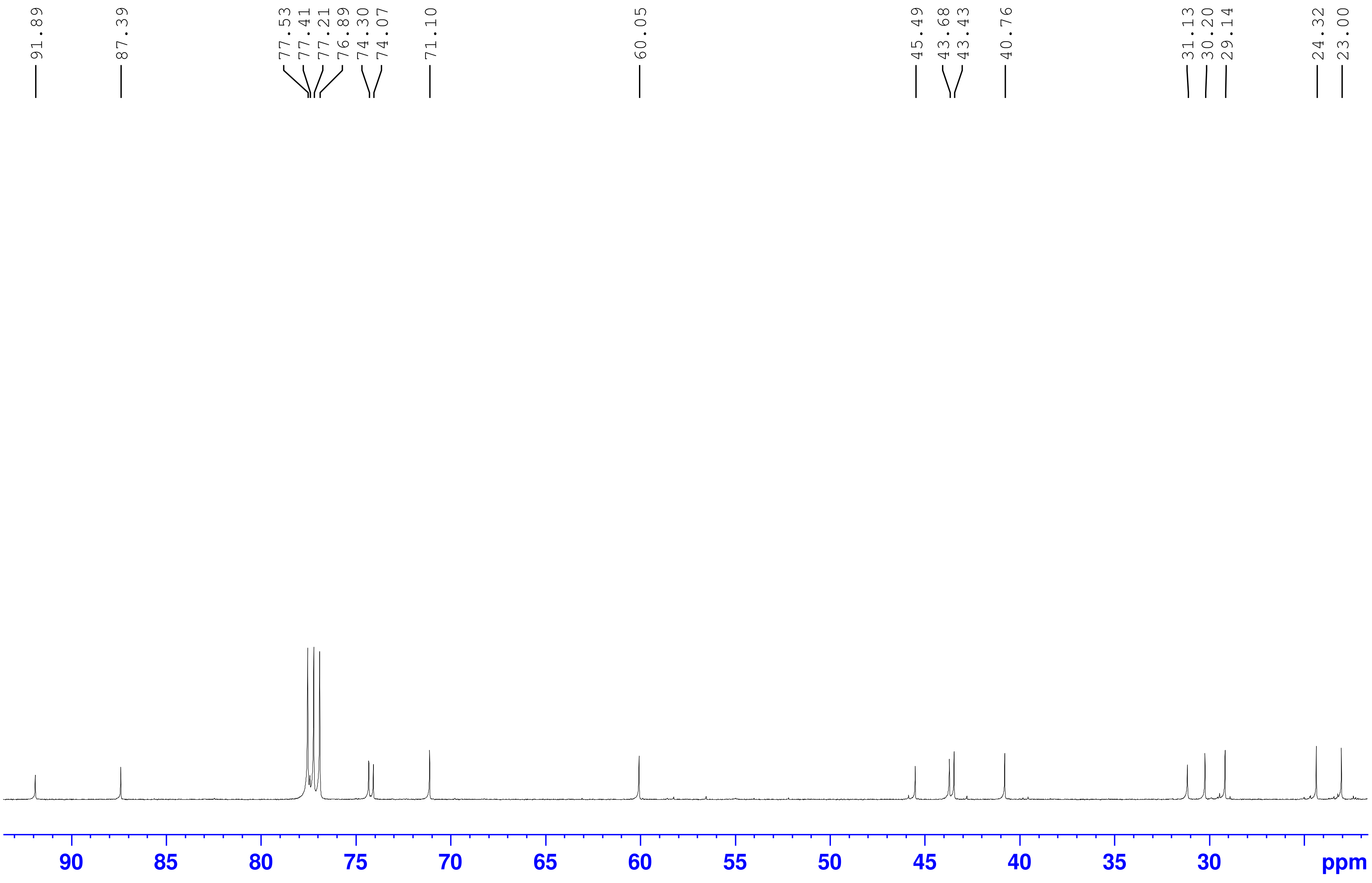

CM2

DEPT 135 SPECTRUM DR.ORABI HGS V caro 86 IN CDCL3

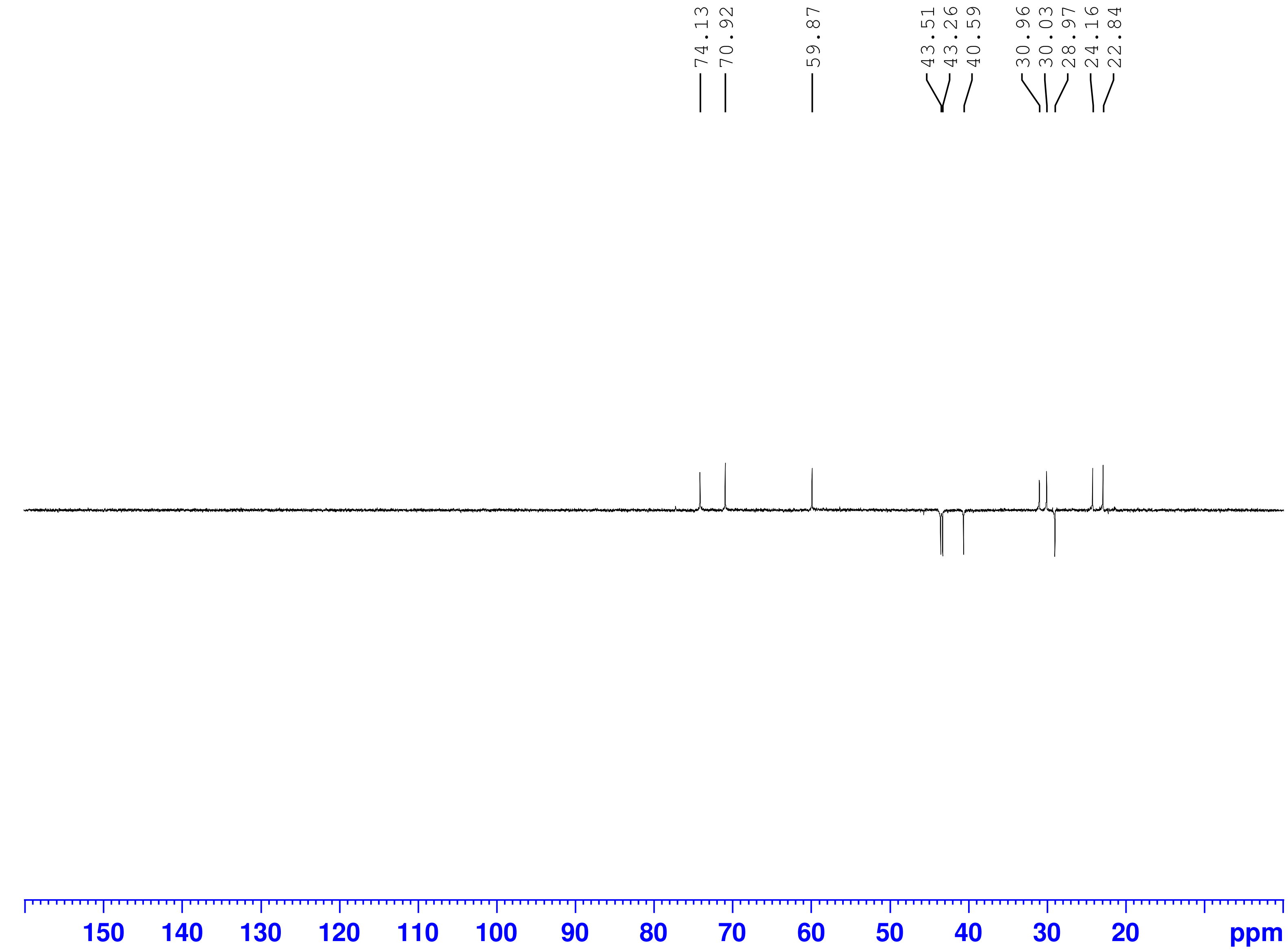

Current Data Parameters

NAME	HGSVcaro86-2D
EXPNO	16
PROCNO	1

F2 - Acquisition Parameters

Date_	20220523
Time	12.57 h
INSTRUM	Avance AV Neo 400
PROBHD	Z108618_0912 (
PULPROG	deptsp135
TD	65536
SOLVENT	CH3OH
NS	256
DS	8
SWH	16129.032 Hz
FIDRES	0.492219 Hz
AQ	2.0316160 sec
RG	46.3268
DW	31.000 usec
DE	6.50 usec
TE	298.5 K
CNST2	145.0000000
D1	2.00000000 sec
D2	0.00344828 sec
D12	0.00002000 sec
TD0	1
SFO1	100.6208175 MHz
NUC1	13C
P1	10.00 usec
P13	2000.00 usec
PLW0	0 W
PLW1	53.78799820 W
SPNAM[5]	Crp60comp.4
SPOAL5	0.500
SPOFFS5	0 Hz
SPW5	8.21819973 W
SFO2	400.1316005 MHz
NUC2	1H
CPDPRG[2	waltz65
P3	14.00 usec
P4	28.00 usec
PCPD2	90.00 usec
PLW2	13.01399994 W
PLW12	0.31492001 W

F2 - Processing parameters

SI	32768
SF	100.6127685 MHz
WDW	EM
SSB	0
LB	1.00 Hz
GB	0
PC	1.40

CM2

HSQC SPECTRUM DR.ORABI HGS V caro 86 IN CDCL3

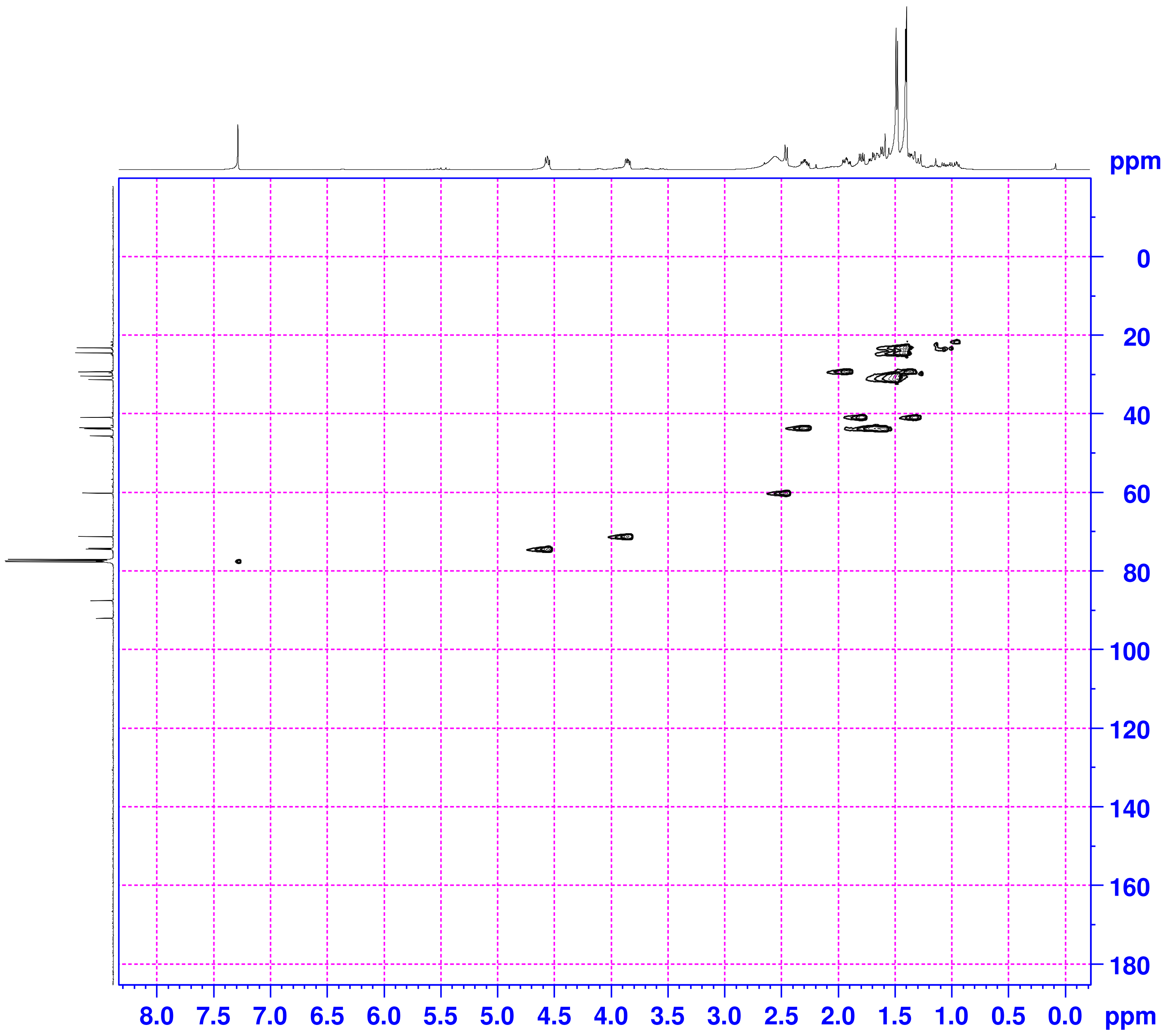


```
Current Data Parameters
NAME        HGSVcaro86-2D
EXPNO                  15
PROCNO                  1

F2 - Acquisition Parameters
Date_            20220523
Time                12.38 h
INSTRUM   Avance AV Neo 400
PROBHD    Z108618_0912 (
PULPROG        hsqcetgpsi2
TD                   1024
SOLVENT             CH3OH
NS                     32
DS                     16
SWH              3424.657 Hz
FIDRES           6.688784 Hz
AQ              0.1495040 sec
RG                    101
DW                146.000 usec
DE                   6.50 usec
TE                  298.1 K
CNST2         145.0000000
D0           0.00000300 sec
D1           1.50000000 sec
D4           0.00172414 sec
D11          0.03000000 sec
D16          0.00020000 sec
D24          0.00086207 sec
IN0          0.00002259 sec
TDav                    1
ZGOPTNS
SFO1          400.1316262 MHz
NUC1                   1H
P1                  14.00 usec
P2                  28.00 usec
PLW1          13.01399994 W
SFO2          100.6203145 MHz
NUC2                  13C
CPDPRG[2             garp
P3                  10.00 usec
P4                  20.00 usec
PCPD2               80.00 usec
PLW2          53.78799820 W
PLW12          0.84043998 W
GPNAM[1]       SMSQ10.100
GPZ1                80.00 %
GPNAM[2]       SMSQ10.100
GPZ2                20.10 %
GPNAM[3]       SMSQ10.100
GPZ3                11.00 %
GPNAM[4]       SMSQ10.100
GPZ4                -5.00 %
P16               1000.00 usec
P19                600.00 usec

======== F1 INDIRECT DIMENSION ========
td1       256
sw_F1     220.000000

F1 - Acquisition parameters
TD                    256
SFO1             100.6203 MHz
FIDRES         172.938568 Hz
SW                219.997 ppm
FnMODE       Echo-Antiecho

F2 - Processing parameters
SI                   1024
SF            400.1300038 MHz
WDW                 QSINE
SSB                     2
LB                      0 Hz
GB                      0
PC                   1.40

F1 - Processing parameters
SI                   1024
MC2          echo-antiecho
SF            100.6127390 MHz
WDW                 QSINE
SSB                     2
LB                      0 Hz
GB                      0
```

CM2

HSQC SPECTRUM DR.ORABI HGS V caro 86 IN CDCL3

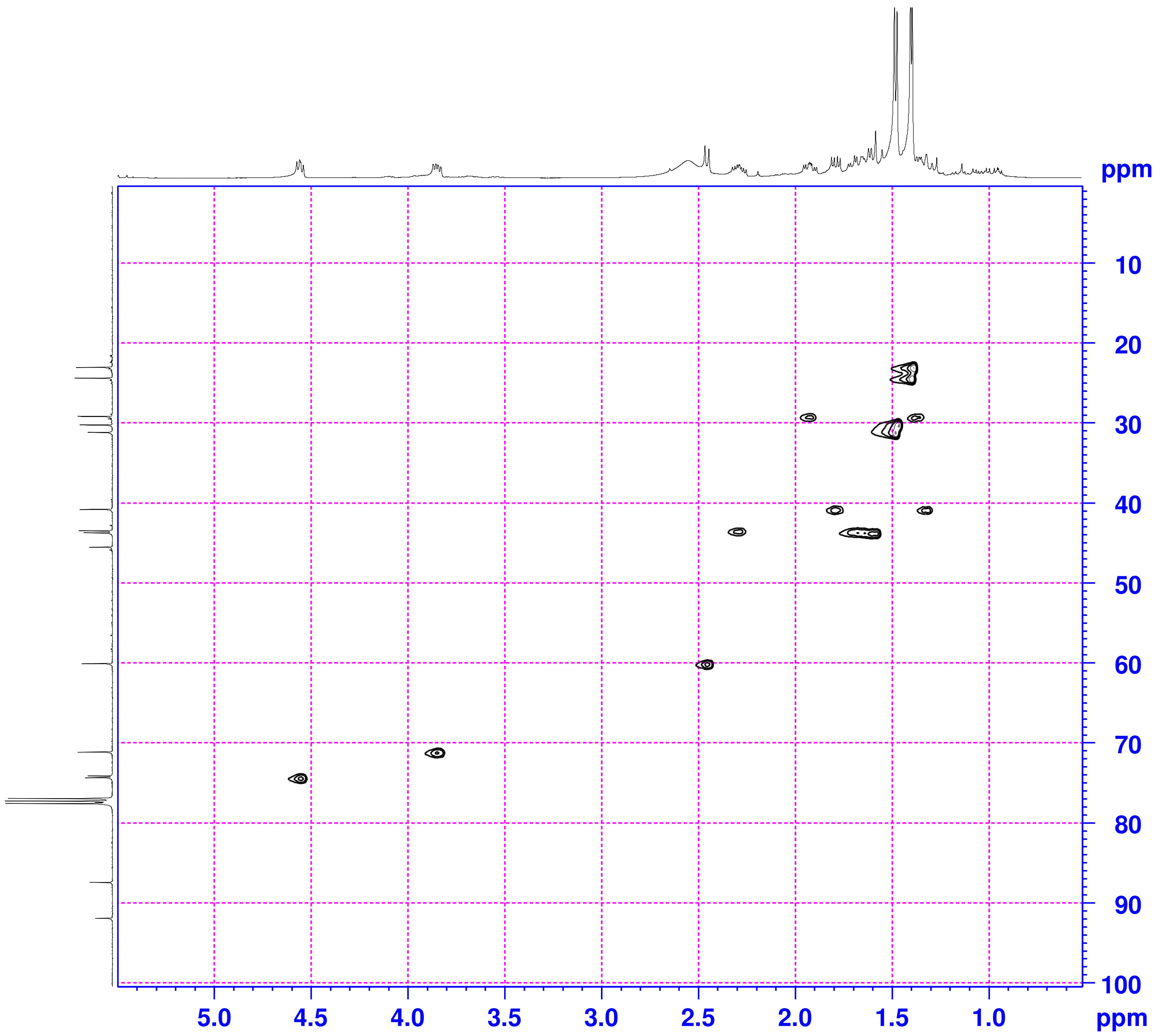


```
Current Data Parameters
NAME          HGSVcaro86-2D
EXPNO                    15
PROCNO                    1

F2 - Acquisition Parameters
Date_              20220523
Time                  12.38 h
INSTRUM    Avance AV Neo 400
PROBHD     Z108618_0912 (
PULPROG        hsqcetgpsi2
TD                     1024
SOLVENT               CH3OH
NS                       32
DS                       16
SWH                3424.657 Hz
FIDRES             6.688784 Hz
AQ                0.1495040 sec
RG                      101
DW                  146.000 usec
DE                     6.50 usec
TE                    298.1 K
CNST2           145.0000000
D0               0.00000300 sec
D1               1.50000000 sec
D4               0.00172414 sec
D11              0.03000000 sec
D16              0.00020000 sec
D24              0.00086207 sec
IN0              0.00002259 sec
TDav                      1
ZGOPTNS
SFO1            400.1316262 MHz
NUC1                     1H
P1                    14.00 usec
P2                    28.00 usec
PLW1            13.01399994 W
SFO2            100.6203145 MHz
NUC2                    13C
CPDPRG[2                garp
P3                    10.00 usec
P4                    20.00 usec
PCPD2                 80.00 usec
PLW2            53.78799820 W
PLW12            0.84043998 W
GPNAM[1]         SMSQ10.100
GPZ1                  80.00 %
GPNAM[2]         SMSQ10.100
GPZ2                  20.10 %
GPNAM[3]         SMSQ10.100
GPZ3                  11.00 %
GPNAM[4]         SMSQ10.100
GPZ4                  -5.00 %
P16                 1000.00 usec
P19                  600.00 usec

======== F1 INDIRECT DIMENSION ========
td1        256
sw_F1      220.000000

F1 - Acquisition parameters
TD                      256
SFO1               100.6203 MHz
FIDRES           172.938568 Hz
SW                  219.997 ppm
FnMODE        Echo-Antiecho

F2 - Processing parameters
SI                     1024
SF              400.1300038 MHz
WDW                   QSINE
SSB                       2
LB                        0 Hz
GB                        0
PC                     1.40

F1 - Processing parameters
SI                     1024
MC2            echo-antiecho
SF              100.6127390 MHz
WDW                   QSINE
SSB                       2
LB                        0 Hz
GB                        0
```

CM2

HMBC SPECTRUM DR.ORABI HGS V caro 86 IN CDCL3

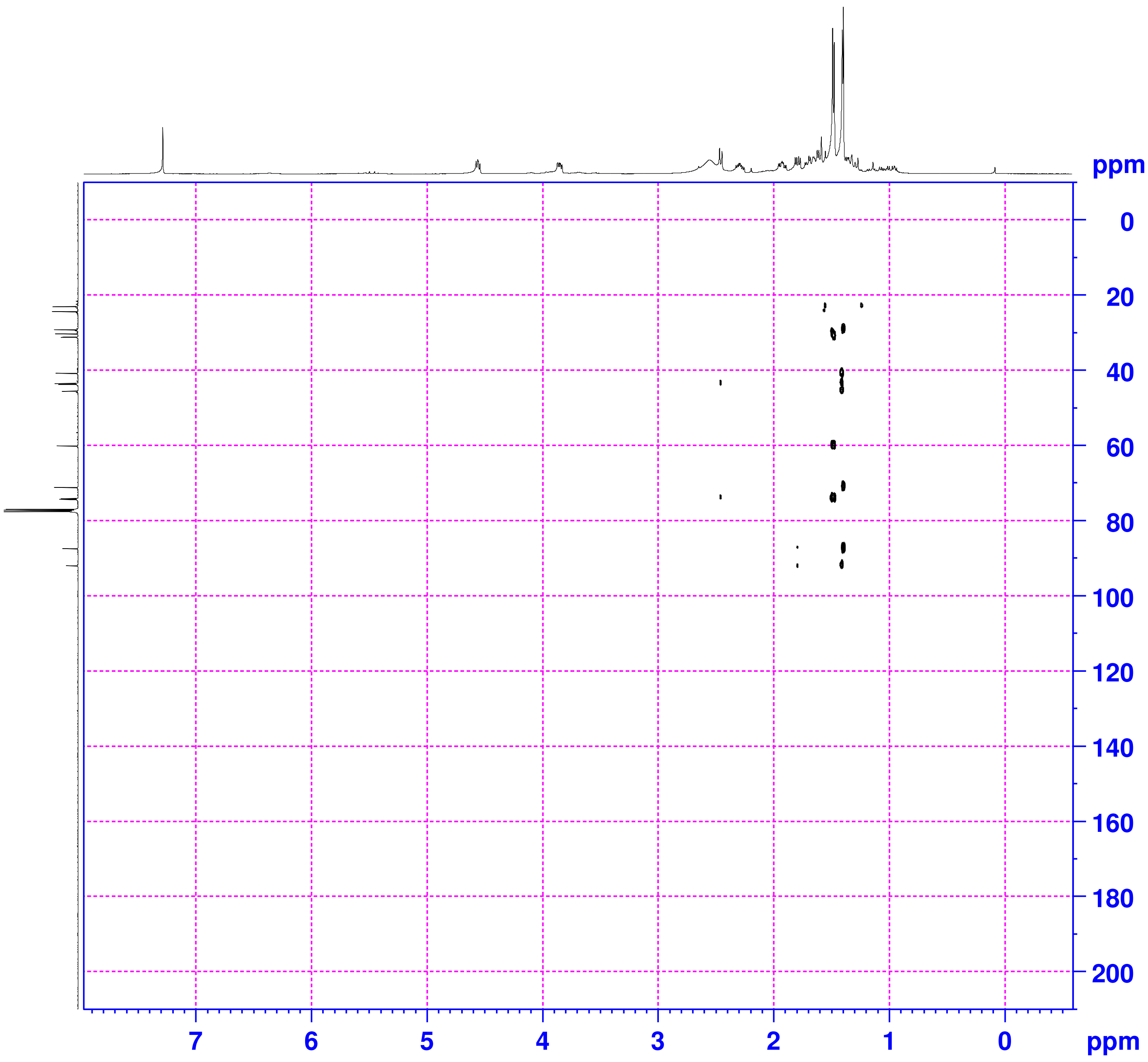


```
Current Data Parameters
NAME       HGSVcaro86-2D
EXPNO                 14
PROCNO                 1

F2 - Acquisition Parameters
Date_          20220523
Time               8.50 h
INSTRUM   Avance AV Neo 400
PROBHD    Z108618_0912 (
PULPROG     hmbcgplpndqf
TD                 4096
SOLVENT           CH3OH
NS                   32
DS                   16
SWH            3424.657 Hz
FIDRES         1.672196 Hz
AQ            0.5980160 sec
RG                  101
DW              146.000 usec
DE                 6.50 usec
TE                297.9 K
CNST2       145.0000000
CNST13       10.0000000
D0           0.00000300 sec
D1           1.50000000 sec
D2           0.00344828 sec
D6           0.05000000 sec
D16          0.00020000 sec
IN0          0.00002259 sec
TDav                  1
SFO1        400.1314729 MHz
NUC1                 1H
P1                14.00 usec
P2                28.00 usec
PLW1        13.01399994 W
SFO2        100.6228298 MHz
NUC2                13C
P3                10.00 usec
PLW2        53.78799820 W
GPNAM[1]      SMSQ10.100
GPZ1              50.00 %
GPNAM[2]      SMSQ10.100
GPZ2              30.00 %
GPNAM[3]      SMSQ10.100
GPZ3              40.10 %
P16             1000.00 usec

======== F1 INDIRECT DIMENSION ========
td1       128
sw_F1     220.000000

F1 - Acquisition parameters
TD                  128
SFO1           100.6228 MHz
FIDRES       345.877136 Hz
SW              219.991 ppm
FnMODE               QF

F2 - Processing parameters
SI                 4096
SF          400.1299966 MHz
WDW               QSINE
SSB                   0
LB                    0 Hz
GB                    0
PC                 1.40

F1 - Processing parameters
SI                 1024
MC2                  QF
SF          100.6127685 MHz
WDW               QSINE
SSB                   0
LB                    0 Hz
GB                    0
```

CM2

HMBC SPECTRUM DR.ORABI HGS V caro 86 IN CDCL3

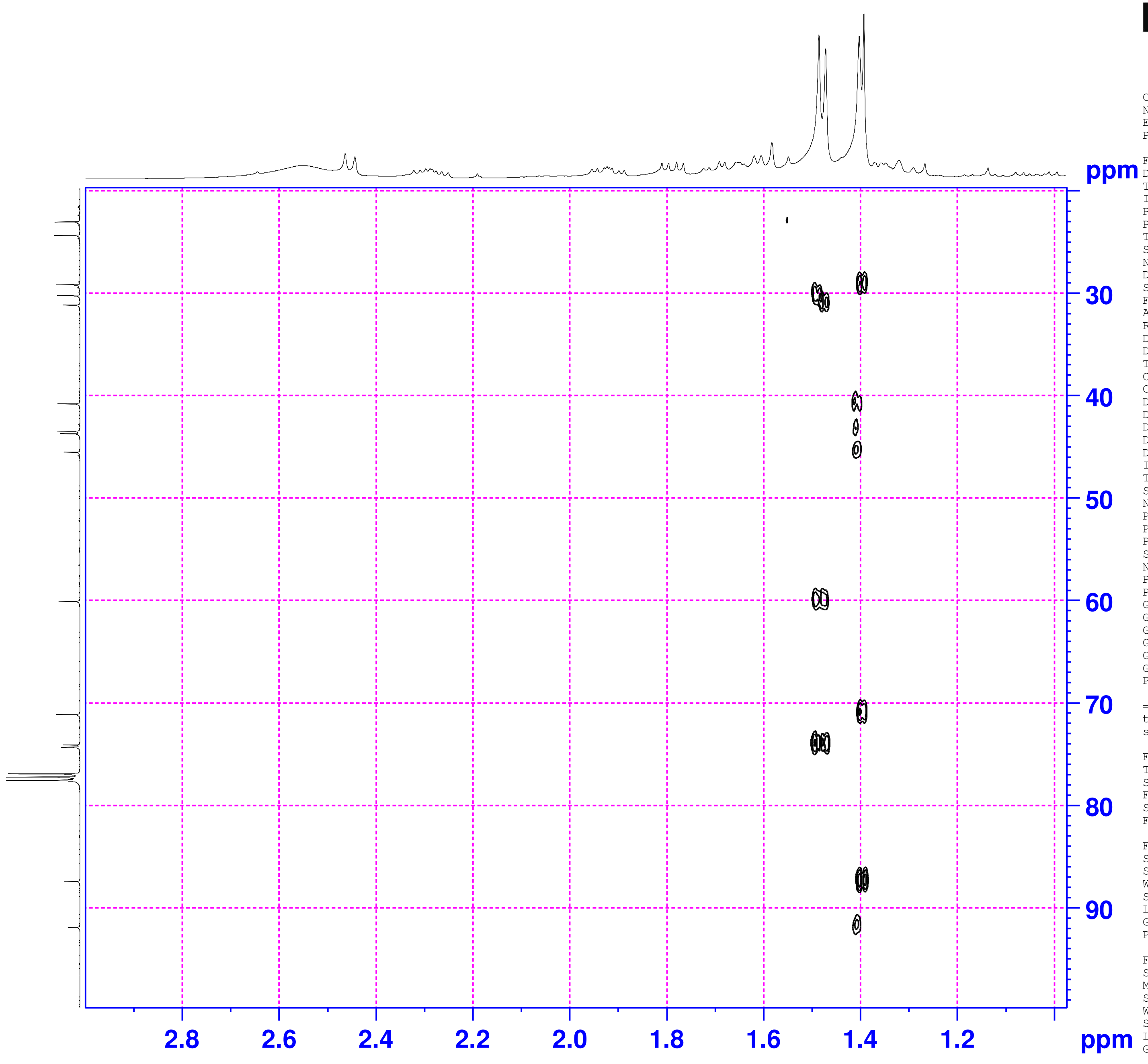

Current Data Parameters

NAME	HGSVcaro86-2D
EXPNO	14
PROCNO	1

F2 - Acquisition Parameters

Date_	20220523
Time	8.50 h
INSTRUM	Avance AV Neo 400
PROBHD	Z108618_0912 (
PULPROG	hmbcgplpndqf
TD	4096
SOLVENT	CH3OH
NS	32
DS	16
SWH	3424.657 Hz
FIDRES	1.672196 Hz
AQ	0.5980160 sec
RG	101
DW	146.000 usec
DE	6.50 usec
TE	297.9 K
CNST2	145.0000000
CNST13	10.0000000
D0	0.00000300 sec
D1	1.50000000 sec
D2	0.00344828 sec
D6	0.05000000 sec
D16	0.00020000 sec
IN0	0.00002259 sec
TDav	1
SFO1	400.1314729 MHz
NUC1	1H
P1	14.00 usec
P2	28.00 usec
PLW1	13.01399994 W
SFO2	100.6228298 MHz
NUC2	13C
P3	10.00 usec
PLW2	53.78799820 W
GPNAM[1]	SMSQ10.100
GPZ1	50.00 %
GPNAM[2]	SMSQ10.100
GPZ2	30.00 %
GPNAM[3]	SMSQ10.100
GPZ3	40.10 %
P16	1000.00 usec

======== F1 INDIRECT DIMENSION ========

td1	128
sw_F1	220.000000

F1 - Acquisition parameters

TD	128
SFO1	100.6228 MHz
FIDRES	345.877136 Hz
SW	219.991 ppm
FnMODE	QF

F2 - Processing parameters

SI	4096
SF	400.1299966 MHz
WDW	QSINE
SSB	0
LB	0 Hz
GB	0
PC	1.40

F1 - Processing parameters

SI	1024
MC2	QF
SF	100.6127685 MHz
WDW	QSINE
SSB	0
LB	0 Hz
GB	0

CM2

COSY SPECTRUM DR.ORABI HGS V caro 86 IN CDCL3

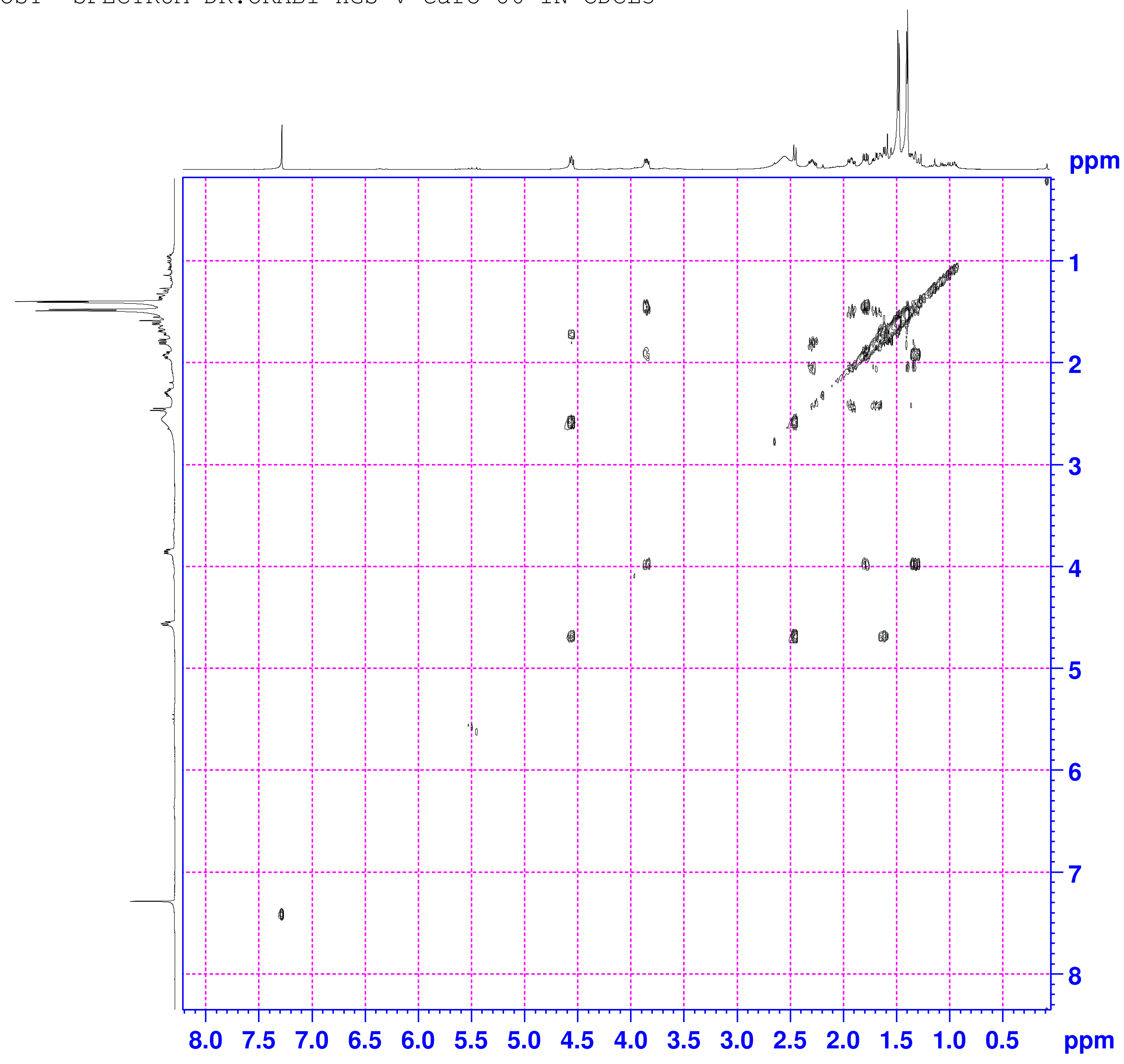


```
Current Data Parameters
NAME          HGSVcaro86-2D
EXPNO                    12
PROCNO                    1

F2 - Acquisition Parameters
Date_              20220523
Time                 6.00 h
INSTRUM   Avance AV Neo 400
PROBHD    Z108618_0912 (
PULPROG          cosygpppqf
TD                     2048
SOLVENT               CH3OH
NS                        8
DS                       16
SWH                3267.974 Hz
FIDRES             3.191381 Hz
AQ                0.3133440 sec
RG                      101
DW                  153.000 usec
DE                     6.50 usec
TE                    298.4 K
D0               0.00000300 sec
D1               1.88735998 sec
D11              0.03000000 sec
D12              0.00002000 sec
D13              0.00000400 sec
D16              0.00020000 sec
IN0              0.00030600 sec
TDav                      1
SFO1            400.1316515 MHz
NUC1                     1H
P0                    14.00 usec
P1                    14.00 usec
P17                 2500.00 usec
PLW1            13.01399994 W
PLW10            2.83430004 W
GPNAM[1]         SMSQ10.100
GPZ1                  10.00 %
P16                 1000.00 usec

======== F1 INDIRECT DIMENSION ========
td1        128
sw_F1      8.167247

F1 - Acquisition parameters
TD                      128
SFO1               400.1317 MHz
FIDRES            51.062092 Hz
SW                    8.167 ppm
FnMODE                   QF

F2 - Processing parameters
SI                     1024
SF              400.1299976 MHz
WDW                   QSINE
SSB                       0
LB                        0 Hz
GB                        0
PC                     1.40

F1 - Processing parameters
SI                     1024
MC2                      QF
SF              400.1299447 MHz
WDW                   QSINE
SSB                       0
LB                        0 Hz
GB                        0
```

CM2

COSY SPECTRUM DR.ORABI HGS V caro 86 IN CDCL3

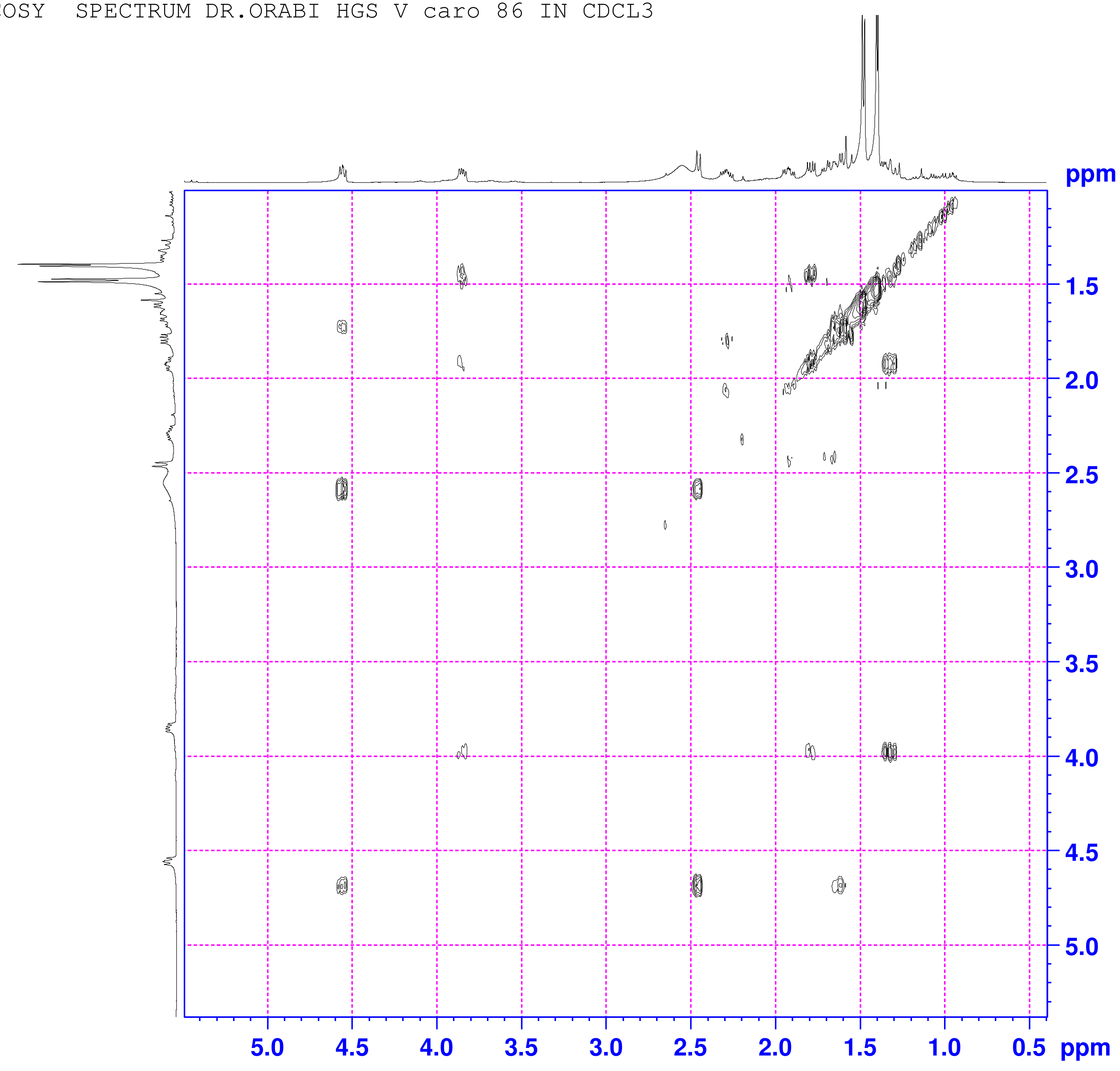


```
Current Data Parameters
NAME        HGSVcaro86-2D
EXPNO                  12
PROCNO                  1

F2 - Acquisition Parameters
Date_            20220523
Time                 6.00 h
INSTRUM   Avance AV Neo 400
PROBHD    Z108618_0912 (
PULPROG        cosygpppqf
TD                   2048
SOLVENT             CH3OH
NS                      8
DS                     16
SWH              3267.974 Hz
FIDRES           3.191381 Hz
AQ              0.3133440 sec
RG                    101
DW                153.000 usec
DE                   6.50 usec
TE                  298.4 K
D0             0.00000300 sec
D1             1.88735998 sec
D11            0.03000000 sec
D12            0.00002000 sec
D13            0.00000400 sec
D16            0.00020000 sec
IN0            0.00030600 sec
TDav                    1
SFO1          400.1316515 MHz
NUC1                   1H
P0                  14.00 usec
P1                  14.00 usec
P17               2500.00 usec
PLW1          13.01399994 W
PLW10          2.83430004 W
GPNAM[1]       SMSQ10.100
GPZ1                10.00 %
P16               1000.00 usec

======== F1 INDIRECT DIMENSION ========
td1         128
sw_F1       8.167247

F1 - Acquisition parameters
TD                    128
SFO1             400.1317 MHz
FIDRES          51.062092 Hz
SW                  8.167 ppm
FnMODE                 QF

F2 - Processing parameters
SI                   1024
SF            400.1299976 MHz
WDW                 QSINE
SSB                     0
LB                      0 Hz
GB                      0
PC                   1.40

F1 - Processing parameters
SI                   1024
MC2                    QF
SF            400.1299447 MHz
WDW                 QSINE
SSB                     0
LB                      0 Hz
GB                      0
```

CM3

1H spectra Dr.Orabi Caro M3 in CDCL3

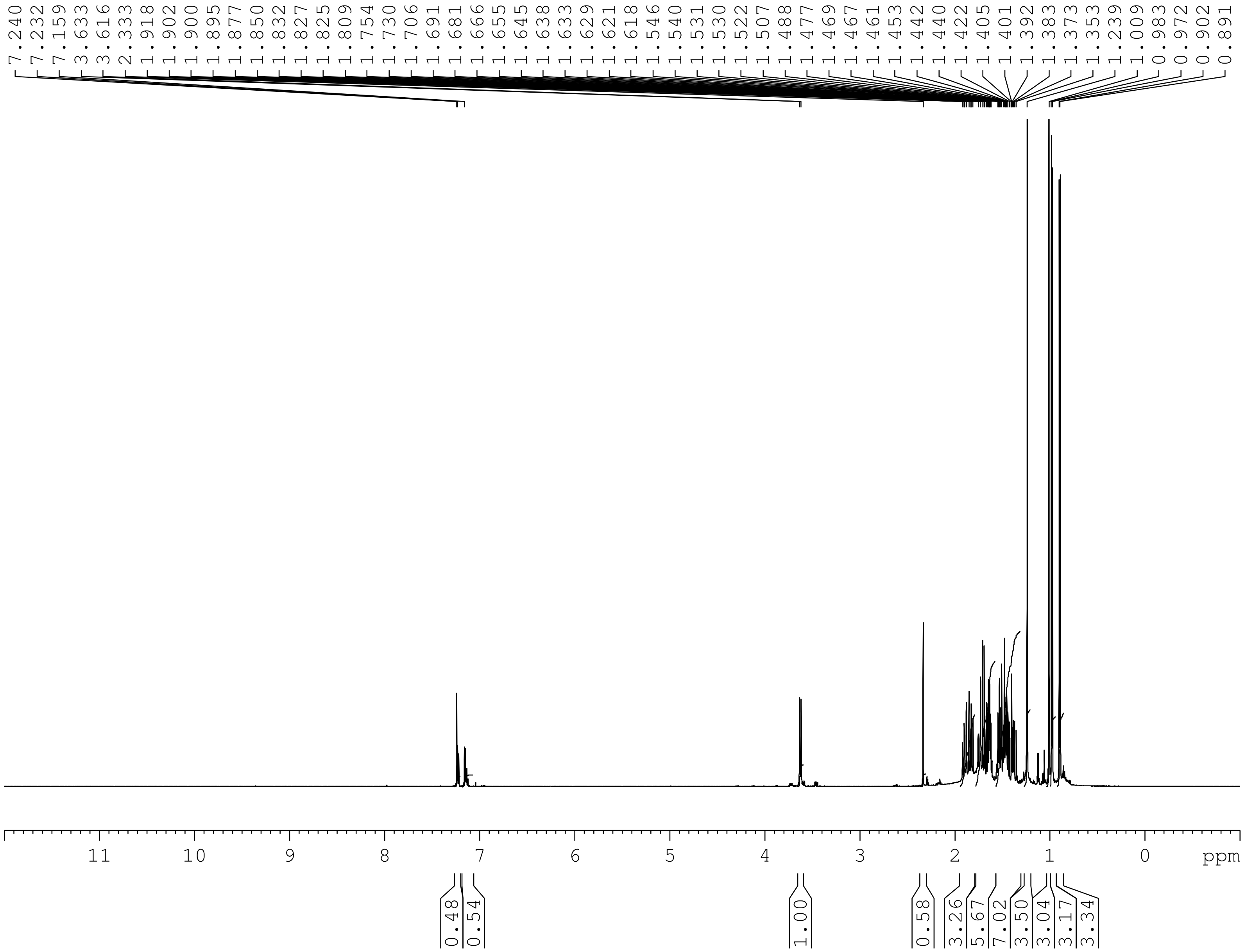


```
Current Data Parameters
NAME             CaroM3-2D
EXPNO                    2
PROCNO                   1

F2 - Acquisition Parameter
Date_             20220630
Time                 14.04
INSTRUM              spect
PROBHD   5 mm PABBO BB-
PULPROG               zg30
TD                   65536
SOLVENT              CDCl3
NS                      16
DS                       2
SWH            12335.526 Hz
FIDRES          0.188225 Hz
AQ             2.6563926 se
RG                     203
DW                  40.533 us
DE                   20.00 us
TE                   298.0 K
D1            1.00000000 se
TD0                      1

======== CHANNEL f1 ======
SFO1         600.1337060 MH
NUC1                    1H
P1                   10.60 us
PLW1           27.82500076 W

F2 - Processing parameters
SI                   32768
SF           600.1300280 MH
WDW                     EM
SSB        0
LB                    0.30 Hz
GB         0
PC                    1.00
```

CM3

1H spectra Dr.Orabi Caro M3 in CDCL3

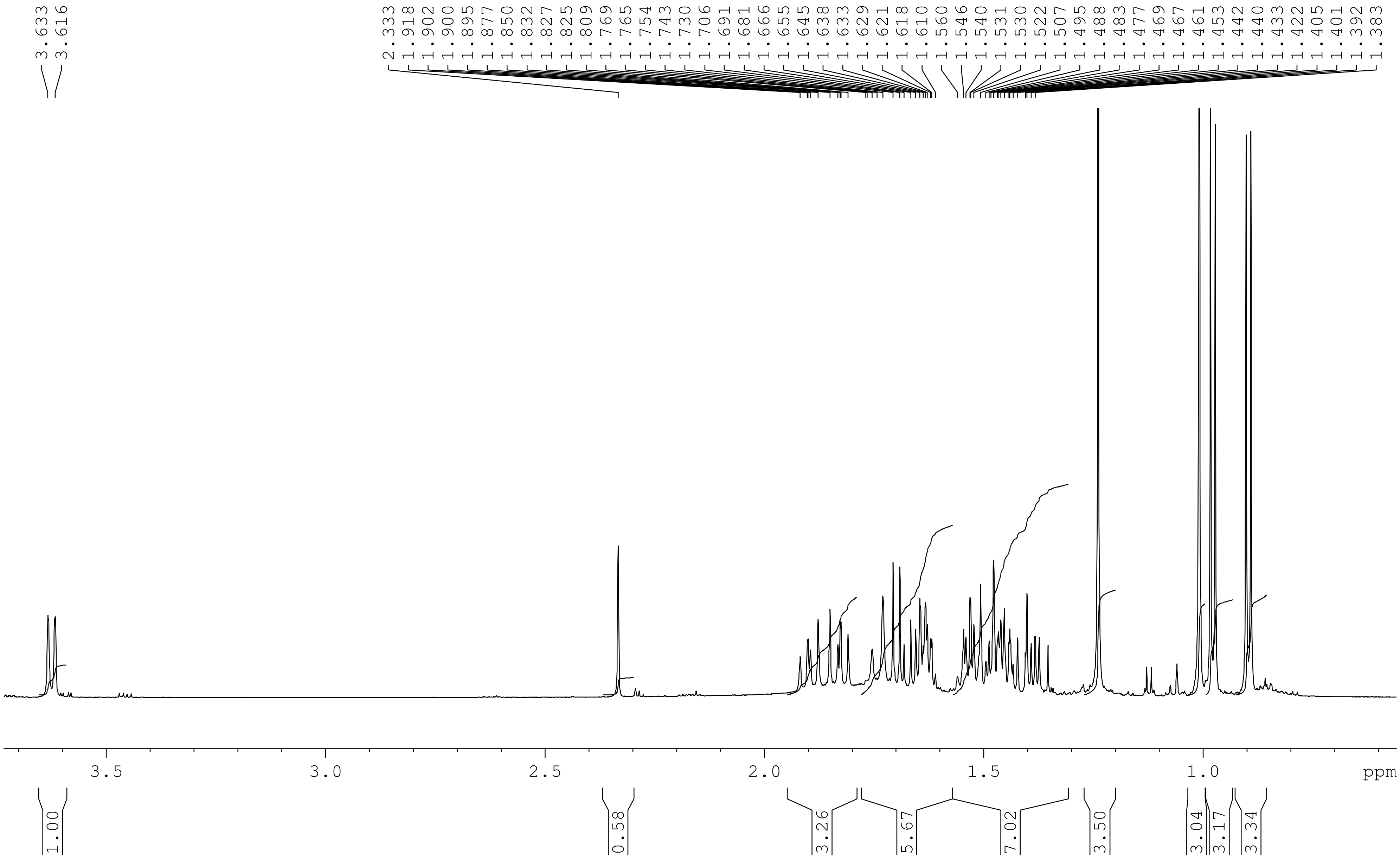

CM3

13C decoupled spectra Dr.Orabi Caro M3 in CDCL3

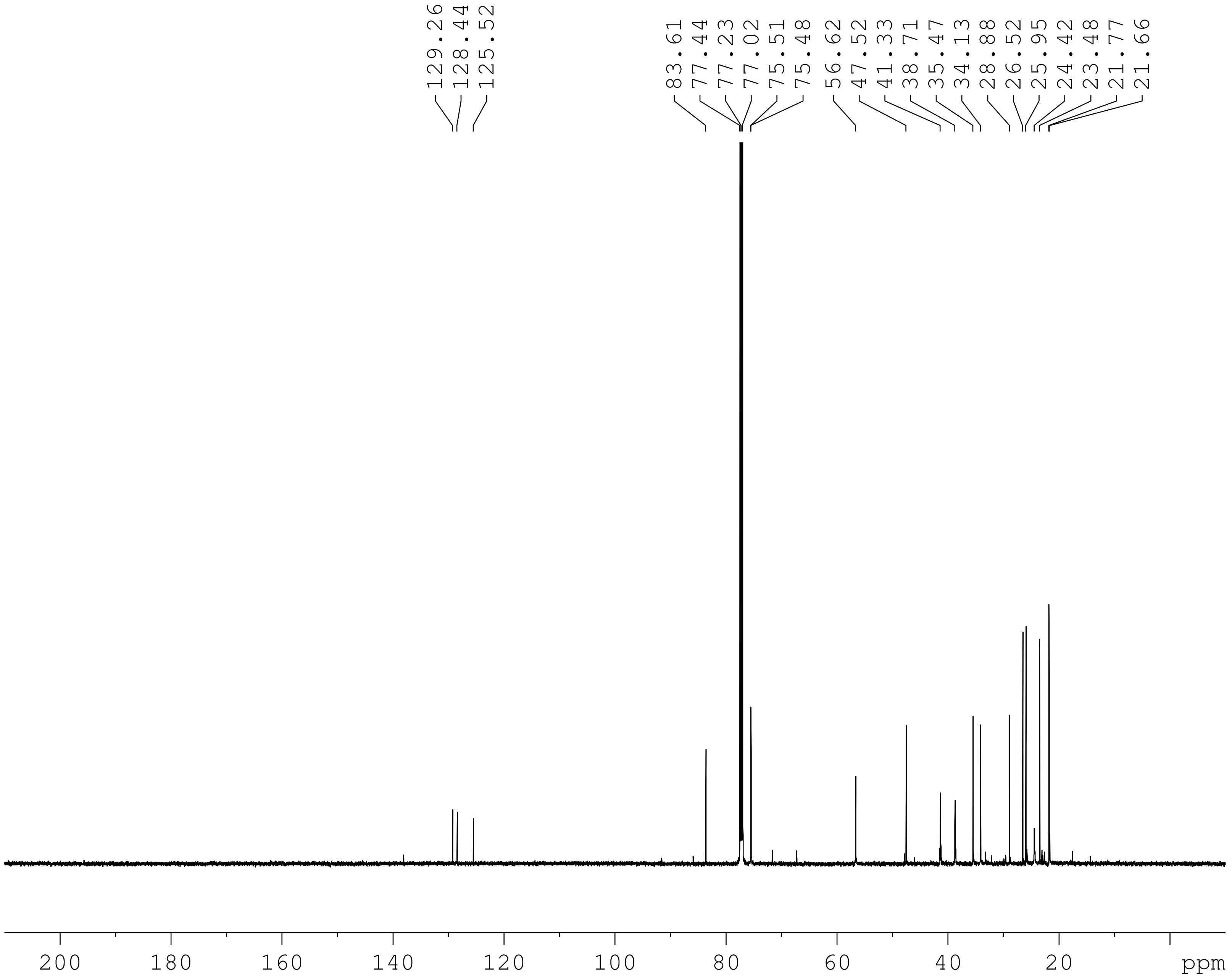

Current Data Parameters
NAME CaroM3-2D
EXPNO 3
PROCNO 1

F2 - Acquisition Parameters
Date_ 20220630
Time 22.34
INSTRUM spect
PROBHD 5 mm PABBO BB-
PULPROG zgpg30
TD 65536
SOLVENT CDCl3
NS 10240
DS 4
SWH 36057.691 Hz
FIDRES 0.550197 Hz
AQ 0.9087659 sec
RG 203
DW 13.867 usec
DE 50.00 usec
TE 300.7 K
D1 2.00000000 sec
D11 0.03000000 sec
TD0 1

======== CHANNEL f1 ========
SFO1 150.9178979 MHz
NUC1 13C
P1 8.80 usec
PLW1 78.13500214 W

======== CHANNEL f2 ========
SFO2 600.1324005 MHz
NUC2 1H
CPDPRG[2 waltz65
PCPD2 70.00 usec
PLW2 27.82500076 W
PLW12 0.63804001 W
PLW13 0.31264001 W

F2 - Processing parameters
SI 32768
SF 150.9027757 MHz
WDW EM
SSB 0
LB 1.00 Hz
GB 0
PC 1.40

CM3

13C decoupled spectra Dr.Orabi Caro M3 in CDCL3

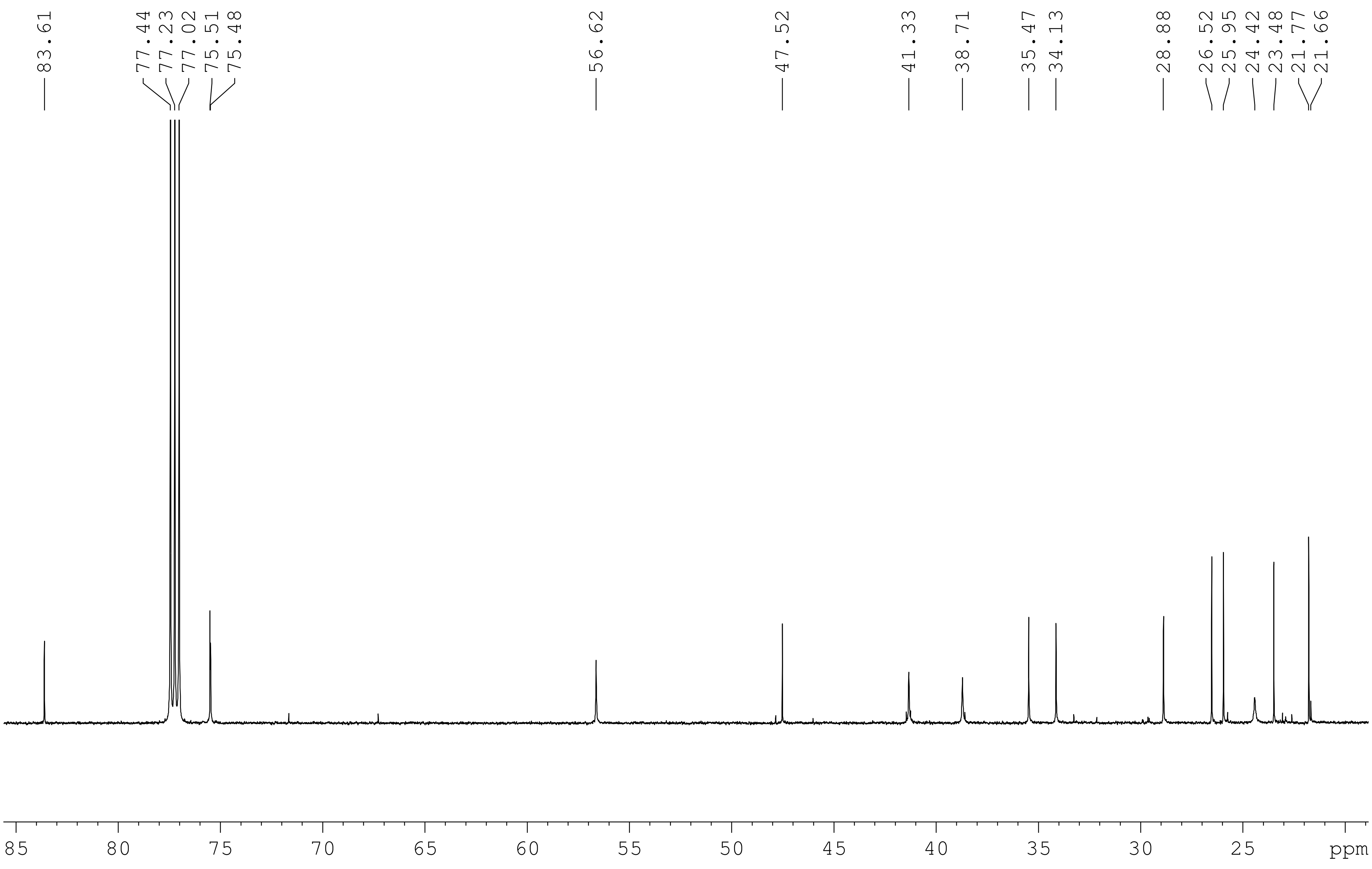

CM3

DEPT 135 spectra Dr.Orabi Caro M3 in CDCL3

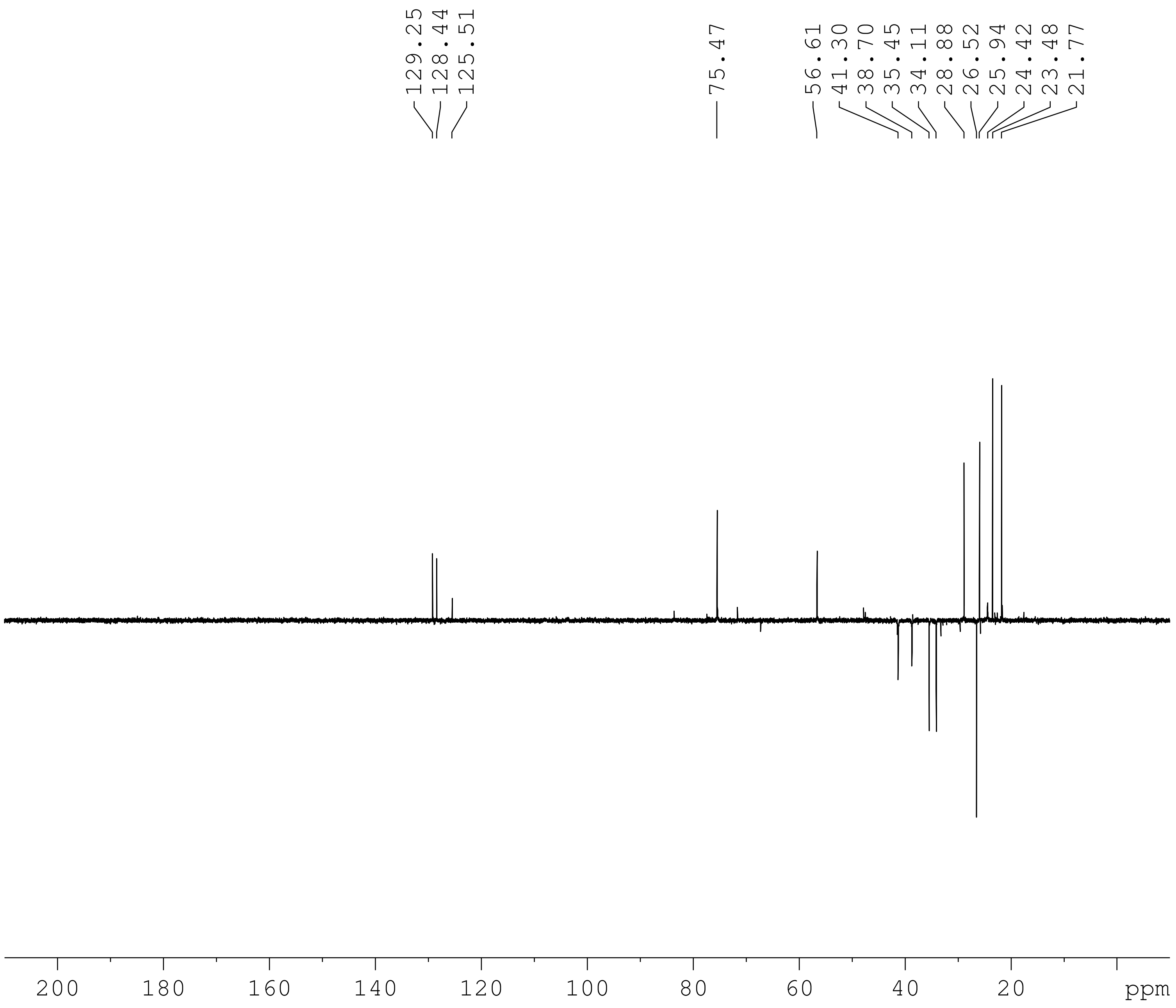

Current Data Parameters	
NAME	CaroM3-2D
EXPNO	6
PROCNO	1
F2 - Acquisition Parameters	
Date_	20220701
Time	8.48
INSTRUM	spect
PROBHD	5 mm PABBO BB-
PULPROG	deptsp135
TD	65536
SOLVENT	CDCl3
NS	4096
DS	4
SWH	36057.691 Hz
FIDRES	0.550197 Hz
AQ	0.9087659 sec
RG	203
DW	13.867 usec
DE	50.00 usec
TE	299.4 K
CNST2	145.0000000
D1	2.00000000 sec
D2	0.00344828 sec
D12	0.00002000 sec
TD0	1
======== CHANNEL f1 ========	
SFO1	150.9178979 MHz
NUC1	13C
P1	8.80 usec
P13	2000.00 usec
PLW0	0 W
PLW1	78.13500214 W
SPNAM[5]	Crp60comp.4
SPOAL5	0.500
SPOFFS5	0 Hz
SPW5	9.24489975 W
======== CHANNEL f2 ========	
SFO2	600.1324005 MHz
NUC2	1H
CPDPRG[2	waltz65
P3	10.60 usec
P4	21.20 usec
PCPD2	70.00 usec
PLW2	27.82500076 W
PLW12	0.63804001 W
F2 - Processing parameters	
SI	32768
SF	150.9027774 MHz
WDW	EM
SSB	0
LB	1.00 Hz
GB	0
PC	1.40

CM3

DEPT 135 spectra Dr.Orabi Caro M3 in CDCL3

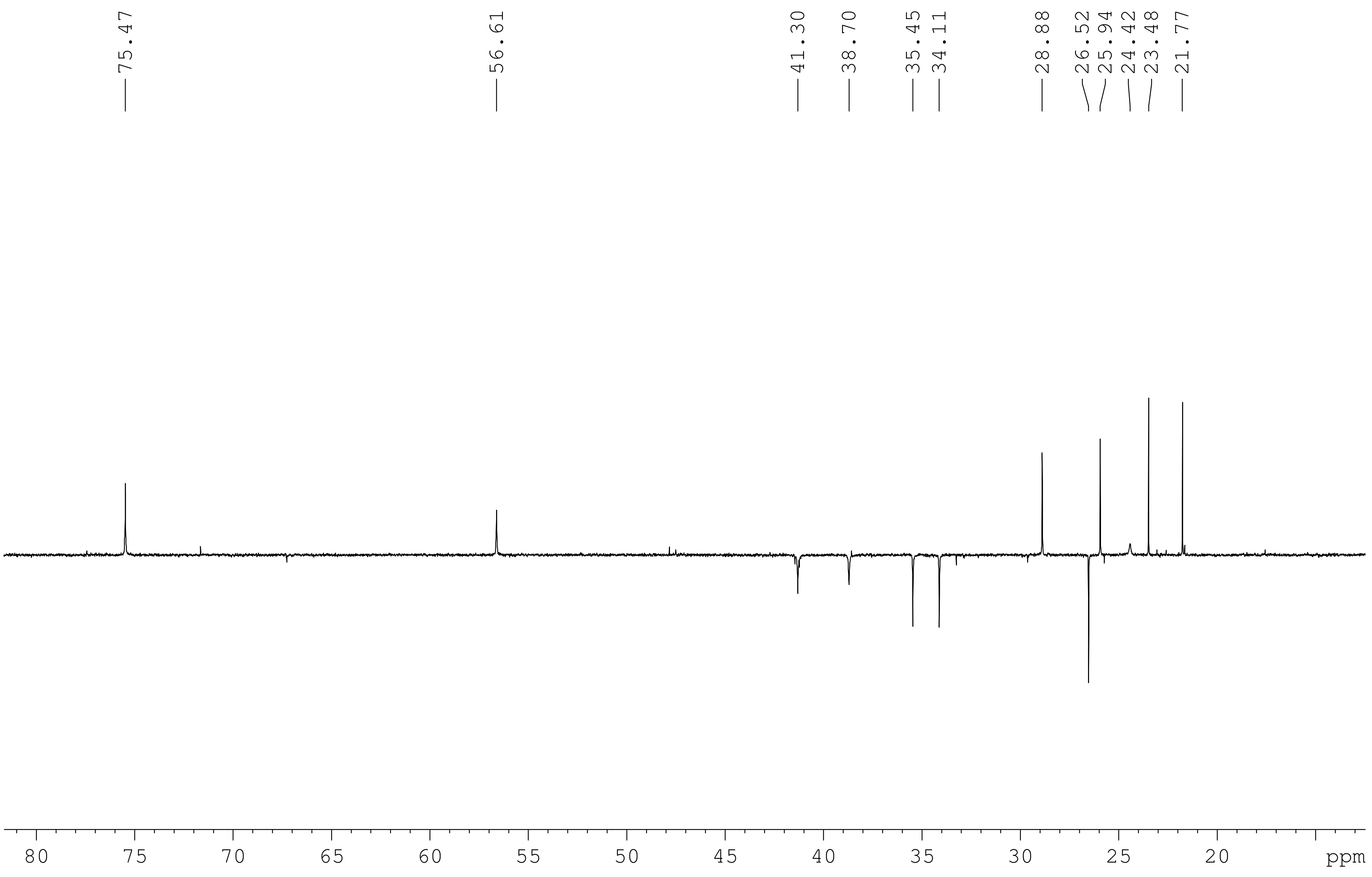

CM3

13C HSQC spectra Dr.Orabi Caro M3 in CDCL3

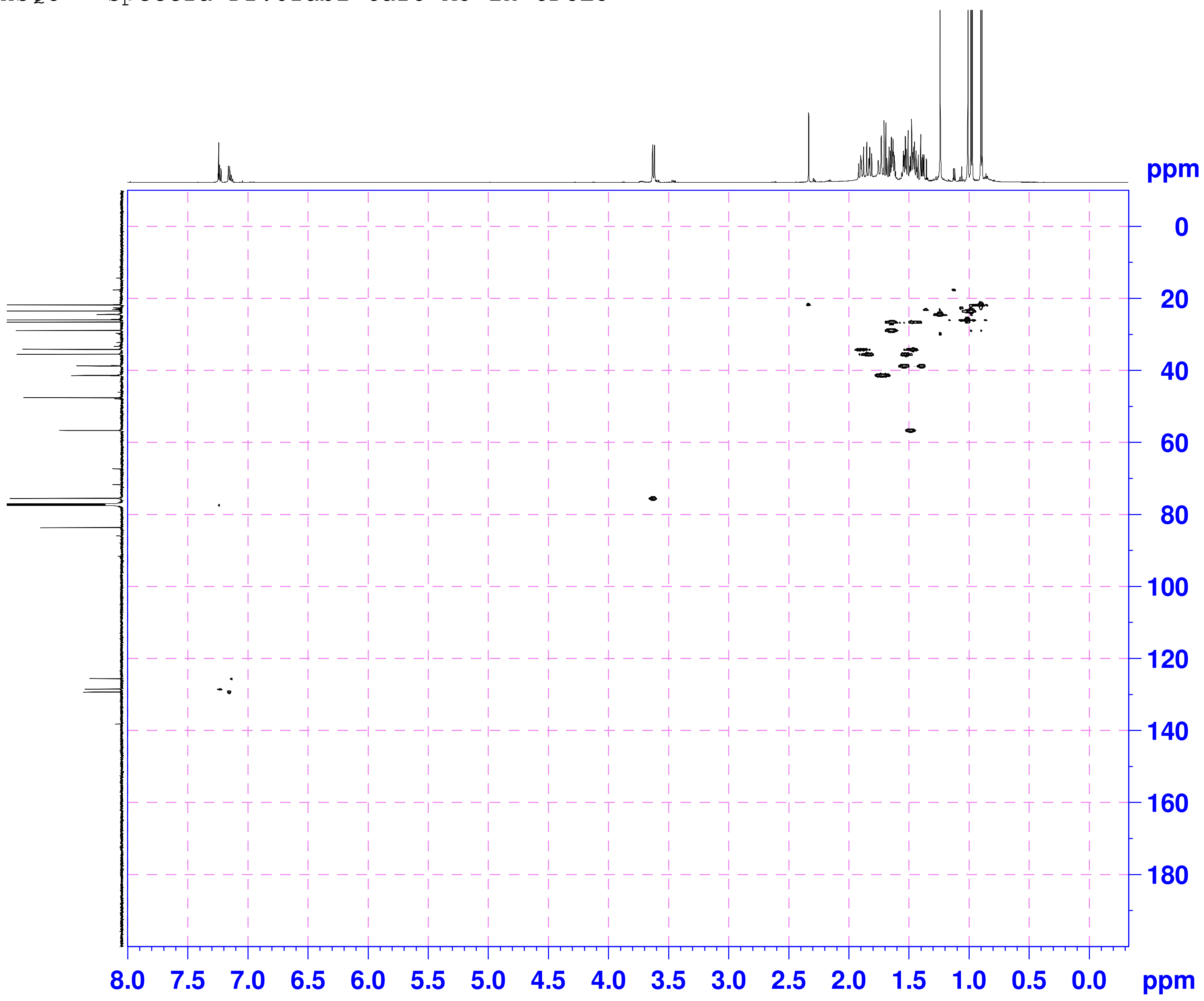


```
Current Data Parameters
NAME          CaroM3-2D
EXPNO                 8
PROCNO                1

F2 - Acquisition Parameters
Date_          20220701
Time               9.35
INSTRUM           spect
PROBHD   5 mm PABBO BB-
PULPROG    hsqcetgpsp.2
TD                 2048
SOLVENT           CDCl3
NS                   16
DS                   16
SWH            5000.000 Hz
FIDRES         2.441406 Hz
AQ            0.2048000 sec
RG                  203
DW              100.000 usec
DE                20.00 usec
TE                299.1 K
CNST2       145.0000000
D0           0.00000300 sec
D1           1.90169597 sec
D4           0.00172414 sec
D11          0.03000000 sec
D16          0.00020000 sec
IN0          0.00001840 sec
ZGOPTNS

======== CHANNEL f1 ========
SFO1        600.1323288 MHz
NUC1                 1H
P1                10.60 usec
P2                21.20 usec
P28             1000.00 usec
PLW1        27.82500076 W

======== CHANNEL f2 ========
SFO2        150.9178993 MHz
NUC2                13C
CPDPRG[2          garp4
P3                 8.80 usec
P14              500.00 usec
P24             2000.00 usec
PCPD2             60.00 usec
PLW0      0 W
PLW2        78.13500214 W
PLW12        1.68079996 W
SPNAM[3] Crp60,0.5,20.1
SPOAL3            0.500
SPOFFS3   0 Hz
SPW3         9.24489975 W
SPNAM[7]    Crp60comp.4
SPOAL7            0.500
SPOFFS7   0 Hz
SPW7         9.24489975 W

====== GRADIENT CHANNEL =====
GPNAM[1]       SINE.100
GPNAM[2]       SINE.100
GPZ1              80.00 %
GPZ2              20.10 %
P16             1000.00 usec

F1 - Acquisition parameters
TD                  512
SFO1           150.9179 MHz
FIDRES        53.074047 Hz
SW              180.058 ppm
FnMODE     Echo-Antiecho

F2 - Processing parameters
SI                 2048
SF          600.1300268 MHz
WDW               QSINE
SSB                   2
LB        0 Hz
GB        0
PC                 1.40

F1 - Processing parameters
SI                 1024
MC2        echo-antiecho
SF          150.9027635 MHz
WDW               QSINE
SSB                   2
LB        0 Hz
GB        0
```

CM3

13C HSQC spectra Dr.Orabi Caro M3 in CDCL3

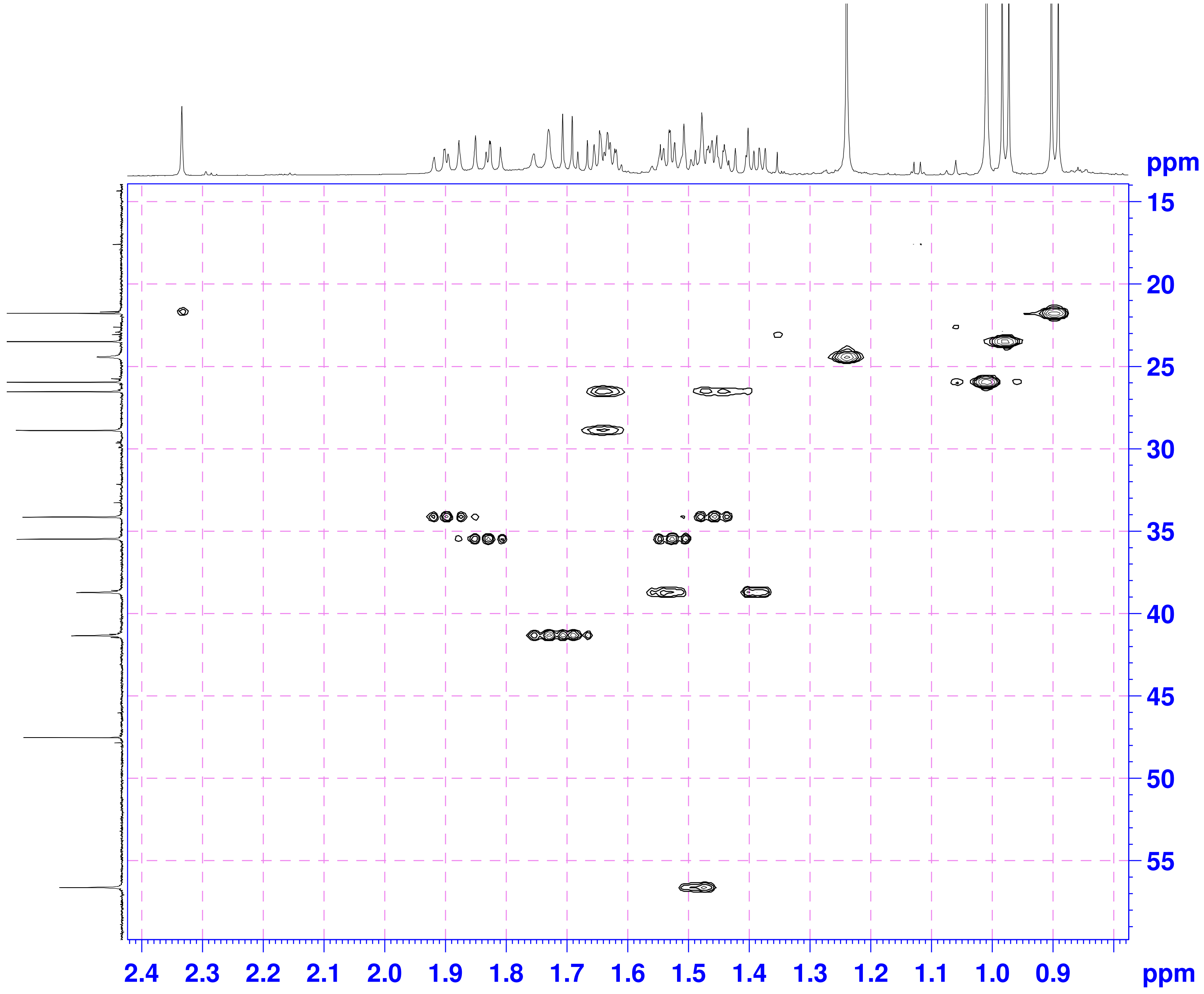

Current Data Parameters	
NAME	CaroM3-2D
EXPNO	8
PROCNO	1
F2 - Acquisition Parameters	
Date_	20220701
Time	9.35
INSTRUM	spect
PROBHD	5 mm PABBO BB-
PULPROG	hsqcetgpsp.2
TD	2048
SOLVENT	CDCl3
NS	16
DS	16
SWH	5000.000 Hz
FIDRES	2.441406 Hz
AQ	0.2048000 sec
RG	203
DW	100.000 usec
DE	20.00 usec
TE	299.1 K
CNST2	145.0000000
D0	0.00000300 sec
D1	1.90169597 sec
D4	0.00172414 sec
D11	0.03000000 sec
D16	0.00020000 sec
IN0	0.00001840 sec
ZGOPTNS	
======== CHANNEL f1 ========	
SFO1	600.1323288 MHz
NUC1	1H
P1	10.60 usec
P2	21.20 usec
P28	1000.00 usec
PLW1	27.82500076 W
======== CHANNEL f2 ========	
SFO2	150.9178993 MHz
NUC2	13C
CPDPRG[2	garp4
P3	8.80 usec
P14	500.00 usec
P24	2000.00 usec
PCPD2	60.00 usec
PLW0	0 W
PLW2	78.13500214 W
PLW12	1.68079996 W
SPNAM[3]	Crp60,0.5,20.1
SPOAL3	0.500
SPOFFS3	0 Hz
SPW3	9.24489975 W
SPNAM[7]	Crp60comp.4
SPOAL7	0.500
SPOFFS7	0 Hz
SPW7	9.24489975 W
====== GRADIENT CHANNEL =====	
GPNAM[1]	SINE.100
GPNAM[2]	SINE.100
GPZ1	80.00 %
GPZ2	20.10 %
P16	1000.00 usec
F1 - Acquisition parameters	
TD	512
SFO1	150.9179 MHz
FIDRES	53.074047 Hz
SW	180.058 ppm
FnMODE	Echo-Antiecho
F2 - Processing parameters	
SI	2048
SF	600.1300268 MHz
WDW	QSINE
SSB	2
LB	0 Hz
GB	0
PC	1.40
F1 - Processing parameters	
SI	1024
MC2	echo-antiecho
SF	150.9027635 MHz
WDW	QSINE
SSB	2
LB	0 Hz
GB	0

CM3

13C HSQC   spectra Dr.Orabi Caro M3 in CDCL3

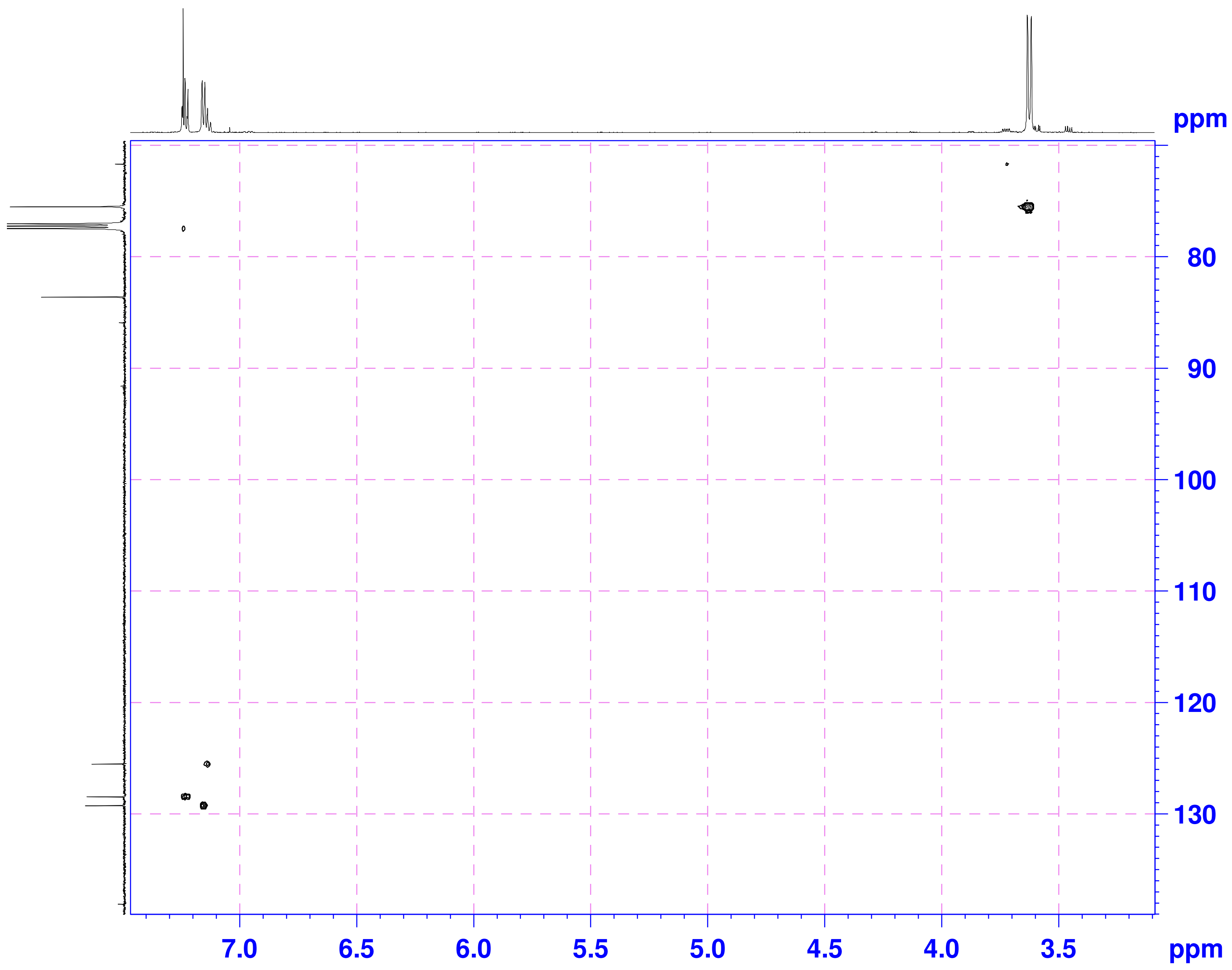


```
Current Data Parameters
NAME          CaroM3-2D
EXPNO                 8
PROCNO                1

F2 - Acquisition Parameters
Date_          20220701
Time               9.35
INSTRUM           spect
PROBHD   5 mm PABBO BB-
PULPROG    hsqcetgpsp.2
TD                 2048
SOLVENT           CDCl3
NS                   16
DS                   16
SWH            5000.000 Hz
FIDRES         2.441406 Hz
AQ            0.2048000 sec
RG                  203
DW              100.000 usec
DE                20.00 usec
TE                299.1 K
CNST2       145.0000000
D0           0.00000300 sec
D1           1.90169597 sec
D4           0.00172414 sec
D11          0.03000000 sec
D16          0.00020000 sec
IN0          0.00001840 sec
ZGOPTNS

======== CHANNEL f1 ========
SFO1        600.1323288 MHz
NUC1                 1H
P1                10.60 usec
P2                21.20 usec
P28             1000.00 usec
PLW1        27.82500076 W

======== CHANNEL f2 ========
SFO2        150.9178993 MHz
NUC2                13C
CPDPRG[2          garp4
P3                 8.80 usec
P14              500.00 usec
P24             2000.00 usec
PCPD2             60.00 usec
PLW0      0 W
PLW2        78.13500214 W
PLW12        1.68079996 W
SPNAM[3] Crp60,0.5,20.1
SPOAL3            0.500
SPOFFS3   0 Hz
SPW3         9.24489975 W
SPNAM[7]    Crp60comp.4
SPOAL7            0.500
SPOFFS7   0 Hz
SPW7         9.24489975 W

====== GRADIENT CHANNEL =====
GPNAM[1]       SINE.100
GPNAM[2]       SINE.100
GPZ1              80.00 %
GPZ2              20.10 %
P16             1000.00 usec

F1 - Acquisition parameters
TD                  512
SFO1           150.9179 MHz
FIDRES        53.074047 Hz
SW              180.058 ppm
FnMODE     Echo-Antiecho

F2 - Processing parameters
SI                 2048
SF          600.1300268 MHz
WDW               QSINE
SSB                   2
LB        0 Hz
GB        0
PC                 1.40

F1 - Processing parameters
SI                 1024
MC2        echo-antiecho
SF          150.9027635 MHz
WDW               QSINE
SSB                   2
LB        0 Hz
GB        0
```

CM3

13C HMBC spectra Dr.Orabi Caro M3 in CDCL3

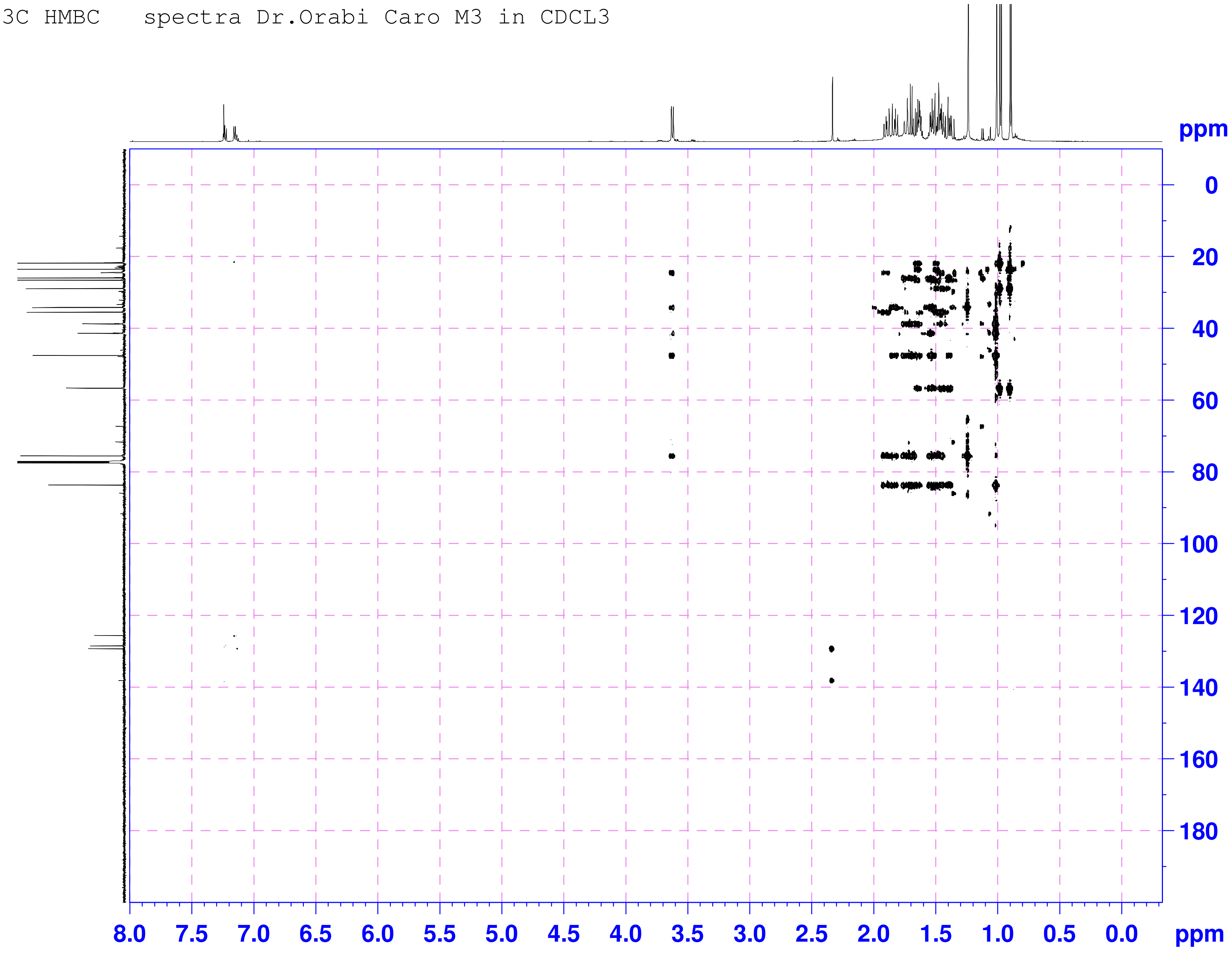

Current Data Parameters
NAME CaroM3-2D
EXPNO 9
PROCNO 1

F2 - Acquisition Parameters
Date_ 20220701
Time 14.29
INSTRUM spect
PROBHD 5 mm PABBO BB-
PULPROG hmbcetgpl2nd
TD 2048
SOLVENT CDCl3
NS 32
DS 16
SWH 5000.000 Hz
FIDRES 2.441406 Hz
AQ 0.2048000 sec
RG 203
DW 100.000 usec
DE 20.00 usec
TE 298.8 K
CNST6 125.0000000
CNST7 165.0000000
CNST13 10.0000000
CNST30 0.5981151
D0 0.00000300 sec
D1 1.42627203 sec
D6 0.05000000 sec
D16 0.00020000 sec
IN0 0.00001490 sec

======== CHANNEL f1 ========
SFO1 600.1323288 MHz
NUC1 1H
P1 10.60 usec
P2 21.20 usec
PLW1 27.82500076 W

======== CHANNEL f2 ========
SFO2 150.9178738 MHz
NUC2 13C
P3 8.80 usec
P24 2000.00 usec
PLW2 78.13500214 W
SPNAM[7] Crp60comp.4
SPOAL7 0.500
SPOFFS7 0 Hz
SPW7 9.24489975 W

====== GRADIENT CHANNEL =====
GPNAM[1] SINE.100
GPNAM[3] SINE.100
GPNAM[4] SINE.100
GPNAM[5] SINE.100
GPZ1 80.00 %
GPZ3 15.00 %
GPZ4 -10.00 %
GPZ5 -5.00 %
P16 1000.00 usec

F1 - Acquisition parameters
TD 256
SFO1 150.9179 MHz
FIDRES 131.082214 Hz
SW 222.353 ppm
FnMODE Echo-Antiecho

F2 - Processing parameters
SI 2048
SF 600.1300257 MHz
WDW SINE
SSB 0
LB 0 Hz
GB 0
PC 1.40

F1 - Processing parameters
SI 512
MC2 echo-antiecho
SF 150.9027314 MHz
WDW SINE
SSB 2
LB 0 Hz
GB 0

CM3

13C HMBC   spectra Dr.Orabi Caro M3 in CDCL3

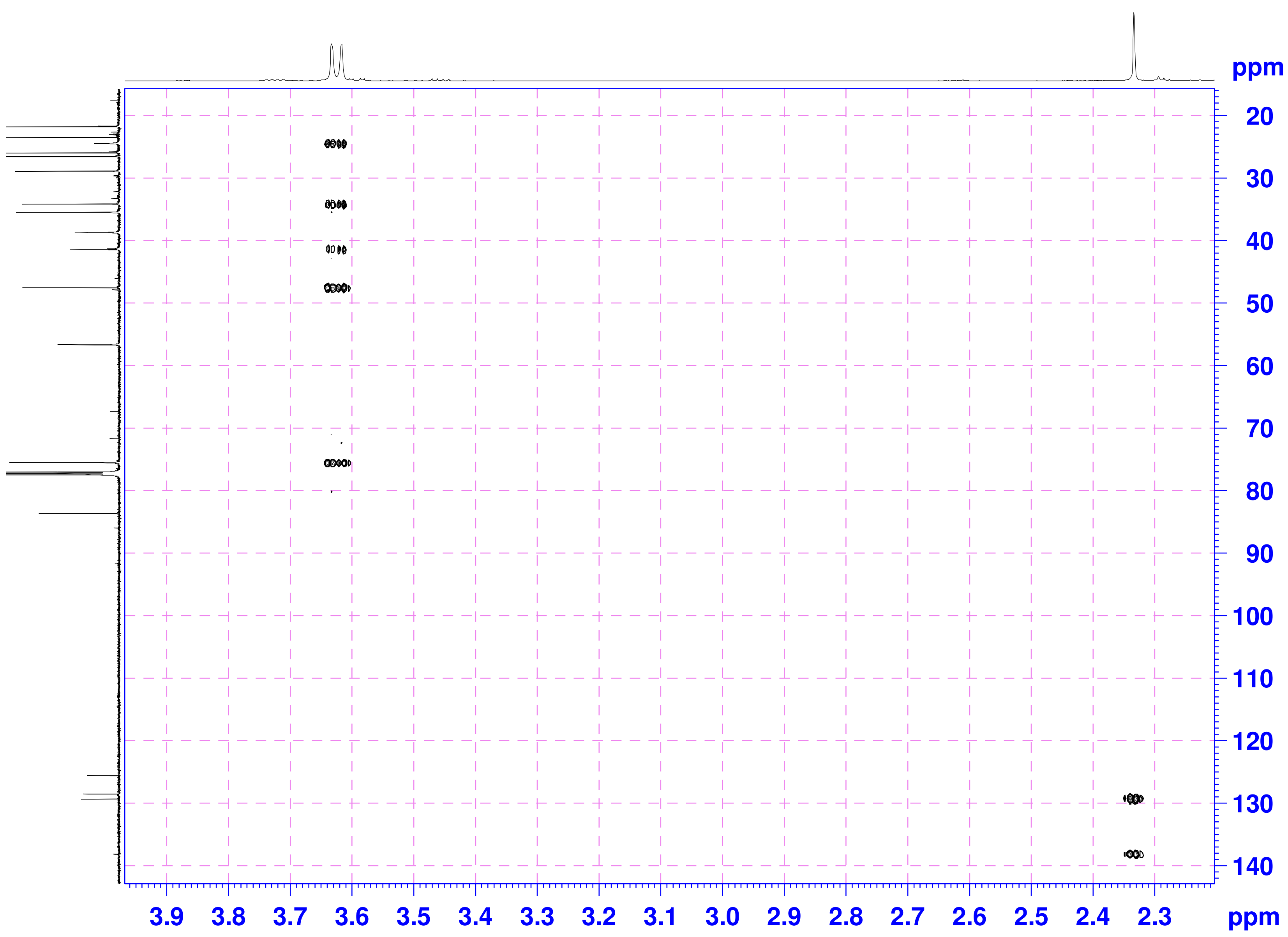


```
Current Data Parameters
NAME              CaroM3-2D
EXPNO                     9
PROCNO                    1

F2 - Acquisition Parameters
Date_              20220701
Time                  14.29
INSTRUM               spect
PROBHD   5 mm PABBO BB-
PULPROG      hmbcetgpl2nd
TD                     2048
SOLVENT               CDCl3
NS                       32
DS                       16
SWH                5000.000 Hz
FIDRES             2.441406 Hz
AQ                0.2048000 sec
RG                      203
DW                  100.000 usec
DE                    20.00 usec
TE                    298.8 K
CNST6           125.0000000
CNST7           165.0000000
CNST13           10.0000000
CNST30            0.5981151
D0               0.00000300 sec
D1               1.42627203 sec
D6               0.05000000 sec
D16              0.00020000 sec
IN0              0.00001490 sec

======== CHANNEL f1 ========
SFO1         600.1323288 MHz
NUC1                  1H
P1                 10.60 usec
P2                 21.20 usec
PLW1         27.82500076 W

======== CHANNEL f2 ========
SFO2         150.9178738 MHz
NUC2                 13C
P3                  8.80 usec
P24              2000.00 usec
PLW2         78.13500214 W
SPNAM[7]     Crp60comp.4
SPOAL7             0.500
SPOFFS7   0 Hz
SPW7          9.24489975 W

====== GRADIENT CHANNEL =====
GPNAM[1]         SINE.100
GPNAM[3]         SINE.100
GPNAM[4]         SINE.100
GPNAM[5]         SINE.100
GPZ1               80.00 %
GPZ3               15.00 %
GPZ4              -10.00 %
GPZ5               -5.00 %
P16              1000.00 usec

F1 - Acquisition parameters
TD                       256
SFO1             150.9179 MHz
FIDRES         131.082214 Hz
SW                222.353 ppm
FnMODE       Echo-Antiecho

F2 - Processing parameters
SI                      2048
SF            600.1300257 MHz
WDW                     SINE
SSB      0
LB       0 Hz
GB       0
PC                      1.40

F1 - Processing parameters
SI                       512
MC2             echo-antiecho
SF            150.9027314 MHz
WDW                     SINE
SSB                        2
LB       0 Hz
GB       0
```

CM3

13C HMBC spectra Dr.Orabi Caro M3 in CDCL3

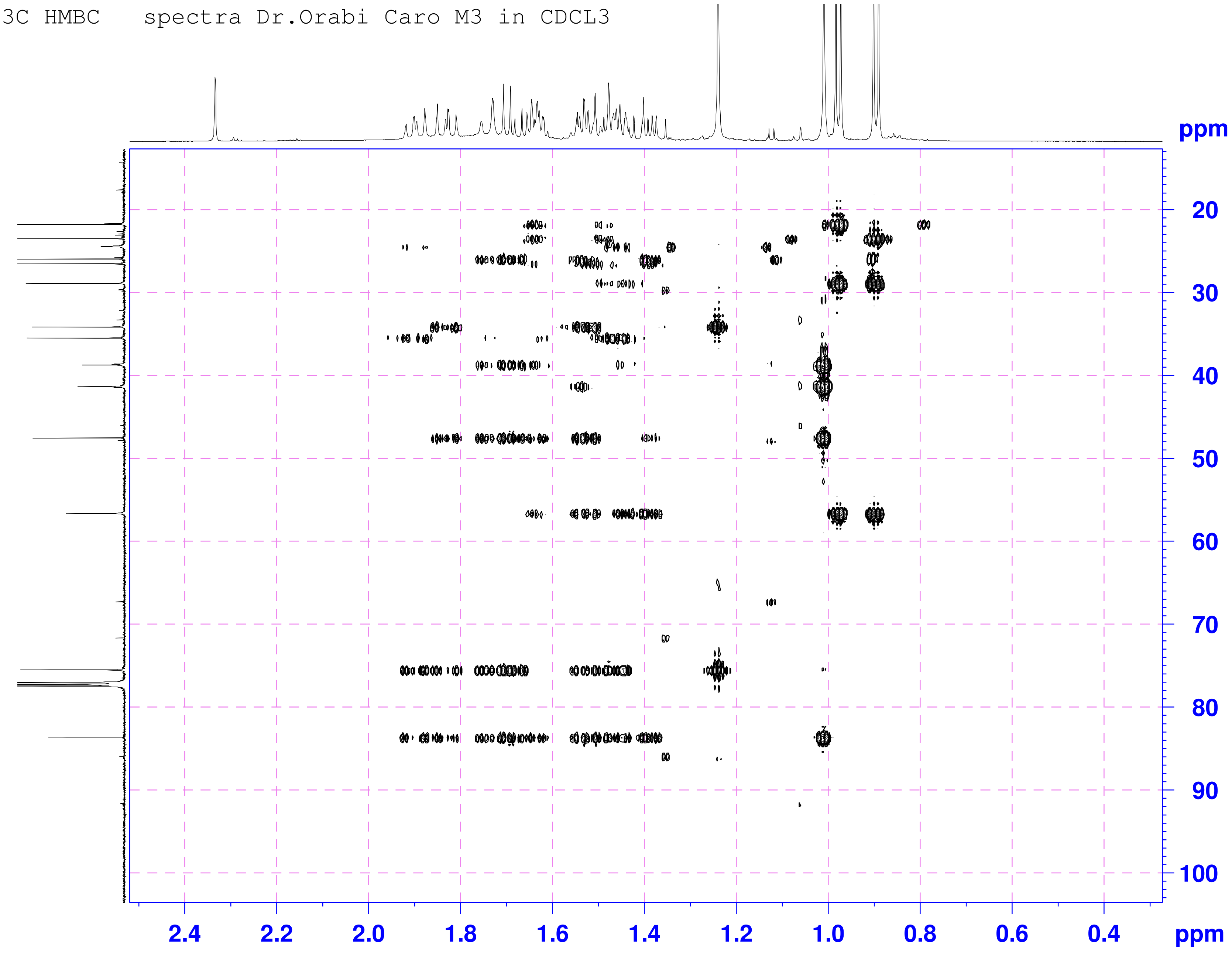


```
Current Data Parameters
NAME             CaroM3-2D
EXPNO                    9
PROCNO                   1

F2 - Acquisition Parameters
Date_             20220701
Time                 14.29
INSTRUM              spect
PROBHD   5 mm PABBO BB-
PULPROG      hmbcetgpl2nd
TD                    2048
SOLVENT              CDCl3
NS                      32
DS                      16
SWH               5000.000 Hz
FIDRES            2.441406 Hz
AQ               0.2048000 sec
RG                     203
DW                 100.000 usec
DE                   20.00 usec
TE                   298.8 K
CNST6          125.0000000
CNST7          165.0000000
CNST13          10.0000000
CNST30           0.5981151
D0              0.00000300 sec
D1              1.42627203 sec
D6              0.05000000 sec
D16             0.00020000 sec
IN0             0.00001490 sec

======== CHANNEL f1 ========
SFO1           600.1323288 MHz
NUC1                    1H
P1                   10.60 usec
P2                   21.20 usec
PLW1           27.82500076 W

======== CHANNEL f2 ========
SFO2           150.9178738 MHz
NUC2                   13C
P3                    8.80 usec
P24                2000.00 usec
PLW2           78.13500214 W
SPNAM[7]       Crp60comp.4
SPOAL7               0.500
SPOFFS7   0 Hz
SPW7            9.24489975 W

====== GRADIENT CHANNEL =====
GPNAM[1]          SINE.100
GPNAM[3]          SINE.100
GPNAM[4]          SINE.100
GPNAM[5]          SINE.100
GPZ1                 80.00 %
GPZ3                 15.00 %
GPZ4                -10.00 %
GPZ5                 -5.00 %
P16                1000.00 usec

F1 - Acquisition parameters
TD                     256
SFO1              150.9179 MHz
FIDRES          131.082214 Hz
SW                 222.353 ppm
FnMODE        Echo-Antiecho

F2 - Processing parameters
SI                    2048
SF             600.1300257 MHz
WDW                   SINE
SSB       0
LB        0 Hz
GB        0
PC                    1.40

F1 - Processing parameters
SI                     512
MC2           echo-antiecho
SF             150.9027314 MHz
WDW                   SINE
SSB                      2
LB        0 Hz
GB        0
```

CM3

COSY spectra Dr.Orabi Caro M3 in CDCL3

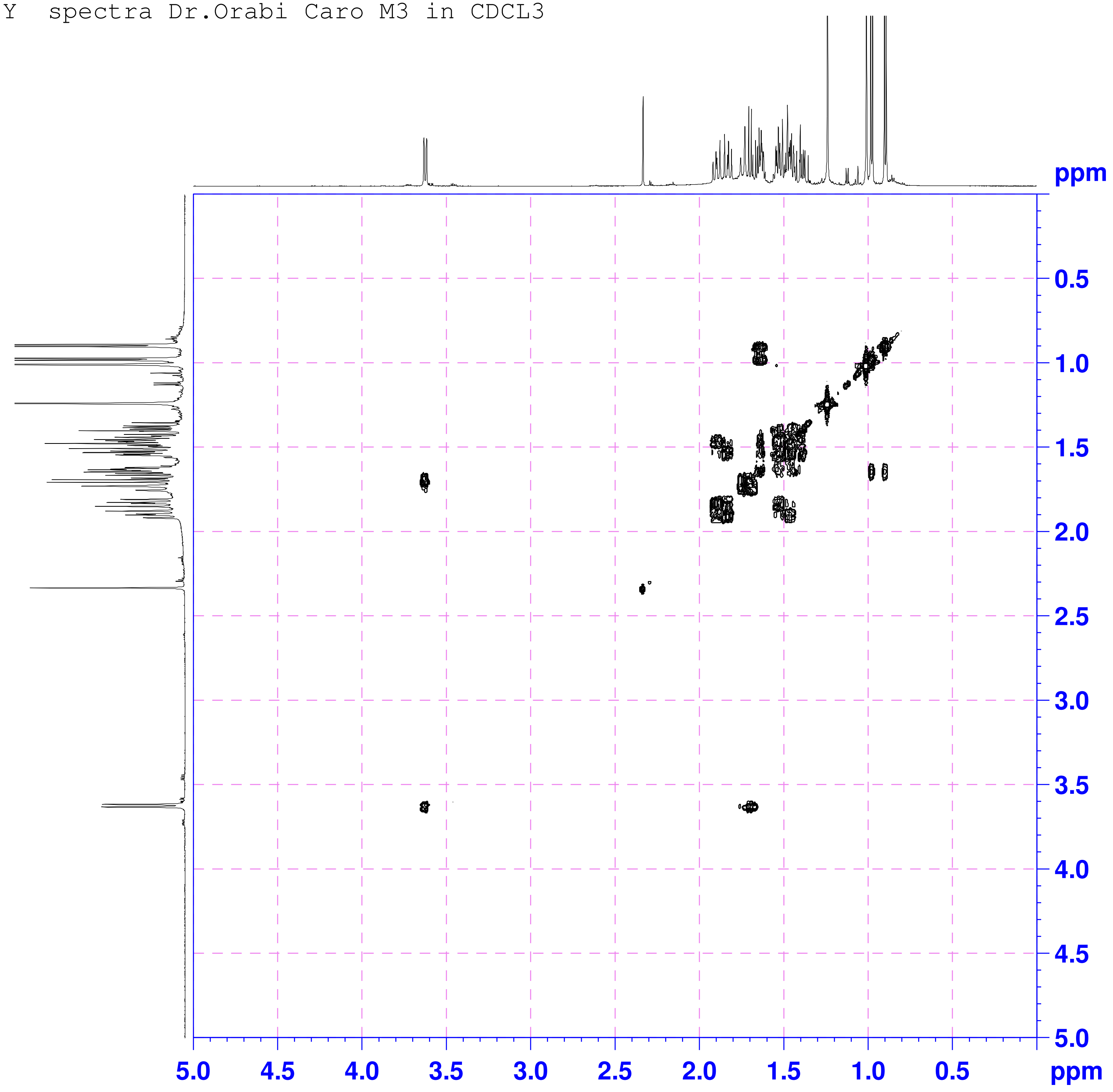


```
Current Data Parameters
NAME              CaroM3-2D
EXPNO                     7
PROCNO                    1

F2 - Acquisition Parameters
Date_              20220701
Time                   8.50
INSTRUM               spect
PROBHD     5 mm PABBO BB-
PULPROG          cosygpppqf
TD                     2048
SOLVENT               CDCl3
NS                        8
DS                        8
SWH                4854.369 Hz
FIDRES             2.370297 Hz
AQ                0.2109440 sec
RG                     90.5
DW                  103.000 usec
DE                    20.00 usec
TE                    298.8 K
D0               0.00000300 sec
D1               0.66685098 sec
D11              0.03000000 sec
D12              0.00002000 sec
D13              0.00000400 sec
D16              0.00020000 sec
IN0              0.00020600 sec

======== CHANNEL f1 ========
SFO1            600.1322713 MHz
NUC1                     1H
P0                    10.60 usec
P1                    10.60 usec
P17                 2500.00 usec
PLW1            27.82500076 W
PLW10            4.62489986 W

====== GRADIENT CHANNEL =====
GPNAM[1]           SINE.100
GPZ1                  20.00 %
P16                 1000.00 usec

F1 - Acquisition parameters
TD                      320
SFO1               600.1323 MHz
FIDRES            15.169903 Hz
SW                    8.089 ppm
FnMODE                   QF

F2 - Processing parameters
SI                     2048
SF              600.1300250 MHz
WDW                    SINE
SSB      0
LB       0 Hz
GB       0
PC                     1.40

F1 - Processing parameters
SI                     2048
MC2                      QF
SF              600.1300235 MHz
WDW                    SINE
SSB      0
LB       0 Hz
GB       0
```

CM3

COSY spectra Dr.Orabi Caro M3 in CDCL3

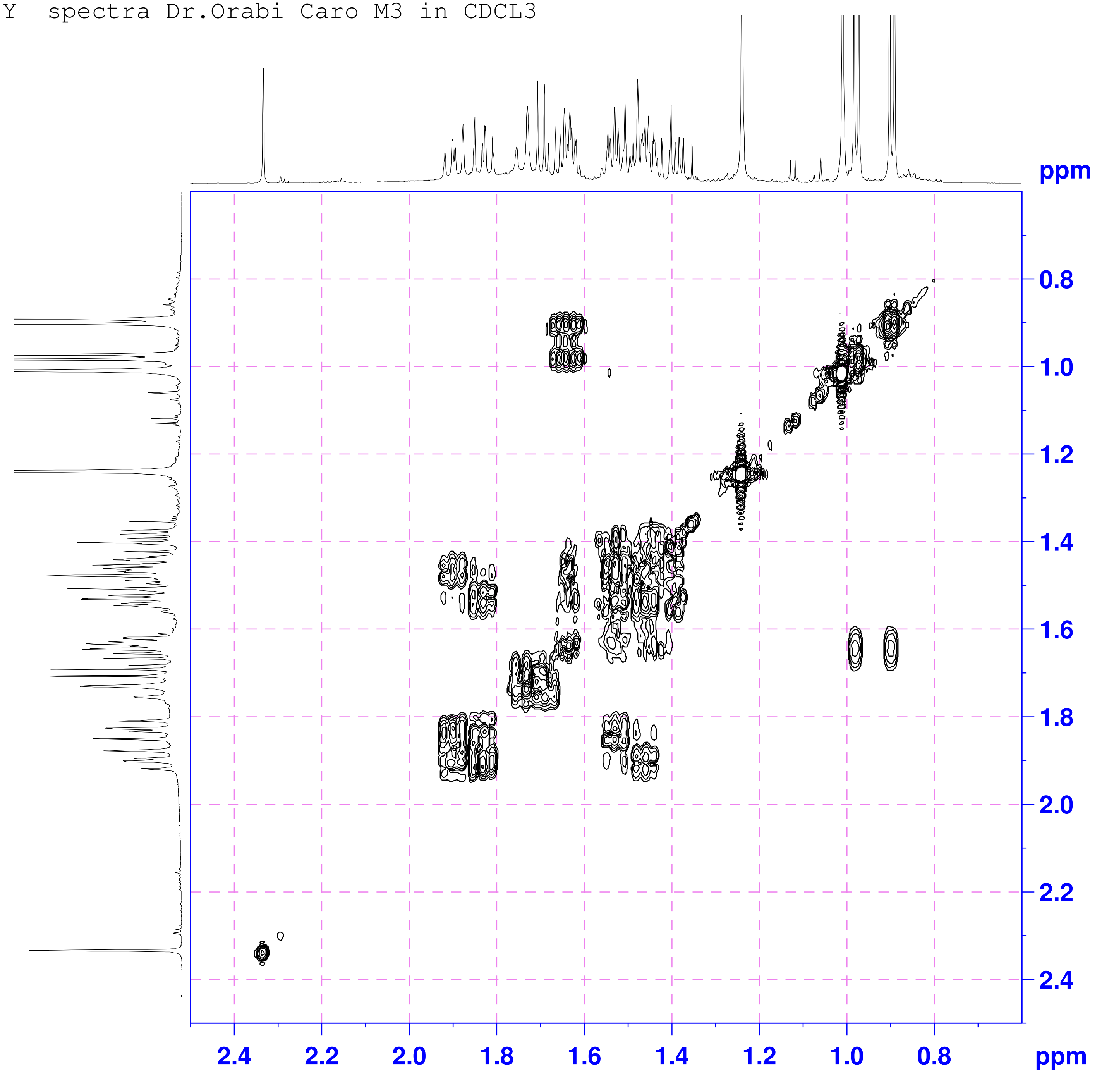


```
Current Data Parameters
NAME            CaroM3-2D
EXPNO                   7
PROCNO                  1

F2 - Acquisition Parameters
Date_            20220701
Time                 8.50
INSTRUM             spect
PROBHD     5 mm PABBO BB-
PULPROG        cosygpppqf
TD                   2048
SOLVENT             CDCl3
NS                      8
DS                      8
SWH              4854.369 Hz
FIDRES           2.370297 Hz
AQ              0.2109440 sec
RG                   90.5
DW                103.000 usec
DE                  20.00 usec
TE                  298.8 K
D0             0.00000300 sec
D1             0.66685098 sec
D11            0.03000000 sec
D12            0.00002000 sec
D13            0.00000400 sec
D16            0.00020000 sec
IN0            0.00020600 sec

======== CHANNEL f1 ========
SFO1          600.1322713 MHz
NUC1                   1H
P0                  10.60 usec
P1                  10.60 usec
P17               2500.00 usec
PLW1          27.82500076 W
PLW10          4.62489986 W

====== GRADIENT CHANNEL =====
GPNAM[1]         SINE.100
GPZ1                20.00 %
P16               1000.00 usec

F1 - Acquisition parameters
TD                    320
SFO1             600.1323 MHz
FIDRES          15.169903 Hz
SW                  8.089 ppm
FnMODE                 QF

F2 - Processing parameters
SI                   2048
SF            600.1300250 MHz
WDW                  SINE
SSB        0
LB         0 Hz
GB         0
PC                   1.40

F1 - Processing parameters
SI                   2048
MC2                    QF
SF            600.1300235 MHz
WDW                  SINE
SSB        0
LB         0 Hz
GB         0
```

Microbial Biotransformation of the Sesquiterpene Carotol: Generation of Hydroxylated Metabolites with Potential Cytotoxic and Target-Specific Binding Activities

Hanan G. Sary [1,2], Mohammed A. Khedr [1,3], Mohamed M. Radwan [4,5], Mickey Vinodh [6], and Khaled Y. Orabi [1*]

[1] Department of Pharmaceutical Chemistry, College of Pharmacy, Kuwait University, Safat 13110, Kuwait; hanan.sary@ku.edu.kw, mohammed.khedr@ku.edu.kw, ky.orabi@ku.edu.kw

[2] Department of Pharmacognosy, Faculty of Pharmacy, Ain-Shams University, Cairo, Egypt; hanangaber@pharma.asu.edu.eg

[3] Department of Pharmaceutical Chemistry, Faculty of Pharmacy, Helwan University, Ain Helwan, Cairo 11795, Egypt

[4] Department of Biomolecular Sciences, School of Pharmacy, The University of Mississippi, University, MS 38677, USA; mradwan@olemiss.edu

[5] National Center for Natural Products Research, School of Pharmacy, The University of Mississippi, University, MS 38677, USA; mradwan@olemiss.edu

[6] Research Sector Project Units, Faculty of Science, Kuwait University, Safat 13060, Kuwait; miky.findo@ku.edu.kw

* Correspondence: ky.orabi@ku.edu.kw; Tel.: +965-2463-6048

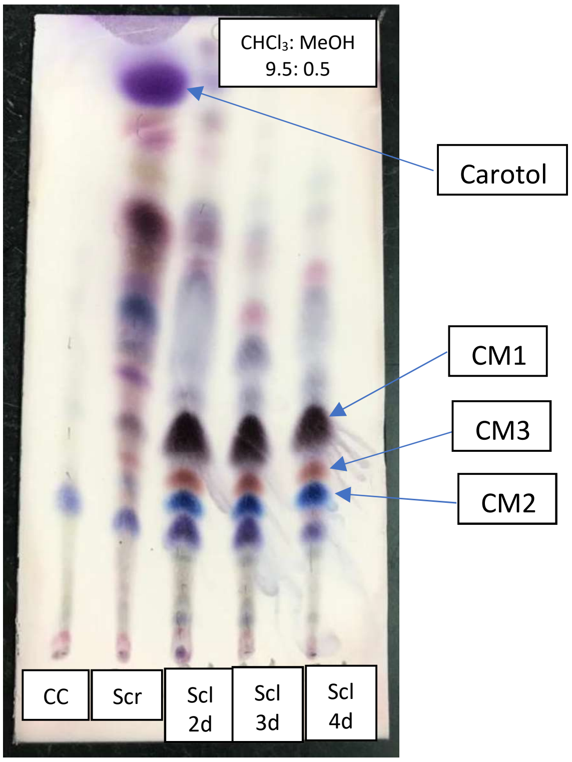

Figure S2. TLC monitoring of the metabolism on a daily basis. Visualization was performed using *p*-anisaldehyde/sulfuric acid followed by heating. Mobile phase: $CHCl_3$/MeOH (9.5:0.5).
Key: CC: culture control; Scr: screening culture; Scl: scale-up culture; d: days.

Microbial Biotransformation of the Sesquiterpene Carotol: Generation of Hydroxylated Metabolites with Potential Cytotoxic and Target-Specific Binding Activities

Hanan G. Sary [1,2], Mohammed A. Khedr [1,3], Mohamed M. Radwan [4,5], Mickey Vinodh [6], and Khaled Y. Orabi [1*]

[1] Department of Pharmaceutical Chemistry, College of Pharmacy, Kuwait University, Safat 13110, Kuwait; hanan.sary@ku.edu.kw, mohammed.khedr@ku.edu.kw, ky.orabi@ku.edu.kw
[2] Department of Pharmacognosy, Faculty of Pharmacy, Ain-Shams University, Cairo, Egypt; hanangaber@pharma.asu.edu.eg
[3] Department of Pharmaceutical Chemistry, Faculty of Pharmacy, Helwan University, Ain Helwan, Cairo 11795, Egypt
[4] Department of Biomolecular Sciences, School of Pharmacy, The University of Mississippi, University, MS 38677, USA; mradwan@olemiss.edu
[5] National Center for Natural Products Research, School of Pharmacy, The University of Mississippi, University, MS 38677, USA; mradwan@olemiss.edu
[6] Research Sector Project Units, Faculty of Science, Kuwait University, Safat 13060, Kuwait; miky.findo@ku.edu.kw
* Correspondence: ky.orabi@ku.edu.kw; Tel.: +965-2463-6048

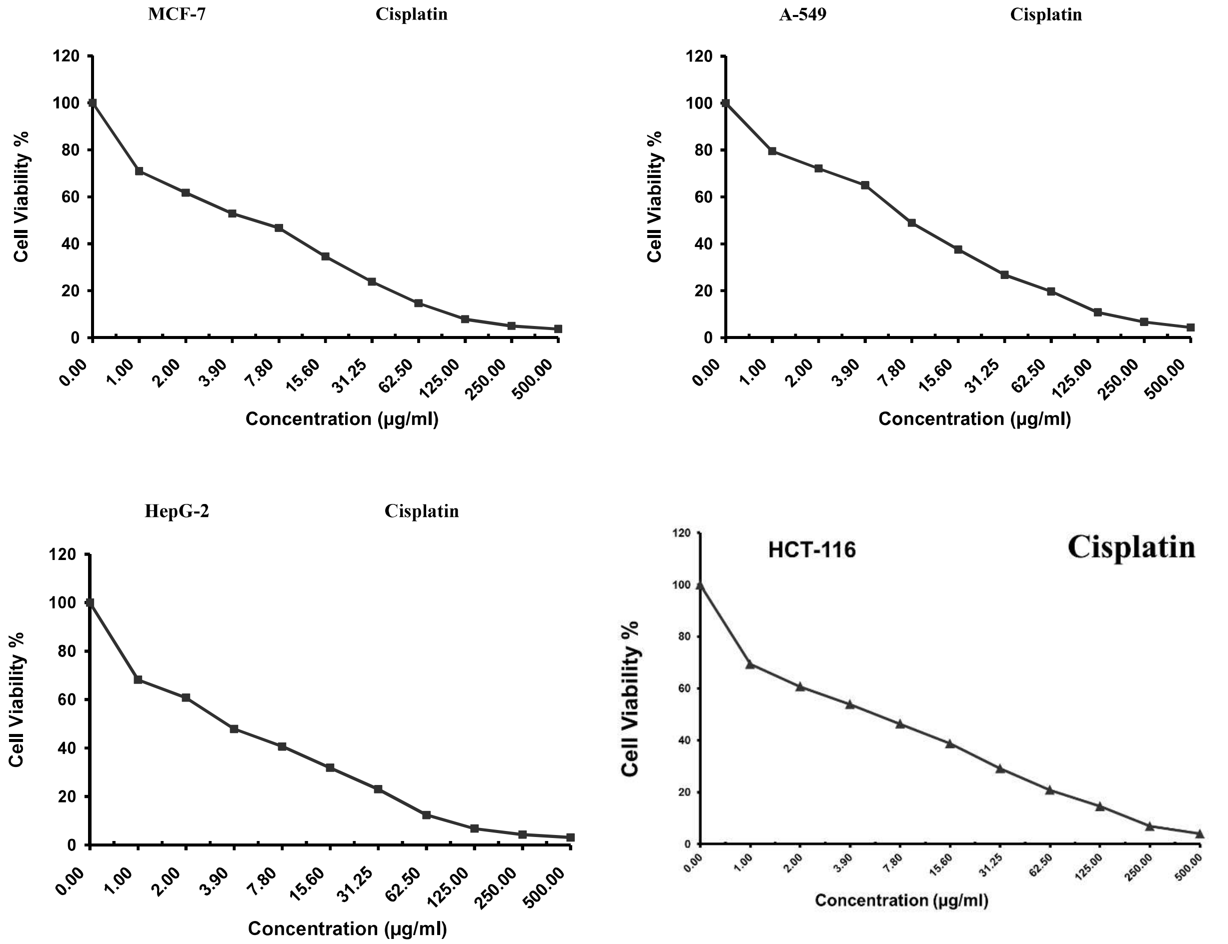

Figure S3. Dose-response curves of *cis*-platin against MCF-7 (breast carcinoma), A-549 (lung carcinoma), HepG-2 (hepatocellular carcinoma), and HCT-116 (colon carcinoma).
